# Association of 25(OH)-Vitamin D and metabolic factors with colorectal polyps

**DOI:** 10.1371/journal.pone.0286654

**Published:** 2023-06-08

**Authors:** Chih-Hsiang Chiang, Yu-Jun Chang, Sin-Ru He, Jih-Ning Chao, Chih-Huai Yang, Yen-Tze Liu

**Affiliations:** 1 Department of Family Medicine, Changhua Christian Hospital, Changhua, Taiwan; 2 Big Data Center, Changhua Christian Hospital, Changhua, Taiwan; 3 Institute of Statistics, National Chung Hsing University, Taichung City, Taiwan; 4 Department of Post-Baccalaureate Medicine, College of Medicine, National Chung Hsing University, Taichung, Taiwan; Health Sciences University, Faculty of Medicine, Bursa City Health Practice and Research Center, TURKEY

## Abstract

**Background:**

Studies have revealed the association of vitamin D with specific types of cancer development, however, its correlation with colorectal polyps (CRPs) remains unverified. Our study aimed to investigate the relationship between vitamin D levels, metabolic factors, and CRPs.

**Methods:**

A cross-sectional study from 2017 to 2019 involving 1306 participants was conducted to investigate the association among vitamin D levels, metabolic factors, uric acid and CRPs in Taiwan. CRPs diagnoses were determined via colonoscopies conducted by experienced gastrointestinal physicians, and biopsied polyps were inspected under a microscope by experienced pathologists. We employed both simple and multiple logistic regression analyses to identify significant factors associated with CRPs and adenomatous polyps, respectively.

**Results:**

Our result showed that the prevalence of 25(OH)-vitamin D deficiency (≦ 20 ng/mL) and CRPs was 21.21% and 40.89%, respectively. Multiple logistic regression revealed that the risk of CRPs increased with old age, male sex, hyperglycemia, high triglyceride levels, and low 25(OH)D levels after adjustment for other factors. Besides, low 25(OH)D levels were significantly associated with CRPs risk in women, whereas elevated blood pressure was associated with CRPs risk in men. 25(OH)D Deficiency was revealed to be significantly associated with risk of CRPs in adults over 50 years old. Compared to nonadenomatous polyps, older age, higher 25(OH) vitamin D and higher uric acid levels were at increased risk for adenomatous polyps.

**Conclusions:**

Our study revealed that vitamin D deficiency was significantly associated with the risk of CRPs, especially in adults over 50 years old and women. We should therefore be concerned about the CRP risk of vitamin D deficiency and metabolic syndrome (especially hyperglycemia, elevated blood pressure in men, and high triglyceride levels) in this population.

## Introduction

Colorectal cancer (CRC) is a common cancer for both men and women. It is the third most diagnosed cancer and second in terms of mortality worldwide in 2018 [1]. The threat of CRC has also increased in Taiwan in recent years. For the eleventh consecutive year, CRC was ranked first among the top 10 cancers in Taiwan in terms of the number of cases. As per the cancer registration statistics released by Taiwan’s Health Promotion Administration in 2018, CRC was ranked first and third among men and women, respectively, in terms of the incidence rate; in terms of the mortality rate, CRC was ranked third and fourth among men and women, respectively [2].

The risk factors for CRC comprise lifestyle factors (such as obesity, lack of exercise, excessive intake of red, animal fat or processed meat, low intake of natural fiber sources, and smoking) and nonmodifiable factors (such as age and personal or family history of CRC or adenoma) [3]. CRC usually develops from colorectal polyps (CRPs); therefore, clarifying the modifiable risk factors for CRPs and CRC should contribute toward their early prevention.

CRPs are polyps that develop on the lining of the colon or rectum. They can be categorized based on tissue characteristics into adenomatous (villous, tubulovillous, and tubular) and nonadenomatous (inflammatory and hyperplastic) polyps. Relative to nonadenomatous polyps, adenomatous polyps have a higher chance of becoming cancerous [4]. Most CRC growths undergo a slow transformation from adenomatous polyps through the adenoma–carcinoma sequence [4, 5]. Hyperplastic polyps are used to be considered benign, but recent studies suggest that some of them may progress and carry malignant potential through the serrated-polyp pathway [6, 7]. Studies of colorectal neoplasia mostly used the presence of adenomatous polyps as an index to clarify the risk factors involved in CRC initiation. Conversely, hyperplastic polyps were seldom included in such studies [8].

Vitamin D comprises a group of fat-soluble vitamins with multiple physiological functions. It is an essential nutrient for the normal growth and health of human beings [9]. Besides the maintenance of the calcium–phosphorus balance and skeletal health, recent studies have discovered that vitamin D provides many extraskeletal health benefits, and its deficiency may be associated with health conditions such as cardiovascular disease (CVD), chronic kidney disease, diabetes mellitus (DM), autoimmune disease, specific infections, and cancer development [9–12].

Studies conducted in western countries have demonstrated the protective role of vitamin D for CRPs and CRC [13]. However, other studies have also proposed that vitamin D is not related to CRC [14, 15]. One study even reported that Ca and vitamin D supplementation may increase the risk of sessile serrated adenomas or polyps [16]. The relationship between vitamin D and CRPs is still a topic of debate and more research is needed to clarify their true relationship.

Metabolic Syndrome (MetS) means a combination of multiple CVD risk factors including abdominal obesity, dyslipidemia, raised blood pressure, insulin resistance and proinflammatory and prothrombotic state [17]. It is a major public health challenge due to the increasing obesity rates and adoption of sedentary lifestyles.

Besides increasing the risk of atherosclerosis and cardiovascular disease, some studies also showed MetS increases the risks of some gastrointestinal disease such as CRC, gastric cancer, and colorectal adenomas [9, 18]. MetS and CRC risk factors that overlap include the excessive intake of oily foods, obesity, and insufficient physical activity. Studies have reported that patients with MetS were prone to develop adenomatous polyps or CRC [18, 19]; however, few studies have examined nonadenomatous polyps.

In view of above mentioned reasons, the present study examined the association among vitamin D, metabolic factors, and CRPs, including adenomatous and nonadenomatous polyps.

## Methods

### Study design and population

We conducted a cross-sectional study at the health examination center of Changhua Christian Hospital, Changhua City, Taiwan. Individuals who underwent a health examination with colonoscopy between 2017 and 2019 were enrolled.

A total of 3100 colonoscopy procedures were performed between 2017 and 2019. However, some participants received multiple colonoscopies during this 3-year period. For such participants, we used the data from their latest health report if no polyps were detected during their colonoscopies. However, if their colonoscopy reports were inconsistent, we then used the report that indicated the presence of CRPs or high-risk polyps. In total, 2679 participants were initially enrolled, after which we excluded the participants with missing data (data used as study variables); a total of 1723 participants were included in the analysis.

**Fig 1:**
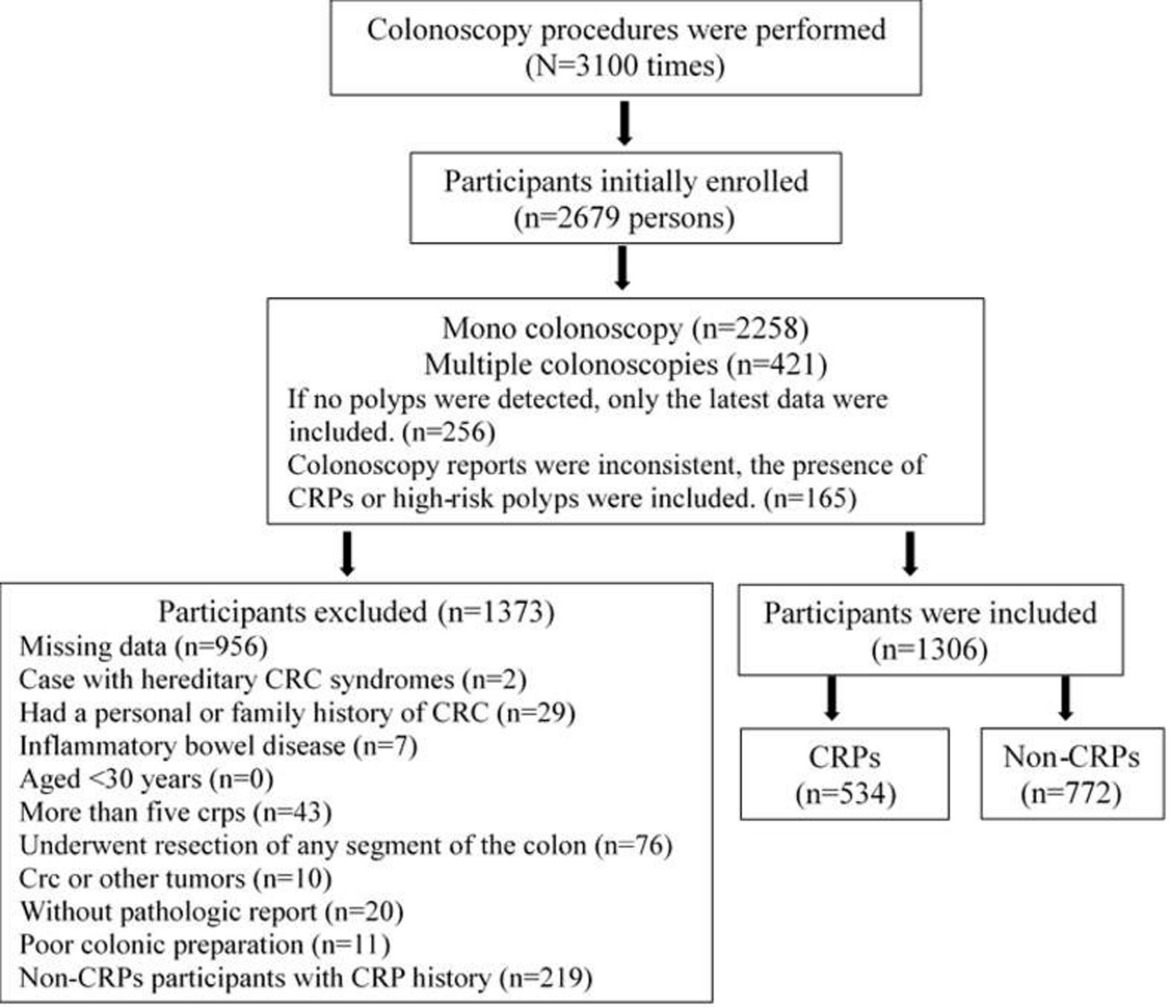
Flowchart of the study population and categorization.

We also excluded participants who had hereditary CRC syndromes (such as familial adenomatous polyposis), had a personal or family history of CRC, had inflammatory bowel disease (such as Crohn’s disease and ulcerative colitis), were aged <30 years, had more than five CRPs, underwent resection of any segment of the colon, had CRC or other tumors, did not have a pathologic report, or had poor colonic preparation. Non-CRPs participants with CRPs history were also excluded. A total of 1306 participants were enrolled for further analysis in the present study. (Fig 1)

### Data collection

These participants completed a questionnaire that collected demographic data on age, sex, alcohol consumption, and family and past history (including history of CRPs and CRC, DM, hypertension, and prior surgeries). They also received physical examinations to determine their body height, body weight, waist circumference, and blood pressure. Waist circumference (measured to the nearest 0.1 cm) was measured at the midpoint between the lower border of the rib and iliac crest. Blood pressure readings were obtained after the participants sat down and rested for 10 min.

We also collected blood samples from the participants after they had fasted overnight (>8 hours).

The data used in our analysis comprised age, sex, and levels of vitamin D, fasting plasma glucose (FPG), triglyceride (TG), total cholesterol (TC), high-density lipoprotein cholesterol (HDL-C), low-density lipoprotein cholesterol (LDL-C), and uric acid.

Biochemist markers, including FPG, HbA1C, TG, HDL-C, TC, LDL-C, and uric acid were measured using a Beckman Coulter AU analyzer (Brea, CA, USA) and included Timed-endpoint method (LDL, TG, HDL-C and uric acid), Cholesterol oxidase method (TC), Hexokinase-UV/NAD (FPG). HbA1c levels were measured using Ion-exchange High Pressure Liquid Chromatography via a Bio-Rad D-100 Analyzer.

### Diagnostic criteria

#### Measurement of 25-Hydroxyvitamin-D Concentration

Electrochemiluminescence immunoassay methods were used to measure total 25-hydroxyvitamin-D (25(OH)D), which is the main form of vitamin D found in the circulatory system; it is also commonly used to assess an individual’s vitamin D status because it has a half-life of up to 2 to 3 weeks, and undergoes fewer changes due to parathyroid hormones [9]. Total serum 25(OH)D was first analyzed as a continuous variable and then analyzed categorically using the previously defined thresholds used by most clinicians (deficiency, 25(OH)D ≦ 20 ng/mL; insufficiency, 25(OH)D of 21–29 ng/mL; normal level, 25(OH)D ≥ 30ng/mL) [12, 20].

#### Metabolic syndrome (MetS)

The modified criteria for MetS was defined as the presence of three or more of the following five conditions, according to the Taiwan’s Ministry of Health and Welfare [21, 22]: (1) abdominal obesity, which was defined as having a waist circumference of ≥90 and ≥80 cm for men and women, respectively; (2) hypertriglyceridemia, which was defined as having a TG level of ≥150 mg/dL; (3) low HDL-C, which was defined as having a serum HDL-C level of <40 and <50 mg/dL for men and women, respectively; (4) elevated blood pressure, which was defined as having a systolic blood pressure of ≥130 mmHg, diastolic blood pressure of ≥85 mmHg, or hypertension history (including self-reported or medical records); and (5) hyperglycemia, which was defined as having an FPG of ≥100 mg/dL or DM history (including self-reported or medical records).

#### Colorectal polyps (CRPs)

The diagnosis of CRPs was determined through colonoscopies conducted by experienced gastrointestinal physicians at the hospital, who were blinded to the objectives of the present study and the laboratory values. Lesions were removed through endoscopic resection (biopsy, polypectomy, or endoscopic mucosal resection), and then were biopsied and histologically assessed by experienced pathologists.

The pathology report would be confirmed after peer discussion if there were different opinions about the polyp types. Based on the pathological biopsy reports, CRPs were then categorized as adenomatous (tubular, tubulovillous, villous, serrated or high-grade dysplastic changes) polyps and nonadenomatous (inflammatory or hyperplastic) polyps. If participants had adenomatous and nonadenomatous polyps at the same time, they were assigned to the adenomatous polyp group. If the patient had more than one histological type of lesion, the grouping was based on the neoplasia with the highest risk. Polyp size data were not analyzed in this study due to inconsistencies in polyp size descriptions.

### Statistical analyses

Independent t-test was applied to compare the continuous variables of the CRPs and non-CRPs groups. Next, Age, gender, 25(OH)D, uric acid and metabolic factors were further separated into categorical variables to identify the risk factors for CRPs. Simple logistic regression was then used to explore risk factors and calculate the odds ratio for CRPs. The result was considered statistically significant for P < 0.05. Since gender and age were modifiers and may interact with other variables, thus we further performed multiple logistic regression analyses with stratification to identify the variables significantly associated with CRPs. Finally, we used both simple and multiple logistic regression to compare differences in adenomatous polyp risk by age, sex, 25(OH)D, uric acid, and metabolic factors. All statistical analyses were conducted by using IBM SPSS Statistics for Windows, Version 22.0 (IBM Corp., Armonk, NY).

### Ethical approval

Ethical approval for this study was obtained from The Institutional Review Board of the Changhua Christian Hospital (CCH IRB No.191001). Owing to the retrospective design of the study and the use of de-identified patient information, the review board waived the need for written informed consent.

## Results

Among the 1306 participants, the prevalence of vitamin D deficiency (≦20 ng/mL) was 21.21% (21.72% and 20.85% in the CRPs and non-CRPs groups, Table 2), and the prevalence of CRPs was 40.89%. Significant differences between the CRPs and non-CRPs groups were observed for the means of age, waist circumference, body mass index (BMI), systolic blood pressure, diastolic blood pressure, FPG (fasting plasma glucose), HbA1C, HDL-C, TG and uric acid (Table 1).

**Table 1:** Mean differences in continuous variables between CRPs and non-CRPs Groups^#^.

	CRPs (*n* = 534)		Non-CRPs (*n* = 772)		
	Mean	SD	Mean	SD	P-value
Age	53.18	10.62	48.34	10.83	< 0.001
Waist circumference	84.34	10.61	79.30	10.19	< 0.001
BMI	25.25	3.68	23.98	3.56	< 0.001
Systolic BP	125.38	15.46	120.93	16.28	< 0.001
Diastolic BP	77.67	10.09	74.52	10.04	< 0.001
FPG*	101.02	23.35	94.00	18.06	< 0.001
HbA1c	5.64	0.90	5.42	0.65	< 0.001
Total cholesterol (TC)	199.91	39.26	201.39	38.39	0.499
HDL-C	48.52	14.38	52.89	14.44	< 0.001
LDL-C	126.09	34.86	126.89	34.59	0.684
Triglyceride (TG)	129.14	81.56	105.77	80.24	< 0.001
Uric acid	6.10	1.40	5.72	1.43	< 0.001
25(OH)D	28.67	10.45	27.82	9.59	0.128

#statistical test: Independent sample Student’s *t*-test

* FPG: fasting plasma glucose; CRPs: colorectal polyps.

Table 2 shows the crude (non-controlled) risk results obtained through univariate analysis. Age ≥ 65, male, abdominal obesity, elevated blood pressure, lower HDL-C levels, higher TG levels, and hyperglycemia were all associated with a high risk of CRPs. The overall odds ratio (OR) of MetS for CRPs was 2.50 (95% confidence interval [CI] = 1.95–3.21). However, 25(OH)D level had no statistical difference in risk for CRPs. Among the aforementioned factors, hyperglycemia exhibited the highest risk of CRPs. (OR = 2.50, 95% CI = 1.98–3.17).

**Table 2:** Simple logistic regression analysis of the factors and the risk for CRPs.

		CRP			Bivariable analysis (crude)		
		Total	N	%	Odds ratio	95% CI	P-value
Age (year)	> = 65	147	88	59.9	2.384	1.680–3.385	<0.001
	<65	1159	446	38.5	1.000		
Gender	Male	709	357	50.4	2.407	1.914–3.026	<0.001
	Female	597	177	29.6	1.000		
Abdominal obesity (cm)	Larger (M> = 90 & F> = 80)	409	193	47.2	1.457	1.150–1.845	0.002
	Normal (M<90 & F<80)	897	341	38.0	1.000		
Elevated blood pressure	Higher	600	282	47.0	1.598	1.279–1.995	<0.001
	Normal	706	252	35.7	1.000		
Lower HDL-C (mg/dL)	Lower (M<40 & F<50)	415	197	47.5	1.486	1.174–1.880	0.001
	Normal (M> = 40 & F> = 50)	891	337	37.8	1.000		
Higher TG (mg/dL)	Highter (> = 150)	295	168	56.9	2.331	1.791–3.035	<0.001
	Normal (<150)	1011	366	36.2	1.000		
Hyperglycemia	Higher	434	242	55.8	2.504	1.977–3.170	<0.001
	Normal	872	292	33.5	1.000		
MetS	Yes	351	201	57.3	2.503	1.950–3.213	<0.001
	No	955	333	34.9	1.000		
Uric acid (mg/dL)	Higher (> = 7.5)	183	82	44.8	1.205	0.880–1.651	0.245
	Normal (<7.5)	1123	452	40.2	1.000		
25(OH)D (ng/mL)	Abnormal (< = 20)	277	116	41.9	1.053	0.805–1.378	0.706
	Normal (>20)	1029	418	40.6	1.000		

CRPs: colorectal polyps MetS: Metabolic Syndrome

Multiple logistic regression was performed to further determine which factors were associated with CRPs in the overall population as well as separately in male and female subgroups. After multicollinearity diagnostics, we excluded abdominal obesity, HDL-C and uric acid because they had high correlation with other variables. The result revealed that old age (age ≥ 65), male sex (OR = 2.11, 95% CI = 1.64–2.72), hyperglycemia, high TG levels, and low 25(OH)D levels were associated with increased risk of CRPs after adjustment for other factors (Table 3).

**Table 3:** Multiple logistic regression analysis of CRPs by gender.

		Overall (n = 1306)			Male (n = 709)			Female (n = 597)		
		OR	95% CI	P-value	OR	95% CI	P-value	OR	95% CI	P-value
Age	> = 65	2.178	1.490–3.185	<0.001	2.191	1.298–3.697	0.003	2.360	1.341–4.155	0.003
	<65	1.000			1.000			1.000		
Gender	Male	2.112	1.642–2.717	<0.001						
	Female	1.000								
Blood pressure*	Higher	1.214	0.955–1.543	0.112	1.398	1.027–1.902	0.033	0.964	0.653–1.423	0.854
	Normal	1.000			1.000			1.000		
Hyperglycemia**	Higher	1.922	1.492–2.476	<0.001	1.797	1.298–2.488	<0.001	2.044	1.357–3.077	0.001
	Normal	1.000			1.000			1.000		
Triglyceride	> = 150	1.682	1.262–2.243	<0.001	1.452	1.048–2.011	0.025	2.832	1.550–5.177	0.001
	<150	1.000			1.000			1.000		
25(OH)D***	< = 20	1.486	1.108–1.992	0.008	1.276	0.821–1.982	0.278	1.653	1.112–2.457	0.013
	>20	1.000			1.000			1.000		

OR: Odds ratio; CRPs: colorectal polyps.

Each model was adjusted for potentially confounders: age, blood pressure, hyperglycemia, and triglyceride.

*Higher: systolic blood pressure ≥130 mmHg, diastolic blood pressure ≥85 mmHg, or hypertension history

**Higher: FPG of ≥100 mg/dL or DM history

***Abnormal: 25(OH)D ≤ 20 ng/mL

Since the correlation may not be the same in different genders, we further performed stratified analysis to identify their differences. The analysis showed that women were at higher risk of CRPs than men in terms of age, hyperglycemia and TG. Besides, low 25(OH)D levels were significantly associated with CRPs risk in women, whereas elevated blood pressure was associated with CRPs risk in men. (Table 3).

Next, we further divided participants into three age groups (age < 50, 50 ≤ age < 65, age ≥ 65) to determine the association between 25(OH)D and CRPs. After controlling for possible confounding factors, the results showed that 25(OH)D Deficiency (≤ 20 ng/mL) was significantly associated with risk of CRPs in adults over 50 years old. (50 years ≤ age < 65 years, OR = 1.81, 95% CI = 1.12–2.92, P = 0.016; age ≥ 65 years, OR = 6.31, 95% CI = 1.28–31.23, P = 0.024) (Table 4).

**Table 4:** Prevalence and odds ratio of CRPs associated with 25(OH)D in logistic regression analysis, as stratified by age.

		CRPs			Bivariable analysis (crude)			Multivariable analysis (adjusted)*		
Age	25(OH)D (ng/mL)	Total	N	%	OR	95% CI	P-value	OR	95% CI	P-value
<50	Abnormal (≤ 20)	165	53	32.1	1.012	0.691–1.483	0.951	1.249	0.832–1.875	0.284
	Normal (>20)	452	144	31.9	1.000			1.000		
50–64	Abnormal (≤ 20)	96	49	51.0	1.282	0.825–1.994	0.270	1.807	1.118–2.918	0.016
	Normal (>20)	446	200	44.8	1.000			1.000		
> = 65	Abnormal (≤ 20)	16	14	87.5	5.392	1.178–24.686	0.030	6.310	1.275–31.225	0.024
	Normal (>20)	131	74	56.5	1.000			1.000		

OR: Odds ratio; CRPs: colorectal polyps.

*Each model was adjusted for potentially confounders: gender, blood pressure, hyperglycemia, and triglyceride.

General speaking, adenomatous polyps have a higher chance of becoming cancerous than nonadenomatous polyps [4], so we also further classified CRPs as either adenomatous (n = 304) or nonadenomatous polyps (n = 230) to identify the differences between these two types of polyps. Multiple logistic regression revealed that age≥ 65 years, high uric acid levels were associated with increased risk of adenomatous polyps; in contrast, a serum 25(OH)D level ≤ 20 ng/mL was significantly (P = 0.01) associated with increased risk of nonadenomatous polyps than adenomatous polyps. (Table 5).

**Table 5:** Prevalence and odds ratio of adenomatous polyps in CRPs group.

			Adenomatous polyps			Bivariable analysis (crude)			Multivariable analysis (adjusted)	
	With CRPs	Total	N	%	OR	95% CI	P-value	OR	95% CI	P-value
Age (year)	> = 65	88	60	68.2	1.774	1.091–2.884	0.021	1.742	1.066–2.846	0.027
	<65	446	244	54.7	1.000			1.000		
Gender	Male	357	216	60.5	1.549	1.078–2.228	0.018			
	Female	177	88	49.7	1.000					
Abdominal obesity (cm)	Larger (M> = 90/F> = 80)	193	115	59.6	1.186	0.829–1.696	0.351			
	Normal (M<90/F<80)	341	189	55.4	1.000					
Elevated blood pressure	Higher	282	164	58.2	1.112	0.789–1.567	0.545			
	Normal	252	140	55.6	1.000					
Lower HDL-C (mg/dL)	Lower (M<40/F<50)	197	108	54.8	0.873	0.612–1.244	0.452			
	Normal (M> = 40/F> = 50)	337	196	58.2	1.000					
Higher TG (mg/dL)	Highter (> = 150)	168	102	60.7	1.255	0.865–1.82	0.232			
	Normal (<150)	366	202	55.2	1.000					
Hyperglycemia	Higher	242	137	56.6	0.977	0.692–1.378	0.893			
	Normal	292	167	57.2	1.000					
Metabolic syndrome	Yes	201	120	59.7	1.200	0.841–1.711	0.315			
	No	333	184	55.3	1.000					
Uric acid (mg/dL)	Higher (> = 7.5)	82	58	70.7	2.024	1.215–3.372	0.007	1.972	1.178–3.301	0.010
	Normal (<7.5)	452	246	54.4	1.000			1.000		
25(OH) D (ng/mL)	Abnormal (< = 20)	116	54	46.6	0.585	0.387–0.885	0.011	0.623	0.410–0.947	0.027
	Normal (>20)	418	250	59.8	1.000			1.000		

OR: Odds ratio; CRPs: colorectal polyps.

## Discussion

Colorectal adenomas are generally considered as precursors of cancer. Muto et al. reported that the rates at which tubular adenomas, intermediate-type adenomas, and villous adenomas progress to CRC are 4.8%, 22.5%, and 40.7%, respectively [4, 23]. Recent studies have also suggested the malignant potential of nonadenomatous polyps [6, 7, 24]. Therefore, our study focused on all types of CRPs. Some studies have reported that CRPs are detected in up to 33% of colonoscopies, and that two-thirds of all CRPs are adenomas [25, 26]. In our study, CRPs were detected in 40.89% of colonoscopies and 56.93% of all detected CRPs were adenomas.

The prevalence of vitamin D deficiency is increasing, and it has become a public health problem [20]. Definite standards for defining vitamin D deficiency have not yet been established. The Institute of Medicine (IOM) of the United States recommends an ideal serum 25(OH)D concentration of 20–50 ng/mL [27]. The International Osteoporosis Foundation (IOF) recommends a serum concentration of at least 30 ng/mL, particularly for older adults. For nonskeletal health, no recommended standards have been established [9]. According to the IOM Recommended Dietary Allowance (RDA) for adults, a daily intake of 600 units of vitamin D results in a blood 25(OH)D concentration of 20 ng/ml in at least 97.5% people [28]. An article in 2016 showed that the prevalence of vitamin D deficiency is overestimated by using the concentration of 20 ng/ml [29]. However, based on many considerations, such as bone health, parathyroid hormone concentrations, variation of different test methods, most clinicians, including endocrine society, define vitamin D deficiency and insufficiency as having 25(OH)D blood concentrations of ≤20 and 21–29 ng/mL, respectively. The normal blood concentration of vitamin D is ≥30 ng/mL [9, 12, 20]. The Nutrition and Health Survey in Taiwan, which surveyed 3755 Taiwanese adults between 2013 and 2016, revealed the average concentration of 25(OH)D was 29 ng/mL; in that survey, 18.6% of respondents had a vitamin D level of <20 ng/mL [30]. Our study indicated that 21.21% of the participants aged >30 years had a vitamin D level of ≤20 ng/mL, with the average concentration of 25(OH)D being 28.17 ng/mL. Therefore, our findings correspond to the current evidence from studies in Taiwan.

Studies have indicated a relationship between vitamin D deficiency and the occurrence of colorectal adenoma or CRC [31–33]. The World Health Organization’s International Agency for Research on Cancer concluded that a strong link exists between vitamin D level and both the incidence and survival rate of CRC [13, 15]. Our univariate analysis indicated that the 25(OH)D level was not associated with CRPs; however, a low 25(OH)D level was revealed to increase the risk of CRPs after adjustment for other factors (OR = 1.49; 95% CI = 1.11–1.99). Our results are similar to those of a 2013 study, which suggested that 25(OH)D level was not a significant predictor of the risk of colon adenoma; but appear to be a stronger association after adjustment for additional known risk factors [14, 15]. A systemic review study concluded that vitamin D level is inversely associated with CRC prevalence in Asian population and it suggested the need for Asians to maintain higher blood circulating vitamin D levels [33]. Our study found vitamin D level is inversely associated with all CRPs. Therefore, we also encouraged people to avoid Vitamin D level deficiency to lower the risk or CRPs. Cells that can cause carcinogenesis (such as skin, breast tissue, and colon cells) have vitamin D receptors (VDRs). Vitamin D can play an anticancer role through VDRs, and it also has an antiproliferation effect on cancer cells. Furthermore, Vitamin D has the functions of preventing angiogenesis and inflammation and repairing deoxyribonucleic acid (i.e., DNA). Collectively, these effects have a preventive effect on cancer development [9, 34, 35].

Our study suggests that vitamin D deficiency, especially in adults over 50 years old, was significantly associated with the risk of CRPs. In adults over 65 years old, the association is even more significant with odds ratio up to 6.31. Our study also revealed women with lower 25(OH)D level were at a significantly increased risk of CRPs (OR = 1.65; 95% CI = 1.11–2.46). The association was not significant in men. An international pooling project of 17 cohorts in 2017 found an inverse association between circulating 25(OH)D and colorectal cancer risk in both women and men, but a provocative finding of that study is the stronger association observed in women, however, the biological explanation is still unclear [31]. Further studies are required to clarify the relationship between vitamin D and CRPs in both sexes.

The prevalence of MetS is increasing worldwide due to increasing obesity and the adoption of sedentary lifestyles. The main components of MetS comprise obesity, hyperlipidemia, and hyperglycemia, which many studies have suggested to be associated with colorectal neoplasm [36–38]. Our study also suggested individuals with MetS have a significantly increased risk of CRPs (OR = 2.50, 95% CI = 1.95–3.21) in simple logistic regression. The pathological mechanism of MetS causing CRC is not fully understood, it may be mediated by the dysregulation of growth signals such as insulin growth factor I (IGF-I), cytokines, and vascular integrity factors, which contribute to cancer-related processes [39]. Furthermore, chronic low-grade inflammation caused by MetS tends to lead to colorectal tumor development [40].

Obesity is associated with a high risk of CRC [38, 41], and abdominal obesity and visceral adipose tissue increased the risk of adenomatous colonic polyps [42]. A previous study revealed that abdominal obesity is associated with a higher risk of CRC than BMI because it indicates a high visceral-fat level, which suggests insulin resistance and high IGF-I circulating levels [43]. Our univariate analysis indicated that abdominal obesity was associated with CRPs; however, it was excluded in multiple variable analysis after multicollinearity diagnostics. Therefore, further investigations are required to clarify the relationship between abdominal obesity and CRPs.

Studies have reported that an association between type 2 DM and an increased risk of CRPs and CRC [44–46]; similarly, our study revealed that after adjustment for other metabolic factors, hyperglycemia was associated with CRPs risk for all participants (OR = 1.92, 95% CI = 1.49–2.48). The positive relationship between type 2 DM and CRC may be related to the prolonged and high levels of circulating insulin and high endogenous insulin production, which were revealed by large clinical studies to be directly related to increased CRC risk [46, 47]. High insulin levels can also promote tumor growth [44, 47]. Insulin resistance or an increased IGF-I level may contribute to the adenoma–carcinoma process. Diabetes may lead to slow down gastrointestinal motility, which increases the likelihood of colonic mucosa exposure to potential carcinogens [44, 45].

Studies have discovered that patients with adenomatous polyps have higher serum TG and lower HDL-C levels relative to controls [48, 49]. Our univariate analysis indicated that higher TG and lower HDL-C level were at a higher risk of CRPs. Higher TG level was also revealed to increase risk of CRPs in both sexes after adjustment for other factors. Several factors may contribute to the association of serum TG and HDL-C with colorectal neoplasm. Low HDL-C and high TG levels may increase the levels of proinflammatory cytokines such as interleukin-6, tumor necrosis factor- α, and decrease the levels of anti-inflammatory cytokines such as interleukin-10 [50]. Inflammation is linked to DNA damage and the growth, apoptosis, and proliferation of colorectal tumors [38, 39]. Low HDL-C causes LDL-C to become more oxidized, thereby causing increased intracellular oxidative stress. Increased serum TG may also result in oxidative stress and the development of reactive oxygen species [39, 51], and the consequent loss of anti-oxidant activity caused by low HDL-C and high TG states then contributes to cancer development [52].

Our multiple logistic regression indicated that male participants with elevated blood pressure were more likely to have CRPs than those with a normal blood pressure (OR = 1.40, 95% CI = 1.03–1.90). Studies also reported positive associations of hypertension with CRPs and CRC [53, 54]. Another study concluded men having hypertension increased the risk of CRC by 13% (95% CI = 1.06–1.20) [54]. The mechanism by which abnormal blood pressure affects CRPs has not been clarified. Insulin resistance may be the primary mechanism that contributes to the parallel relationship between the progression of blood pressure and CRPs because hypertension and CRPs share the same mechanism with respect to insulin resistance [53].

In our study, uric acid level was excluded in multiple analysis for the risk for CRPs after multicollinearity diagnostics. Studies have indicated that uric acid may be related to CRC. Lower serum uric acid levels may decrease systemic inflammation and prevent polyp–cancer transformation [55, 56]. However, some studies have suggested that an increased serum uric acid level has protective effects against cancer development; hyperuricemia could clear free radicals and inhibit lipid and protein peroxidation [57, 58]. Notably, since uric acid plays dual roles as antioxidant and proinflammatory effects, the association between uric acid and CRPs requires further clarification.

When comparing the difference between adenomatous polyp and nonadenomatous polyp groups, our multiple logistic regression indicated old age (≥ 65 years) and high uric acid levels were at increased risk of adenomatous polyps and a serum 25(OH)D level ≤ 20 ng/mL increased more risk of nonadenomatous polyps than adenomatous polyps. A previous study suggested that the inverse association between circulating 25(OH)D and adenomas does not apply to hyperplastic polyps [8]. However, another study in 2021 did not find evidence for an association between vitamin D and overall colorectal polyps, but observed a trend for decreased odds of hyperplastic polyps with increased vitamin D levels [59]. The research variables of the above study were quite different from our study, which suggested that Vitamin D deficiency increased the risk of CRPs, especially nonadenomatous polyps. Besides, most previous studies focus on adenomas and hyperplastic polyps, and used no polyps group as control group. Our study directly compared the differences between adenomatous and nonadenomatous polyps in vitamin D and metabolic factors by using nonadenomatous polyps as reference population. More studies are required to verify the differences between adenomatous and nonadenomatous polyps.

To the best of our knowledge, the present study is first to explore the association of circulating vitamin D and metabolic factors with CRPs, including adenomatous and nonadenomatous polyps in the same population. The present study was subject to several limitations. First, the study design was retrospective and cross-sectional. Second, the participants enrolled in the present study were individuals who underwent a health check-up, which indicated that they were more health conscious and of a higher socioeconomic status than the general population; therefore, the results could be affected by selection bias. Approximately 40.89% of these participants had CRPs, which is a slightly higher prevalence level relative to those reported in other studies conducted in Asian countries [42, 50]. Furthermore, specific recognized CRC or CRPs risk factors such as lack of exercise and dietary habits were not analyzed in the present study. Therefore, further research is required to clarify the causal association of Vitamin D and MetS with CRPs.

## Conclusions

This study suggests that lower vitamin D levels and MetS are associated with increased risk for CRPs in Taiwanese adults. Risk of CRPs increased with old age, male sex, lower 25(OH) vitamin D levels, hyperglycemia, higher TG levels. Vitamin D deficiency was significantly associated with the risk of CRPs, especially in adults over 50 years old and women. Given the significant association we found between vitamin D deficiency and CRP risk, as well as the correlation between metabolic syndrome and CRP risk (especially hyperglycemia, elevated blood pressure in men, and high triglyceride levels), it is important to address these risk factors in the population we studied. Compared to nonadenomatous polyps, older age, higher 25(OH) vitamin D and higher uric acid levels were at increased risk for adenomatous polyps.

## Supporting information

S1 Data (DOCX)
