# Supplementary material for: Association of 25(OH)-Vitamin D and metabolic factors with colorectal polyps
Source: PLoS One. 2023 Jun 8;18(6):e0286654. doi: 10.1371/journal.pone.0286654 (PMC10249833; doi:10.1371/journal.pone.0286654)
Supplement: S1 Data — (DOCX) [file pone.0286654.s001.docx]

| ID | CRP | Adenomatous | Gender | Age | BMI | Waist | FPG | Chol | HDL | LDL | TG | UA | 25(OH)D | SBP | DBP | HbA1c | Abdominal_obesity | Higher_TG | Lower_HDL | Elevated_BP | Hyperglycemia | MetS |
| --- | --- | --- | --- | --- | --- | --- | --- | --- | --- | --- | --- | --- | --- | --- | --- | --- | --- | --- | --- | --- | --- | --- |
| 1 | 1 | 0 | 0 | 59 | 29.9 | 102.0 | 105 | 162 | 39 | 73 | 310 | 5.5 | 23.3 | 136 | 77 | 5.9 | 1 | 1 | 1 | 1 | 1 | 1 |
| 2 | 0 |  | 1 | 43 | 25.9 | 82.0 | 99 | 188 | 58 | 111 | 140 | 5.9 | 19.8 | 152 | 91 | 5.5 | 0 | 0 | 0 | 1 | 0 | 0 |
| 3 | 1 | 1 | 1 | 60 | 23.9 | 96.0 | 94 | 227 | 47 | 153 | 121 | 7.4 | 27.6 | 100 | 52 | 5.3 | 1 | 0 | 0 | 0 | 0 | 0 |
| 4 | 0 |  | 1 | 63 | 27.5 | 88.0 | 94 | 118 | 41 | 59 | 90 | 5.7 | 34.4 | 128 | 78 | 5.1 | 0 | 0 | 0 | 1 | 0 | 0 |
| 5 | 1 | 1 | 1 | 36 | 24.3 | 83.0 | 87 | 171 | 31 | 114 | 126 | 7.9 | 32.0 | 113 | 78 | 5.2 | 0 | 0 | 1 | 0 | 0 | 0 |
| 6 | 0 |  | 0 | 61 | 22.9 | 83.0 | 105 | 185 | 48 | 127 | 47 | 4.3 | 24.6 | 120 | 67 | 6.4 | 1 | 0 | 1 | 0 | 1 | 1 |
| 7 | 0 |  | 0 | 57 | 21.8 | 73.0 | 94 | 258 | 57 | 178 | 75 | 4.6 | 27.6 | 98 | 61 | 5.7 | 0 | 0 | 0 | 1 | 0 | 0 |
| 8 | 0 |  | 1 | 35 | 24.3 | 76.5 | 92 | 199 | 33 | 143 | 116 | 6.3 | 23.2 | 96 | 70 | 5.6 | 0 | 0 | 1 | 1 | 0 | 0 |
| 9 | 0 |  | 1 | 61 | 27.2 | 94.0 | 104 | 155 | 44 | 94 | 90 | 4.4 | 37.6 | 124 | 74 | 5.8 | 1 | 0 | 0 | 1 | 1 | 1 |
| 10 | 1 | 0 | 1 | 34 | 21.4 | 79.0 | 89 | 149 | 47 | 91 | 61 | 6.7 | 15.1 | 128 | 78 | 5.1 | 0 | 0 | 0 | 0 | 0 | 0 |
| 11 | 0 |  | 1 | 33 | 26.0 | 87.0 | 77 | 217 | 42 | 142 | 138 | 6.1 | 22.5 | 116 | 66 | 4.8 | 0 | 0 | 0 | 0 | 0 | 0 |
| 12 | 0 |  | 1 | 50 | 29.8 | 93.0 | 108 | 150 | 35 | 106 | 71 | 7.1 | 39.9 | 124 | 89 | 6.3 | 1 | 0 | 1 | 1 | 1 | 1 |
| 13 | 0 |  | 0 | 59 | 21.4 | 71.0 | 93 | 253 | 45 | 206 | 70 | 5.1 | 17.4 | 108 | 69 | 5.5 | 0 | 0 | 1 | 0 | 0 | 0 |
| 14 | 0 |  | 1 | 33 | 23.9 | 77.0 | 80 | 204 | 67 | 126 | 68 | 6.9 | 31.3 | 112 | 64 | 5.0 | 0 | 0 | 0 | 0 | 0 | 0 |
| 15 | 1 | 1 | 0 | 61 | 22.1 | 80.5 | 127 | 299 | 57 | 221 | 103 | 5.4 | 29.2 | 115 | 72 | 6.6 | 1 | 0 | 0 | 0 | 1 | 0 |
| 16 | 1 | 1 | 0 | 62 | 23.9 | 71.0 | 87 | 197 | 67 | 61 | 171 | 4.8 | 34.5 | 126 | 79 | 5.6 | 0 | 1 | 0 | 0 | 0 | 0 |
| 17 | 0 |  | 1 | 31 | 30.1 | 83.0 | 85 | 228 | 43 | 179 | 90 | 8.8 | 24.2 | 128 | 75 | 5.2 | 0 | 0 | 0 | 0 | 0 | 0 |
| 18 | 0 |  | 1 | 53 | 23.9 | 78.0 | 99 | 211 | 53 | 153 | 99 | 4.7 | 24.6 | 115 | 72 | 5.6 | 0 | 0 | 0 | 0 | 0 | 0 |
| 19 | 0 |  | 1 | 62 | 23.1 | 84.0 | 113 | 192 | 40 | 75 | 407 | 5.6 | 20.5 | 126 | 79 | 5.3 | 0 | 1 | 0 | 1 | 1 | 1 |
| 20 | 0 |  | 1 | 55 | 17.6 | 63.0 | 79 | 197 | 59 | 126 | 81 | 5.4 | 28.3 | 133 | 82 | 5.1 | 0 | 0 | 0 | 1 | 0 | 0 |
| 21 | 0 |  | 1 | 55 | 23.3 | 83.0 | 112 | 155 | 36 | 89 | 171 | 6.8 | 28.9 | 110 | 77 | 6.8 | 0 | 1 | 1 | 1 | 1 | 1 |
| 22 | 0 |  | 1 | 35 | 25.0 | 78.0 | 83 | 213 | 67 | 121 | 179 | 8.3 | 30.7 | 121 | 80 | 5.3 | 0 | 1 | 0 | 0 | 0 | 0 |
| 23 | 1 | 0 | 0 | 52 | 33.2 | 87.0 | 103 | 218 | 52 | 148 | 75 | 4.7 | 26.7 | 131 | 80 | 4.4 | 1 | 0 | 0 | 1 | 1 | 1 |
| 24 | 1 | 1 | 1 | 32 | 29.3 | 95.5 | 91 | 228 | 42 | 161 | 112 | 6.6 | 56.8 | 117 | 65 | 5.5 | 1 | 0 | 0 | 0 | 0 | 0 |
| 25 | 1 | 0 | 1 | 58 | 24.0 | 76.0 | 79 | 242 | 62 | 147 | 112 | 5.8 | 45.3 | 133 | 79 | 4.2 | 0 | 0 | 0 | 1 | 0 | 0 |
| 26 | 1 | 0 | 1 | 54 | 26.9 | 93.0 | 102 | 173 | 41 | 107 | 152 | 6.0 | 26.0 | 132 | 93 | 5.8 | 1 | 1 | 0 | 1 | 1 | 1 |
| 27 | 0 |  | 0 | 56 | 20.9 | 74.0 | 90 | 219 | 69 | 138 | 80 | 4.1 | 42.4 | 117 | 50 | 5.5 | 0 | 0 | 0 | 0 | 0 | 0 |
| 28 | 0 |  | 0 | 54 | 25.7 | 78.5 | 90 | 207 | 64 | 132 | 58 | 5.0 | 21.4 | 105 | 66 | 5.0 | 0 | 0 | 0 | 0 | 0 | 0 |
| 29 | 1 | 1 | 1 | 47 | 23.1 | 89.5 | 90 | 208 | 42 | 135 | 185 | 7.7 | 28.9 | 120 | 82 | 5.5 | 0 | 1 | 0 | 0 | 0 | 0 |
| 30 | 0 |  | 1 | 43 | 27.7 | 92.0 | 98 | 155 | 30 | 93 | 151 | 6.2 | 23.6 | 125 | 76 | 5.5 | 1 | 1 | 1 | 0 | 0 | 1 |
| 31 | 0 |  | 0 | 59 | 24.6 | 75.0 | 92 | 241 | 49 | 164 | 91 | 6.9 | 39.9 | 127 | 76 | 5.4 | 0 | 0 | 1 | 0 | 0 | 0 |
| 32 | 1 | 1 | 1 | 42 | 25.0 | 84.0 | 111 | 150 | 31 | 83 | 132 | 3.2 | 32.3 | 123 | 73 | 4.7 | 0 | 0 | 1 | 0 | 1 | 0 |
| 33 | 0 |  | 1 | 40 | 29.1 | 89.0 | 95 | 224 | 42 | 164 | 61 | 7.7 | 29.6 | 127 | 80 | 6.1 | 0 | 0 | 0 | 0 | 1 | 0 |
| 34 | 0 |  | 0 | 61 | 26.9 | 82.0 | 85 | 285 | 46 | 206 | 226 | 6.0 | 25.5 | 116 | 63 | 5.8 | 1 | 1 | 1 | 0 | 1 | 1 |
| 35 | 0 |  | 1 | 38 | 23.8 | 79.0 | 93 | 214 | 52 | 140 | 84 | 6.2 | 28.4 | 145 | 80 | 5.1 | 0 | 0 | 0 | 1 | 0 | 0 |
| 36 | 1 | 0 | 1 | 37 | 30.8 | 102.0 | 96 | 179 | 30 | 122 | 300 | 6.2 | 20.8 | 110 | 76 | 5.7 | 1 | 1 | 1 | 0 | 0 | 1 |
| 37 | 0 |  | 0 | 34 | 20.0 | 70.0 | 85 | 239 | 101 | 108 | 61 | 3.8 | 17.4 | 103 | 67 | 5.1 | 0 | 0 | 0 | 0 | 0 | 0 |
| 38 | 0 |  | 1 | 54 | 26.1 | 81.0 | 93 | 277 | 31 | 157 | 409 | 12.5 | 26.2 | 132 | 85 | 5.5 | 0 | 1 | 1 | 1 | 0 | 1 |
| 39 | 0 |  | 1 | 43 | 22.4 | 78.0 | 83 | 284 | 73 | 176 | 82 | 7.7 | 24.7 | 118 | 75 | 4.0 | 0 | 0 | 0 | 0 | 0 | 0 |
| 40 | 1 | 1 | 0 | 55 | 24.3 | 85.0 | 84 | 267 | 55 | 177 | 73 | 5.6 | 25.3 | 108 | 71 | 5.5 | 1 | 0 | 0 | 0 | 0 | 0 |
| 41 | 0 |  | 0 | 54 | 22.9 | 84.0 | 88 | 199 | 51 | 134 | 102 | 5.3 | 50.3 | 118 | 74 | 5.4 | 1 | 0 | 0 | 0 | 0 | 0 |
| 42 | 0 |  | 0 | 54 | 24.0 | 76.0 | 89 | 210 | 63 | 134 | 43 | 3.8 | 24.4 | 128 | 72 | 5.6 | 0 | 0 | 0 | 0 | 0 | 0 |
| 43 | 0 |  | 1 | 50 | 28.4 | 95.5 | 110 | 185 | 60 | 92 | 174 | 7.9 | 57.8 | 132 | 85 | 4.1 | 1 | 1 | 0 | 1 | 1 | 1 |
| 44 | 1 | 1 | 0 | 45 | 24.2 | 78.0 | 92 | 149 | 56 | 79 | 45 | 4.1 | 14.7 | 112 | 79 | 5.2 | 0 | 0 | 0 | 0 | 0 | 0 |
| 45 | 1 | 0 | 1 | 61 | 22.3 | 83.0 | 108 | 200 | 43 | 140 | 144 | 8.6 | 44.0 | 146 | 102 | 5.4 | 0 | 0 | 0 | 1 | 1 | 0 |
| 46 | 1 | 1 | 1 | 42 | 22.9 | 81.5 | 83 | 224 | 72 | 141 | 44 | 5.1 | 31.0 | 138 | 89 | 5.2 | 0 | 0 | 0 | 1 | 0 | 0 |
| 47 | 1 | 1 | 1 | 41 | 24.7 | 86.0 | 99 | 180 | 35 | 109 | 205 | 7.1 | 25.2 | 120 | 78 | 5.2 | 0 | 1 | 1 | 0 | 0 | 0 |
| 48 | 0 |  | 1 | 40 | 25.0 | 78.0 | 91 | 189 | 72 | 96 | 122 | 4.3 | 30.3 | 101 | 62 | 3.7 | 0 | 0 | 0 | 0 | 0 | 0 |
| 49 | 1 | 1 | 1 | 53 | 22.7 | 85.0 | 94 | 243 | 45 | 167 | 105 | 6.1 | 27.7 | 131 | 86 | 5.1 | 0 | 0 | 0 | 1 | 0 | 0 |
| 50 | 0 |  | 0 | 45 | 27.5 | 85.0 | 101 | 236 | 47 | 176 | 106 | 4.1 | 16.2 | 117 | 70 | 5.6 | 1 | 0 | 1 | 0 | 1 | 1 |
| 51 | 1 | 1 | 0 | 55 | 27.1 | 78.5 | 92 | 281 | 50 | 164 | 321 | 6.4 | 20.2 | 100 | 68 | 5.4 | 0 | 1 | 0 | 0 | 0 | 0 |
| 52 | 1 | 1 | 1 | 60 | 25.0 | 88.0 | 118 | 147 | 67 | 63 | 63 | 4.8 | 41.8 | 111 | 73 | 5.7 | 0 | 0 | 0 | 1 | 1 | 0 |
| 53 | 0 |  | 1 | 46 | 27.6 | 88.5 | 90 | 192 | 52 | 126 | 106 | 6.6 | 33.3 | 106 | 72 | 5.2 | 0 | 0 | 0 | 0 | 0 | 0 |
| 54 | 1 | 1 | 0 | 60 | 20.4 | 78.5 | 90 | 266 | 64 | 177 | 58 | 5.3 | 22.6 | 127 | 79 | 5.9 | 0 | 0 | 0 | 0 | 1 | 0 |
| 55 | 1 | 1 | 0 | 59 | 21.1 | 67.5 | 98 | 171 | 65 | 83 | 116 | 3.8 | 62.4 | 93 | 57 | 5.1 | 0 | 0 | 0 | 1 | 0 | 0 |
| 56 | 1 | 1 | 1 | 59 | 30.4 | 108.0 | 159 | 183 | 47 | 118 | 160 | 6.3 | 22.4 | 129 | 86 | 7.3 | 1 | 1 | 0 | 1 | 1 | 1 |
| 57 | 1 | 1 | 0 | 55 | 24.9 | 76.0 | 89 | 199 | 50 | 134 | 80 | 6.0 | 31.4 | 123 | 68 | 4.8 | 0 | 0 | 0 | 0 | 0 | 0 |
| 58 | 1 | 1 | 0 | 52 | 25.6 | 87.0 | 93 | 227 | 63 | 140 | 80 | 4.3 | 26.2 | 116 | 69 | 5.1 | 1 | 0 | 0 | 0 | 0 | 0 |
| 59 | 1 | 0 | 1 | 46 | 23.3 | 86.5 | 99 | 155 | 39 | 98 | 65 | 6.0 | 48.6 | 119 | 77 | 6.1 | 0 | 0 | 1 | 0 | 1 | 0 |
| 60 | 0 |  | 0 | 44 | 18.0 | 65.5 | 88 | 192 | 51 | 130 | 82 | 3.9 | 11.5 | 91 | 59 | 5.5 | 0 | 0 | 0 | 1 | 0 | 0 |
| 61 | 1 | 0 | 1 | 59 | 21.8 | 78.0 | 91 | 166 | 45 | 103 | 197 | 6.2 | 37.7 | 107 | 66 | 5.6 | 0 | 1 | 0 | 0 | 0 | 0 |
| 62 | 1 | 1 | 1 | 51 | 28.5 | 94.5 | 91 | 139 | 35 | 81 | 139 | 7.9 | 17.5 | 113 | 82 | 5.1 | 1 | 0 | 1 | 0 | 0 | 0 |
| 63 | 0 |  | 1 | 60 | 23.9 | 86.5 | 82 | 170 | 47 | 109 | 49 | 5.4 | 45.6 | 126 | 88 | 5.3 | 0 | 0 | 0 | 1 | 0 | 0 |
| 64 | 1 | 1 | 1 | 38 | 24.6 | 84.0 | 90 | 240 | 50 | 170 | 156 | 6.6 | 20.3 | 106 | 70 | 5.5 | 0 | 1 | 0 | 0 | 0 | 0 |
| 65 | 0 |  | 0 | 37 | 21.4 | 71.0 | 78 | 200 | 59 | 128 | 77 | 4.7 | 27.4 | 109 | 63 | 4.9 | 0 | 0 | 0 | 0 | 0 | 0 |
| 66 | 1 | 1 | 1 | 36 | 27.6 | 90.0 | 86 | 257 | 37 | 155 | 413 | 8.0 | 29.9 | 133 | 84 | 5.3 | 1 | 1 | 1 | 1 | 0 | 1 |
| 67 | 0 |  | 0 | 63 | 21.3 | 84.5 | 285 | 238 | 58 | 158 | 204 | 6.1 | 17.1 | 128 | 67 | 10.0 | 1 | 1 | 0 | 0 | 1 | 1 |
| 68 | 1 | 1 | 0 | 58 | 21.6 | 78.0 | 75 | 251 | 60 | 176 | 54 | 5.2 | 19.5 | 124 | 76 | 5.0 | 0 | 0 | 0 | 0 | 0 | 0 |
| 69 | 0 |  | 0 | 34 | 20.0 | 67.0 | 93 | 200 | 81 | 107 | 47 | 4.0 | 26.3 | 109 | 66 | 5.0 | 0 | 0 | 0 | 0 | 0 | 0 |
| 70 | 0 |  | 0 | 58 | 22.9 | 77.0 | 76 | 174 | 60 | 98 | 71 | 4.7 | 32.1 | 143 | 84 | 5.3 | 0 | 0 | 0 | 1 | 0 | 0 |
| 71 | 0 |  | 0 | 60 | 23.6 | 70.0 | 93 | 231 | 58 | 165 | 98 | 5.8 | 30.0 | 107 | 68 | 5.7 | 0 | 0 | 0 | 0 | 0 | 0 |
| 72 | 0 |  | 1 | 38 | 22.5 | 79.0 | 98 | 246 | 54 | 165 | 96 | 5.9 | 49.6 | 111 | 72 | 5.3 | 0 | 0 | 0 | 0 | 0 | 0 |
| 73 | 0 |  | 1 | 61 | 26.9 | 93.0 | 94 | 223 | 40 | 152 | 132 | 5.1 | 20.2 | 127 | 73 | 5.4 | 1 | 0 | 0 | 0 | 0 | 0 |
| 74 | 1 | 0 | 1 | 64 | 23.9 | 84.5 | 105 | 212 | 67 | 128 | 60 | 7.1 | 68.8 | 127 | 89 | 6.3 | 0 | 0 | 0 | 1 | 1 | 0 |
| 75 | 0 |  | 0 | 56 | 21.0 | 72.5 | 94 | 243 | 66 | 160 | 54 | 4.6 | 35.6 | 106 | 68 | 5.3 | 0 | 0 | 0 | 0 | 0 | 0 |
| 76 | 0 |  | 0 | 50 | 22.3 | 69.0 | 101 | 209 | 57 | 134 | 84 | 5.9 | 32.9 | 97 | 68 | 5.5 | 0 | 0 | 0 | 1 | 1 | 0 |
| 77 | 0 |  | 1 | 53 | 23.9 | 79.0 | 93 | 154 | 46 | 93 | 47 | 7.8 | 43.5 | 133 | 83 | 5.7 | 0 | 0 | 0 | 1 | 0 | 0 |
| 78 | 0 |  | 0 | 62 | 21.1 | 73.0 | 93 | 194 | 45 | 131 | 66 | 3.4 | 30.1 | 111 | 74 | 5.4 | 0 | 0 | 1 | 0 | 0 | 0 |
| 79 | 0 |  | 0 | 62 | 25.8 | 79.5 | 110 | 221 | 42 | 140 | 154 | 5.1 | 13.2 | 142 | 75 | 5.7 | 0 | 1 | 1 | 1 | 1 | 1 |
| 80 | 1 | 0 | 1 | 60 | 23.8 | 89.0 | 99 | 206 | 65 | 131 | 68 | 6.2 | 38.4 | 127 | 83 | 5.9 | 0 | 0 | 0 | 0 | 1 | 0 |
| 81 | 1 | 0 | 1 | 62 | 22.4 | 78.0 | 154 | 185 | 43 | 130 | 63 | 7.3 | 32.6 | 136 | 72 | 8.4 | 0 | 0 | 0 | 1 | 1 | 0 |
| 82 | 1 | 1 | 0 | 63 | 33.6 | 107.0 | 136 | 211 | 32 | 152 | 142 | 9.0 | 22.6 | 148 | 77 | 6.8 | 1 | 0 | 1 | 1 | 1 | 1 |
| 83 | 0 |  | 0 | 35 | 22.3 | 70.0 | 85 | 141 | 39 | 88 | 80 | 4.4 | 22.0 | 120 | 78 | 5.0 | 0 | 0 | 1 | 0 | 0 | 0 |
| 84 | 0 |  | 1 | 35 | 24.4 | 86.0 | 89 | 226 | 37 | 137 | 326 | 5.8 | 12.1 | 127 | 79 | 5.3 | 0 | 1 | 1 | 0 | 0 | 0 |
| 85 | 0 |  | 1 | 63 | 23.4 | 79.0 | 98 | 150 | 32 | 60 | 316 | 6.1 | 29.6 | 136 | 83 | 3.8 | 0 | 1 | 1 | 1 | 0 | 1 |
| 86 | 0 |  | 0 | 58 | 19.0 | 60.0 | 102 | 239 | 64 | 150 | 63 | 3.8 | 32.0 | 121 | 75 | 5.3 | 0 | 0 | 0 | 0 | 1 | 0 |
| 87 | 0 |  | 0 | 58 | 22.1 | 75.0 | 89 | 183 | 47 | 128 | 48 | 3.7 | 27.6 | 120 | 78 | 7.4 | 0 | 0 | 1 | 0 | 1 | 0 |
| 88 | 0 |  | 0 | 53 | 28.3 | 86.0 | 103 | 252 | 74 | 167 | 101 | 6.1 | 25.5 | 112 | 75 | 5.7 | 1 | 0 | 0 | 0 | 1 | 0 |
| 89 | 0 |  | 0 | 57 | 24.5 | 80.0 | 96 | 229 | 70 | 143 | 47 | 5.0 | 22.0 | 131 | 84 | 5.4 | 1 | 0 | 0 | 1 | 0 | 0 |
| 90 | 0 |  | 0 | 59 | 19.8 | 66.0 | 96 | 193 | 45 | 124 | 81 | 5.2 | 31.5 | 119 | 67 | 5.4 | 0 | 0 | 1 | 0 | 0 | 0 |
| 91 | 0 |  | 0 | 58 | 28.7 | 80.0 | 95 | 182 | 45 | 115 | 99 | 6.2 | 26.3 | 132 | 84 | 5.6 | 1 | 0 | 1 | 1 | 0 | 1 |
| 92 | 1 | 0 | 1 | 59 | 23.8 | 80.5 | 117 | 169 | 33 | 115 | 135 | 6.7 | 38.3 | 132 | 96 | 6.2 | 0 | 0 | 1 | 1 | 1 | 1 |
| 93 | 0 |  | 0 | 55 | 26.3 | 80.0 | 105 | 289 | 62 | 208 | 97 | 4.6 | 29.1 | 112 | 74 | 5.6 | 1 | 0 | 0 | 0 | 1 | 0 |
| 94 | 0 |  | 0 | 54 | 23.5 | 82.0 | 90 | 236 | 61 | 162 | 108 | 5.4 | 33.3 | 115 | 76 | 4.6 | 1 | 0 | 0 | 0 | 0 | 0 |
| 95 | 0 |  | 1 | 62 | 28.8 | 97.0 | 125 | 187 | 25 | 133 | 137 | 7.5 | 38.7 | 130 | 84 | 6.3 | 1 | 0 | 1 | 1 | 1 | 1 |
| 96 | 0 |  | 1 | 33 | 27.3 | 93.0 | 89 | 158 | 74 | 70 | 44 | 6.8 | 51.4 | 120 | 72 | 5.6 | 1 | 0 | 0 | 0 | 0 | 0 |
| 97 | 1 | 0 | 1 | 62 | 22.1 | 73.0 | 92 | 151 | 55 | 77 | 88 | 6.7 | 19.4 | 132 | 83 | 5.5 | 0 | 0 | 0 | 1 | 0 | 0 |
| 98 | 0 |  | 1 | 63 | 27.8 | 88.0 | 93 | 200 | 39 | 140 | 61 | 6.3 | 45.1 | 133 | 85 | 5.9 | 0 | 0 | 1 | 1 | 1 | 1 |
| 99 | 1 | 0 | 0 | 51 | 24.3 | 75.0 | 93 | 240 | 53 | 162 | 122 | 4.9 | 20.5 | 118 | 78 | 5.3 | 0 | 0 | 0 | 0 | 0 | 0 |
| 100 | 1 | 0 | 0 | 44 | 25.3 | 79.0 | 92 | 277 | 78 | 183 | 51 | 4.4 | 16.9 | 106 | 72 | 5.3 | 0 | 0 | 0 | 0 | 0 | 0 |
| 101 | 1 | 1 | 1 | 42 | 22.6 | 79.0 | 93 | 167 | 40 | 99 | 152 | 4.7 | 21.2 | 112 | 73 | 5.6 | 0 | 1 | 0 | 0 | 0 | 0 |
| 102 | 1 | 0 | 0 | 41 | 34.4 | 97.0 | 88 | 197 | 35 | 122 | 220 | 6.1 | 24.7 | 132 | 72 | 4.8 | 1 | 1 | 1 | 1 | 0 | 1 |
| 103 | 0 |  | 0 | 45 | 19.2 | 68.5 | 90 | 205 | 61 | 120 | 49 | 4.6 | 16.9 | 87 | 52 | 5.4 | 0 | 0 | 0 | 1 | 0 | 0 |
| 104 | 1 | 1 | 1 | 61 | 26.3 | 83.0 | 88 | 135 | 43 | 72 | 99 | 6.9 | 27.8 | 117 | 69 | 5.7 | 0 | 0 | 0 | 1 | 0 | 0 |
| 105 | 0 |  | 0 | 56 | 22.7 | 73.0 | 85 | 245 | 40 | 177 | 121 | 3.9 | 26.7 | 134 | 81 | 4.9 | 0 | 0 | 1 | 1 | 0 | 0 |
| 106 | 0 |  | 0 | 64 | 22.7 | 73.0 | 86 | 191 | 71 | 103 | 30 | 3.4 | 29.0 | 109 | 62 | 5.3 | 0 | 0 | 0 | 0 | 0 | 0 |
| 107 | 0 |  | 0 | 32 | 18.9 | 62.0 | 89 | 209 | 84 | 108 | 31 | 5.3 | 20.1 | 97 | 59 | 5.3 | 0 | 0 | 0 | 1 | 0 | 0 |
| 108 | 0 |  | 0 | 62 | 21.7 | 79.0 | 88 | 175 | 60 | 107 | 38 | 4.9 | 24.6 | 111 | 73 | 5.4 | 0 | 0 | 0 | 0 | 0 | 0 |
| 109 | 1 | 1 | 0 | 52 | 24.4 | 72.0 | 87 | 231 | 48 | 166 | 115 | 4.2 | 17.9 | 112 | 65 | 5.6 | 0 | 0 | 1 | 0 | 0 | 0 |
| 110 | 1 | 1 | 0 | 56 | 26.5 | 90.0 | 127 | 167 | 56 | 96 | 75 | 5.1 | 68.5 | 165 | 100 | 5.9 | 1 | 0 | 0 | 1 | 1 | 1 |
| 111 | 0 |  | 0 | 54 | 22.8 | 73.0 | 91 | 261 | 60 | 175 | 84 | 4.1 | 28.6 | 111 | 63 | 5.8 | 0 | 0 | 0 | 0 | 1 | 0 |
| 112 | 0 |  | 1 | 45 | 28.4 | 85.5 | 103 | 170 | 39 | 116 | 70 | 7.0 | 26.8 | 124 | 88 | 5.6 | 0 | 0 | 1 | 1 | 1 | 1 |
| 113 | 1 | 1 | 1 | 59 | 22.9 | 75.0 | 80 | 241 | 49 | 166 | 42 | 7.2 | 47.0 | 118 | 74 | 5.0 | 0 | 0 | 0 | 0 | 0 | 0 |
| 114 | 1 | 1 | 1 | 51 | 25.3 | 85.0 | 97 | 186 | 36 | 135 | 142 | 7.4 | 22.6 | 107 | 70 | 5.4 | 0 | 0 | 1 | 0 | 0 | 0 |
| 115 | 0 |  | 1 | 55 | 28.9 | 94.0 | 136 | 131 | 36 | 84 | 69 | 5.9 | 29.0 | 133 | 84 | 7.7 | 1 | 0 | 1 | 1 | 1 | 1 |
| 116 | 0 |  | 0 | 63 | 21.4 | 73.0 | 97 | 241 | 64 | 158 | 73 | 4.8 | 41.9 | 98 | 57 | 5.9 | 0 | 0 | 0 | 1 | 1 | 0 |
| 117 | 1 | 0 | 1 | 49 | 23.7 | 80.0 | 91 | 191 | 45 | 121 | 110 | 6.5 | 40.1 | 120 | 81 | 5.2 | 0 | 0 | 0 | 0 | 0 | 0 |
| 118 | 1 | 0 | 1 | 59 | 30.7 | 104.0 | 108 | 212 | 57 | 125 | 161 | 6.7 | 30.5 | 107 | 67 | 6.6 | 1 | 1 | 0 | 0 | 1 | 1 |
| 119 | 0 |  | 1 | 36 | 24.5 | 85.0 | 85 | 234 | 52 | 157 | 87 | 5.0 | 33.5 | 132 | 85 | 5.3 | 0 | 0 | 0 | 1 | 0 | 0 |
| 120 | 0 |  | 1 | 35 | 28.0 | 95.0 | 96 | 271 | 53 | 195 | 76 | 7.4 | 18.7 | 136 | 85 | 5.6 | 1 | 0 | 0 | 1 | 0 | 0 |
| 121 | 1 | 0 | 1 | 42 | 27.7 | 88.0 | 95 | 269 | 54 | 172 | 240 | 6.0 | 39.5 | 128 | 84 | 5.1 | 0 | 1 | 0 | 0 | 0 | 0 |
| 122 | 0 |  | 0 | 56 | 24.5 | 77.0 | 89 | 275 | 83 | 168 | 53 | 3.1 | 31.7 | 118 | 70 | 5.8 | 0 | 0 | 0 | 1 | 1 | 0 |
| 123 | 1 | 0 | 0 | 48 | 21.7 | 72.0 | 91 | 223 | 53 | 140 | 135 | 5.4 | 21.6 | 119 | 77 | 5.6 | 0 | 0 | 0 | 0 | 0 | 0 |
| 124 | 0 |  | 0 | 51 | 25.6 | 78.0 | 93 | 249 | 48 | 180 | 175 | 5.7 | 14.6 | 109 | 75 | 5.5 | 0 | 1 | 1 | 0 | 0 | 0 |
| 125 | 1 | 0 | 1 | 41 | 30.2 | 92.0 | 89 | 154 | 33 | 104 | 106 | 4.7 | 27.2 | 121 | 79 | 5.1 | 1 | 0 | 1 | 0 | 0 | 0 |
| 126 | 1 | 1 | 1 | 49 | 24.9 | 87.0 | 89 | 200 | 39 | 141 | 104 | 6.4 | 29.4 | 117 | 80 | 5.4 | 0 | 0 | 1 | 0 | 0 | 0 |
| 127 | 1 | 0 | 0 | 55 | 30.5 | 96.0 | 95 | 195 | 37 | 148 | 166 | 5.7 | 19.9 | 134 | 67 | 5.0 | 1 | 1 | 1 | 1 | 0 | 1 |
| 128 | 1 | 1 | 1 | 54 | 19.5 | 70.0 | 75 | 249 | 72 | 169 | 37 | 6.2 | 33.0 | 116 | 75 | 5.1 | 0 | 0 | 0 | 0 | 0 | 0 |
| 129 | 1 | 0 | 1 | 45 | 26.3 | 88.0 | 94 | 233 | 45 | 159 | 157 | 6.7 | 25.0 | 133 | 79 | 5.1 | 0 | 1 | 0 | 1 | 0 | 0 |
| 130 | 1 | 0 | 1 | 41 | 29.2 | 99.0 | 98 | 227 | 46 | 154 | 101 | 6.4 | 20.0 | 112 | 88 | 5.5 | 1 | 0 | 0 | 1 | 0 | 0 |
| 131 | 0 |  | 1 | 41 | 21.4 | 75.0 | 79 | 144 | 49 | 86 | 49 | 5.8 | 24.2 | 118 | 75 | 4.9 | 0 | 0 | 0 | 0 | 0 | 0 |
| 132 | 0 |  | 0 | 40 | 22.0 | 78.0 | 81 | 248 | 79 | 145 | 81 | 4.7 | 20.2 | 104 | 64 | 5.2 | 0 | 0 | 0 | 0 | 0 | 0 |
| 133 | 1 | 1 | 0 | 60 | 23.0 | 81.0 | 119 | 221 | 35 | 128 | 232 | 5.5 | 23.9 | 137 | 65 | 6.0 | 1 | 1 | 1 | 1 | 1 | 1 |
| 134 | 0 |  | 0 | 37 | 21.1 | 70.0 | 78 | 178 | 72 | 89 | 83 | 4.1 | 23.0 | 100 | 57 | 5.4 | 0 | 0 | 0 | 0 | 0 | 0 |
| 135 | 1 | 0 | 1 | 36 | 24.0 | 81.0 | 99 | 202 | 37 | 140 | 138 | 6.5 | 23.4 | 118 | 82 | 5.8 | 0 | 0 | 1 | 0 | 1 | 0 |
| 136 | 1 | 0 | 0 | 57 | 22.9 | 72.0 | 92 | 256 | 93 | 130 | 65 | 4.5 | 34.8 | 145 | 76 | 5.6 | 0 | 0 | 0 | 1 | 0 | 0 |
| 137 | 0 |  | 1 | 33 | 26.8 | 88.0 | 100 | 184 | 26 | 118 | 195 | 8.1 | 33.5 | 133 | 82 | 5.3 | 0 | 1 | 1 | 1 | 1 | 1 |
| 138 | 1 | 1 | 1 | 55 | 24.5 | 84.0 | 88 | 147 | 62 | 74 | 48 | 7.8 | 48.2 | 132 | 78 | 5.6 | 0 | 0 | 0 | 1 | 0 | 0 |
| 139 | 0 |  | 1 | 52 | 22.9 | 79.5 | 99 | 205 | 34 | 150 | 80 | 7.5 | 23.4 | 128 | 78 | 4.2 | 0 | 0 | 1 | 0 | 0 | 0 |
| 140 | 1 | 0 | 0 | 58 | 24.4 | 79.0 | 90 | 194 | 52 | 132 | 59 | 4.8 | 25.1 | 101 | 63 | 5.7 | 0 | 0 | 0 | 0 | 0 | 0 |
| 141 | 1 | 1 | 1 | 53 | 22.1 | 77.0 | 94 | 198 | 69 | 111 | 62 | 6.5 | 26.4 | 97 | 63 | 4.5 | 0 | 0 | 0 | 1 | 0 | 0 |
| 142 | 0 |  | 0 | 48 | 18.1 | 68.0 | 92 | 284 | 110 | 149 | 34 | 4.3 | 26.5 | 106 | 67 | 5.4 | 0 | 0 | 0 | 0 | 0 | 0 |
| 143 | 0 |  | 1 | 64 | 24.2 | 80.0 | 90 | 157 | 41 | 105 | 69 | 5.4 | 36.2 | 184 | 97 | 4.9 | 0 | 0 | 0 | 1 | 0 | 0 |
| 144 | 1 | 0 | 0 | 54 | 24.5 | 75.5 | 87 | 242 | 84 | 136 | 106 | 5.4 | 22.4 | 148 | 91 | 5.3 | 0 | 0 | 0 | 1 | 0 | 0 |
| 145 | 0 |  | 0 | 46 | 22.0 | 78.0 | 81 | 204 | 78 | 112 | 43 | 5.5 | 28.3 | 114 | 77 | 4.9 | 0 | 0 | 0 | 0 | 0 | 0 |
| 146 | 0 |  | 0 | 49 | 21.9 | 75.0 | 87 | 208 | 65 | 112 | 70 | 7.2 | 25.1 | 108 | 79 | 5.0 | 0 | 0 | 0 | 0 | 0 | 0 |
| 147 | 1 | 0 | 1 | 45 | 26.7 | 92.0 | 90 | 145 | 35 | 85 | 127 | 6.2 | 19.4 | 117 | 71 | 5.2 | 1 | 0 | 1 | 1 | 0 | 1 |
| 148 | 0 |  | 0 | 64 | 22.2 | 78.0 | 95 | 252 | 53 | 172 | 108 | 4.5 | 25.6 | 136 | 75 | 5.3 | 0 | 0 | 0 | 1 | 0 | 0 |
| 149 | 0 |  | 0 | 41 | 21.5 | 73.0 | 78 | 165 | 46 | 108 | 56 | 3.8 | 20.8 | 100 | 55 | 5.1 | 0 | 0 | 1 | 0 | 0 | 0 |
| 150 | 1 | 1 | 0 | 64 | 30.6 | 98.0 | 84 | 164 | 41 | 111 | 196 | 4.0 | 23.9 | 109 | 71 | 5.5 | 1 | 1 | 1 | 0 | 0 | 1 |
| 151 | 1 | 1 | 1 | 63 | 19.5 | 69.5 | 112 | 165 | 38 | 115 | 128 | 5.3 | 28.6 | 130 | 76 | 5.7 | 0 | 0 | 1 | 1 | 1 | 1 |
| 152 | 0 |  | 1 | 63 | 26.5 | 90.0 | 83 | 205 | 44 | 173 | 71 | 6.3 | 30.2 | 122 | 77 | 5.4 | 1 | 0 | 0 | 0 | 0 | 0 |
| 153 | 1 | 0 | 0 | 50 | 30.9 | 90.0 | 102 | 183 | 52 | 115 | 88 | 4.6 | 11.5 | 103 | 71 | 5.3 | 1 | 0 | 0 | 1 | 1 | 1 |
| 154 | 0 |  | 0 | 35 | 21.9 | 64.0 | 84 | 181 | 69 | 96 | 69 | 4.5 | 24.5 | 96 | 64 | 5.3 | 0 | 0 | 0 | 1 | 0 | 0 |
| 155 | 0 |  | 0 | 35 | 19.5 | 67.0 | 82 | 155 | 39 | 97 | 107 | 4.8 | 30.8 | 102 | 61 | 5.0 | 0 | 0 | 1 | 0 | 0 | 0 |
| 156 | 1 | 1 | 0 | 61 | 20.4 | 66.5 | 102 | 277 | 74 | 176 | 67 | 4.4 | 26.8 | 132 | 76 | 5.3 | 0 | 0 | 0 | 1 | 1 | 0 |
| 157 | 1 | 0 | 0 | 43 | 18.7 | 67.0 | 82 | 187 | 52 | 117 | 53 | 4.3 | 16.3 | 115 | 60 | 5.2 | 0 | 0 | 0 | 0 | 0 | 0 |
| 158 | 0 |  | 0 | 54 | 19.3 | 66.0 | 87 | 240 | 94 | 123 | 56 | 5.2 | 10.0 | 110 | 59 | 5.4 | 0 | 0 | 0 | 0 | 0 | 0 |
| 159 | 0 |  | 0 | 64 | 21.0 | 71.0 | 80 | 227 | 56 | 155 | 75 | 4.5 | 19.2 | 110 | 69 | 5.5 | 0 | 0 | 0 | 0 | 0 | 0 |
| 160 | 0 |  | 0 | 58 | 23.0 | 61.0 | 95 | 218 | 69 | 134 | 61 | 4.1 | 48.1 | 120 | 80 | 5.3 | 0 | 0 | 0 | 0 | 0 | 0 |
| 161 | 1 | 0 | 0 | 49 | 24.5 | 71.5 | 98 | 136 | 41 | 80 | 131 | 5.0 | 21.5 | 123 | 85 | 5.4 | 0 | 0 | 1 | 1 | 0 | 0 |
| 162 | 0 |  | 0 | 49 | 27.4 | 86.0 | 89 | 241 | 54 | 146 | 181 | 4.7 | 22.7 | 108 | 59 | 5.2 | 1 | 1 | 0 | 0 | 0 | 0 |
| 163 | 0 |  | 1 | 40 | 22.3 | 77.5 | 88 | 218 | 48 | 147 | 59 | 5.9 | 27.9 | 132 | 68 | 5.0 | 0 | 0 | 0 | 1 | 0 | 0 |
| 164 | 1 | 0 | 1 | 62 | 20.3 | 71.0 | 100 | 155 | 52 | 91 | 85 | 6.3 | 31.3 | 138 | 78 | 4.4 | 0 | 0 | 0 | 1 | 1 | 0 |
| 165 | 0 |  | 1 | 62 | 25.3 | 82.0 | 92 | 242 | 52 | 150 | 71 | 6.7 | 35.7 | 122 | 86 | 5.4 | 0 | 0 | 0 | 1 | 0 | 0 |
| 166 | 0 |  | 0 | 62 | 24.4 | 71.0 | 121 | 229 | 65 | 150 | 68 | 4.7 | 23.0 | 141 | 58 | 6.6 | 0 | 0 | 0 | 1 | 1 | 0 |
| 167 | 0 |  | 0 | 49 | 23.8 | 75.0 | 60 | 215 | 74 | 122 | 46 | 5.1 | 36.4 | 115 | 76 | 5.1 | 0 | 0 | 0 | 0 | 0 | 0 |
| 168 | 1 | 1 | 1 | 53 | 26.8 | 92.0 | 97 | 247 | 67 | 166 | 51 | 7.2 | 29.4 | 155 | 94 | 6.0 | 1 | 0 | 0 | 1 | 1 | 1 |
| 169 | 0 |  | 1 | 58 | 21.1 | 75.0 | 82 | 202 | 64 | 124 | 58 | 5.8 | 55.8 | 115 | 73 | 5.0 | 0 | 0 | 0 | 0 | 0 | 0 |
| 170 | 1 | 1 | 1 | 58 | 27.4 | 91.0 | 92 | 204 | 55 | 134 | 74 | 7.2 | 25.9 | 135 | 85 | 5.3 | 1 | 0 | 0 | 1 | 0 | 0 |
| 171 | 0 |  | 1 | 62 | 30.5 | 91.0 | 147 | 177 | 51 | 116 | 100 | 6.0 | 39.1 | 151 | 97 | 7.4 | 1 | 0 | 0 | 1 | 1 | 1 |
| 172 | 0 |  | 0 | 45 | 22.1 | 73.0 | 83 | 149 | 53 | 82 | 74 | 2.4 | 23.4 | 101 | 73 | 5.1 | 0 | 0 | 0 | 0 | 0 | 0 |
| 173 | 1 | 0 | 1 | 41 | 22.9 | 87.0 | 94 | 220 | 33 | 160 | 202 | 7.2 | 26.6 | 118 | 86 | 5.5 | 0 | 1 | 1 | 1 | 0 | 1 |
| 174 | 1 | 1 | 0 | 56 | 23.1 | 83.0 | 74 | 235 | 78 | 137 | 68 | 4.0 | 44.8 | 106 | 70 | 5.2 | 1 | 0 | 0 | 1 | 0 | 0 |
| 175 | 0 |  | 0 | 61 | 21.5 | 79.0 | 91 | 209 | 53 | 131 | 81 | 3.8 | 20.4 | 138 | 59 | 5.0 | 0 | 0 | 0 | 1 | 0 | 0 |
| 176 | 0 |  | 0 | 35 | 22.4 | 72.0 | 87 | 182 | 82 | 86 | 49 | 4.8 | 21.0 | 97 | 56 | 4.9 | 0 | 0 | 0 | 1 | 0 | 0 |
| 177 | 1 | 0 | 1 | 63 | 29.0 | 89.0 | 85 | 116 | 46 | 54 | 79 | 4.6 | 28.2 | 129 | 72 | 5.2 | 0 | 0 | 0 | 1 | 0 | 0 |
| 178 | 0 |  | 0 | 48 | 27.1 | 85.5 | 144 | 172 | 42 | 97 | 270 | 5.5 | 13.3 | 119 | 78 | 7.3 | 1 | 1 | 1 | 0 | 1 | 1 |
| 179 | 1 | 0 | 1 | 42 | 26.8 | 80.0 | 102 | 202 | 39 | 130 | 244 | 6.5 | 54.7 | 120 | 78 | 5.3 | 0 | 1 | 1 | 0 | 1 | 1 |
| 180 | 0 |  | 0 | 37 | 24.0 | 84.0 | 81 | 164 | 64 | 86 | 68 | 4.5 | 24.8 | 113 | 67 | 5.2 | 1 | 0 | 0 | 0 | 0 | 0 |
| 181 | 0 |  | 0 | 49 | 21.2 | 78.0 | 91 | 253 | 89 | 143 | 104 | 4.7 | 19.9 | 100 | 63 | 5.2 | 0 | 0 | 0 | 0 | 0 | 0 |
| 182 | 0 |  | 0 | 40 | 20.6 | 68.0 | 78 | 162 | 79 | 70 | 51 | 4.4 | 30.8 | 95 | 58 | 5.3 | 0 | 0 | 0 | 1 | 0 | 0 |
| 183 | 0 |  | 1 | 40 | 28.6 | 102.5 | 93 | 241 | 53 | 157 | 136 | 7.6 | 39.8 | 128 | 76 | 5.3 | 1 | 0 | 0 | 0 | 0 | 0 |
| 184 | 0 |  | 0 | 39 | 23.4 | 75.0 | 88 | 147 | 53 | 82 | 49 | 5.2 | 16.6 | 110 | 71 | 5.4 | 0 | 0 | 0 | 0 | 0 | 0 |
| 185 | 1 | 0 | 0 | 45 | 20.0 | 66.0 | 84 | 165 | 54 | 98 | 71 | 3.5 | 6.8 | 89 | 61 | 4.9 | 0 | 0 | 0 | 1 | 0 | 0 |
| 186 | 0 |  | 1 | 53 | 24.8 | 80.0 | 93 | 203 | 43 | 128 | 188 | 6.5 | 30.7 | 108 | 79 | 5.4 | 0 | 1 | 0 | 0 | 0 | 0 |
| 187 | 1 | 1 | 1 | 61 | 24.7 | 84.0 | 121 | 131 | 39 | 84 | 68 | 5.8 | 36.9 | 130 | 72 | 7.3 | 0 | 0 | 1 | 1 | 1 | 1 |
| 188 | 1 | 1 | 1 | 63 | 25.5 | 83.0 | 89 | 166 | 47 | 101 | 81 | 5.6 | 30.6 | 149 | 97 | 5.9 | 0 | 0 | 0 | 1 | 1 | 0 |
| 189 | 0 |  | 0 | 58 | 21.2 | 69.5 | 76 | 227 | 64 | 151 | 51 | 6.3 | 19.8 | 98 | 60 | 5.5 | 0 | 0 | 0 | 1 | 0 | 0 |
| 190 | 0 |  | 0 | 36 | 19.7 | 70.0 | 72 | 183 | 56 | 115 | 52 | 3.1 | 11.4 | 128 | 80 | 4.7 | 0 | 0 | 0 | 0 | 0 | 0 |
| 191 | 1 | 1 | 1 | 44 | 22.4 | 90.0 | 84 | 187 | 42 | 129 | 75 | 5.2 | 24.5 | 117 | 86 | 5.4 | 1 | 0 | 0 | 1 | 0 | 0 |
| 192 | 0 |  | 1 | 43 | 21.9 | 76.0 | 91 | 150 | 38 | 96 | 120 | 4.3 | 27.8 | 116 | 80 | 5.5 | 0 | 0 | 1 | 0 | 0 | 0 |
| 193 | 0 |  | 1 | 53 | 34.6 | 108.0 | 172 | 288 | 33 | 123 | 959 | 9.0 | 33.2 | 156 | 89 | 8.9 | 1 | 1 | 1 | 1 | 1 | 1 |
| 194 | 0 |  | 1 | 60 | 24.1 | 78.0 | 92 | 210 | 61 | 126 | 90 | 6.9 | 32.3 | 117 | 78 | 5.6 | 0 | 0 | 0 | 0 | 0 | 0 |
| 195 | 1 | 0 | 0 | 52 | 25.2 | 82.0 | 209 | 145 | 45 | 81 | 129 | 4.7 | 22.3 | 126 | 91 | 7.4 | 1 | 0 | 1 | 1 | 1 | 1 |
| 196 | 1 | 1 | 0 | 35 | 20.3 | 72.0 | 86 | 170 | 53 | 104 | 84 | 3.9 | 24.4 | 106 | 64 | 5.5 | 0 | 0 | 0 | 0 | 0 | 0 |
| 197 | 1 | 0 | 1 | 46 | 25.0 | 86.0 | 88 | 254 | 76 | 160 | 157 | 5.6 | 25.7 | 126 | 79 | 4.9 | 0 | 1 | 0 | 0 | 0 | 0 |
| 198 | 0 |  | 1 | 44 | 23.0 | 77.0 | 93 | 211 | 25 | 108 | 282 | 5.8 | 28.9 | 117 | 82 | 5.3 | 0 | 1 | 1 | 0 | 0 | 0 |
| 199 | 1 | 1 | 1 | 44 | 27.9 | 85.0 | 101 | 253 | 31 | 143 | 414 | 6.7 | 23.9 | 113 | 78 | 5.4 | 0 | 1 | 1 | 0 | 1 | 1 |
| 200 | 0 |  | 0 | 46 | 20.8 | 67.5 | 88 | 218 | 55 | 151 | 84 | 4.0 | 37.0 | 105 | 65 | 4.9 | 0 | 0 | 0 | 0 | 0 | 0 |
| 201 | 0 |  | 1 | 56 | 26.6 | 97.0 | 163 | 189 | 46 | 114 | 212 | 7.2 | 29.7 | 159 | 98 | 8.1 | 1 | 1 | 0 | 1 | 1 | 1 |
| 202 | 0 |  | 1 | 40 | 22.5 | 76.0 | 93 | 184 | 72 | 92 | 36 | 6.7 | 31.7 | 111 | 70 | 5.7 | 0 | 0 | 0 | 0 | 0 | 0 |
| 203 | 1 | 1 | 1 | 57 | 26.3 | 95.0 | 99 | 274 | 36 | 211 | 147 | 7.9 | 32.0 | 126 | 81 | 5.6 | 1 | 0 | 1 | 0 | 0 | 0 |
| 204 | 1 | 0 | 1 | 61 | 31.5 | 110.0 | 112 | 147 | 40 | 88 | 86 | 5.9 | 21.7 | 149 | 85 | 6.3 | 1 | 0 | 0 | 1 | 1 | 1 |
| 205 | 0 |  | 0 | 46 | 20.6 | 72.0 | 87 | 178 | 52 | 118 | 54 | 4.8 | 29.0 | 117 | 74 | 5.3 | 0 | 0 | 0 | 0 | 0 | 0 |
| 206 | 0 |  | 0 | 51 | 21.7 | 67.0 | 91 | 188 | 81 | 99 | 70 | 3.9 | 23.4 | 117 | 68 | 5.0 | 0 | 0 | 0 | 0 | 0 | 0 |
| 207 | 0 |  | 0 | 59 | 30.4 | 78.0 | 101 | 229 | 45 | 145 | 141 | 6.0 | 33.2 | 112 | 66 | 5.9 | 0 | 0 | 1 | 1 | 1 | 1 |
| 208 | 0 |  | 0 | 42 | 19.9 | 70.0 | 82 | 220 | 71 | 135 | 61 | 4.7 | 24.3 | 110 | 72 | 5.8 | 0 | 0 | 0 | 0 | 1 | 0 |
| 209 | 0 |  | 1 | 64 | 19.8 | 72.0 | 88 | 156 | 42 | 101 | 81 | 7.3 | 8.9 | 115 | 75 | 5.1 | 0 | 0 | 0 | 1 | 0 | 0 |
| 210 | 1 | 0 | 1 | 41 | 32.0 | 97.0 | 89 | 183 | 32 | 117 | 156 | 8.1 | 30.9 | 140 | 75 | 5.4 | 1 | 1 | 1 | 1 | 0 | 1 |
| 211 | 0 |  | 1 | 44 | 26.8 | 85.5 | 95 | 165 | 39 | 103 | 180 | 8.4 | 20.2 | 112 | 75 | 5.4 | 0 | 1 | 1 | 0 | 0 | 0 |
| 212 | 1 | 0 | 0 | 47 | 26.9 | 85.0 | 98 | 196 | 47 | 146 | 90 | 5.3 | 13.5 | 130 | 79 | 5.3 | 1 | 0 | 1 | 1 | 0 | 1 |
| 213 | 0 |  | 0 | 44 | 22.2 | 70.0 | 100 | 184 | 50 | 112 | 121 | 5.2 | 20.2 | 109 | 66 | 5.4 | 0 | 0 | 0 | 0 | 1 | 0 |
| 214 | 1 | 0 | 1 | 44 | 27.9 | 93.0 | 95 | 218 | 36 | 125 | 247 | 5.8 | 17.2 | 137 | 86 | 5.5 | 1 | 1 | 1 | 1 | 0 | 1 |
| 215 | 0 |  | 0 | 48 | 19.9 | 69.0 | 92 | 208 | 63 | 120 | 113 | 3.7 | 24.4 | 125 | 75 | 5.2 | 0 | 0 | 0 | 0 | 0 | 0 |
| 216 | 0 |  | 1 | 30 | 20.5 | 71.0 | 82 | 166 | 55 | 97 | 76 | 6.8 | 18.2 | 114 | 71 | 5.0 | 0 | 0 | 0 | 0 | 0 | 0 |
| 217 | 1 | 0 | 0 | 58 | 21.0 | 75.0 | 92 | 225 | 60 | 135 | 73 | 4.5 | 30.4 | 110 | 69 | 5.5 | 0 | 0 | 0 | 0 | 0 | 0 |
| 218 | 0 |  | 1 | 30 | 20.9 | 69.0 | 83 | 172 | 50 | 106 | 50 | 6.5 | 31.3 | 100 | 60 | 5.4 | 0 | 0 | 0 | 0 | 0 | 0 |
| 219 | 0 |  | 0 | 38 | 20.1 | 65.0 | 90 | 234 | 55 | 156 | 104 | 5.3 | 20.0 | 118 | 77 | 5.4 | 0 | 0 | 0 | 0 | 0 | 0 |
| 220 | 1 | 1 | 1 | 49 | 36.2 | 112.0 | 128 | 123 | 24 | 72 | 192 | 6.0 | 36.5 | 127 | 83 | 6.9 | 1 | 1 | 1 | 1 | 1 | 1 |
| 221 | 1 | 0 | 1 | 46 | 20.4 | 70.0 | 138 | 153 | 55 | 83 | 104 | 5.6 | 36.0 | 138 | 87 | 4.4 | 0 | 0 | 0 | 1 | 1 | 0 |
| 222 | 1 | 0 | 1 | 53 | 25.5 | 82.0 | 99 | 186 | 49 | 127 | 82 | 3.9 | 31.2 | 115 | 77 | 5.4 | 0 | 0 | 0 | 0 | 0 | 0 |
| 223 | 1 | 1 | 1 | 49 | 26.9 | 89.0 | 90 | 245 | 43 | 164 | 201 | 6.5 | 23.5 | 125 | 80 | 5.4 | 0 | 1 | 0 | 1 | 0 | 0 |
| 224 | 0 |  | 1 | 37 | 25.5 | 91.0 | 102 | 174 | 47 | 107 | 98 | 8.7 | 21.6 | 107 | 68 | 5.4 | 1 | 0 | 0 | 0 | 1 | 0 |
| 225 | 0 |  | 1 | 47 | 17.5 | 64.0 | 88 | 222 | 92 | 116 | 44 | 4.7 | 43.7 | 115 | 66 | 5.0 | 0 | 0 | 0 | 0 | 0 | 0 |
| 226 | 0 |  | 1 | 45 | 28.1 | 92.0 | 96 | 217 | 41 | 151 | 138 | 6.6 | 34.6 | 123 | 84 | 4.9 | 1 | 0 | 0 | 0 | 0 | 0 |
| 227 | 1 | 1 | 1 | 63 | 26.2 | 91.0 | 103 | 216 | 72 | 122 | 102 | 6.4 | 36.0 | 115 | 62 | 6.0 | 1 | 0 | 0 | 1 | 1 | 1 |
| 228 | 1 | 0 | 1 | 59 | 24.3 | 87.0 | 118 | 204 | 39 | 140 | 120 | 6.2 | 39.4 | 116 | 75 | 6.0 | 0 | 0 | 1 | 0 | 1 | 0 |
| 229 | 0 |  | 0 | 41 | 24.0 | 85.0 | 102 | 218 | 68 | 124 | 76 | 4.9 | 43.2 | 107 | 67 | 5.3 | 1 | 0 | 0 | 0 | 1 | 0 |
| 230 | 1 | 0 | 0 | 38 | 22.7 | 71.0 | 83 | 137 | 57 | 66 | 78 | 5.0 | 27.5 | 93 | 66 | 5.0 | 0 | 0 | 0 | 1 | 0 | 0 |
| 231 | 0 |  | 0 | 47 | 20.5 | 71.0 | 106 | 178 | 38 | 115 | 163 | 6.3 | 24.5 | 147 | 87 | 5.3 | 0 | 1 | 1 | 1 | 1 | 1 |
| 232 | 1 | 1 | 1 | 62 | 27.6 | 89.0 | 97 | 165 | 42 | 91 | 185 | 5.7 | 41.1 | 161 | 91 | 5.5 | 0 | 1 | 0 | 1 | 0 | 0 |
| 233 | 1 | 1 | 1 | 44 | 23.7 | 86.0 | 98 | 149 | 35 | 103 | 62 | 6.3 | 16.2 | 140 | 81 | 5.4 | 0 | 0 | 1 | 1 | 0 | 0 |
| 234 | 0 |  | 0 | 36 | 22.8 | 78.0 | 90 | 215 | 49 | 137 | 131 | 4.4 | 23.5 | 120 | 72 | 5.3 | 0 | 0 | 1 | 0 | 0 | 0 |
| 235 | 0 |  | 0 | 36 | 24.7 | 75.0 | 97 | 204 | 60 | 130 | 75 | 4.8 | 31.3 | 111 | 67 | 5.6 | 0 | 0 | 0 | 0 | 0 | 0 |
| 236 | 1 | 1 | 1 | 53 | 18.8 | 73.0 | 78 | 246 | 77 | 142 | 103 | 9.8 | 19.9 | 125 | 101 | 5.1 | 0 | 0 | 0 | 1 | 0 | 0 |
| 237 | 1 | 0 | 1 | 57 | 22.5 | 74.0 | 92 | 288 | 54 | 201 | 59 | 7.2 | 38.2 | 129 | 84 | 5.0 | 0 | 0 | 0 | 0 | 0 | 0 |
| 238 | 0 |  | 0 | 37 | 23.7 | 78.0 | 98 | 232 | 45 | 166 | 142 | 5.7 | 18.1 | 108 | 71 | 5.5 | 0 | 0 | 1 | 0 | 0 | 0 |
| 239 | 0 |  | 0 | 36 | 22.5 | 77.0 | 75 | 173 | 47 | 114 | 58 | 5.5 | 32.4 | 102 | 65 | 5.3 | 0 | 0 | 1 | 0 | 0 | 0 |
| 240 | 1 | 0 | 0 | 63 | 26.8 | 86.5 | 126 | 153 | 41 | 86 | 178 | 5.5 | 22.8 | 125 | 50 | 6.6 | 1 | 1 | 1 | 1 | 1 | 1 |
| 241 | 0 |  | 0 | 36 | 20.6 | 72.0 | 87 | 180 | 67 | 112 | 74 | 4.0 | 10.0 | 99 | 54 | 4.9 | 0 | 0 | 0 | 1 | 0 | 0 |
| 242 | 0 |  | 0 | 46 | 19.9 | 65.0 | 87 | 167 | 60 | 97 | 29 | 4.4 | 25.1 | 130 | 68 | 4.9 | 0 | 0 | 0 | 1 | 0 | 0 |
| 243 | 1 | 1 | 1 | 55 | 21.9 | 75.0 | 94 | 182 | 58 | 101 | 94 | 4.6 | 26.4 | 136 | 90 | 5.0 | 0 | 0 | 0 | 1 | 0 | 0 |
| 244 | 1 | 0 | 1 | 43 | 25.6 | 88.0 | 116 | 189 | 35 | 116 | 217 | 6.4 | 14.4 | 135 | 93 | 6.5 | 0 | 1 | 1 | 1 | 1 | 1 |
| 245 | 1 | 0 | 1 | 44 | 30.8 | 102.0 | 109 | 174 | 50 | 98 | 170 | 7.2 | 54.4 | 122 | 84 | 5.5 | 1 | 1 | 0 | 0 | 1 | 1 |
| 246 | 0 |  | 0 | 50 | 24.8 | 70.0 | 91 | 175 | 39 | 115 | 116 | 5.5 | 17.6 | 126 | 64 | 4.5 | 0 | 0 | 1 | 0 | 0 | 0 |
| 247 | 1 | 0 | 1 | 58 | 25.2 | 86.0 | 112 | 210 | 42 | 137 | 121 | 5.3 | 20.7 | 124 | 76 | 6.1 | 0 | 0 | 0 | 0 | 1 | 0 |
| 248 | 0 |  | 0 | 46 | 20.9 | 70.0 | 88 | 166 | 35 | 113 | 93 | 5.1 | 25.2 | 104 | 64 | 5.4 | 0 | 0 | 1 | 0 | 0 | 0 |
| 249 | 0 |  | 1 | 54 | 22.6 | 84.0 | 89 | 155 | 37 | 100 | 120 | 6.4 | 18.3 | 131 | 98 | 5.6 | 0 | 0 | 1 | 1 | 0 | 0 |
| 250 | 0 |  | 1 | 53 | 27.2 | 87.0 | 90 | 235 | 37 | 157 | 149 | 10.9 | 36.1 | 135 | 76 | 5.5 | 0 | 0 | 1 | 1 | 0 | 0 |
| 251 | 0 |  | 0 | 51 | 24.8 | 71.0 | 97 | 265 | 57 | 191 | 42 | 4.6 | 20.2 | 159 | 94 | 5.4 | 0 | 0 | 0 | 1 | 0 | 0 |
| 252 | 0 |  | 1 | 61 | 23.4 | 75.0 | 76 | 264 | 78 | 162 | 64 | 6.4 | 38.1 | 134 | 77 | 5.7 | 0 | 0 | 0 | 1 | 0 | 0 |
| 253 | 0 |  | 1 | 52 | 26.8 | 81.0 | 104 | 229 | 37 | 175 | 149 | 7.1 | 15.3 | 120 | 77 | 5.4 | 0 | 0 | 1 | 0 | 1 | 0 |
| 254 | 0 |  | 1 | 52 | 25.2 | 85.0 | 90 | 229 | 39 | 150 | 201 | 6.5 | 23.7 | 125 | 84 | 5.4 | 0 | 1 | 1 | 0 | 0 | 0 |
| 255 | 0 |  | 1 | 34 | 32.7 | 107.0 | 92 | 209 | 46 | 146 | 132 | 7.3 | 25.2 | 126 | 83 | 5.5 | 1 | 0 | 0 | 0 | 0 | 0 |
| 256 | 0 |  | 1 | 42 | 27.2 | 84.0 | 84 | 154 | 43 | 99 | 45 | 7.0 | 23.9 | 125 | 79 | 4.7 | 0 | 0 | 0 | 0 | 0 | 0 |
| 257 | 1 | 0 | 1 | 56 | 29.6 | 104.5 | 107 | 179 | 52 | 95 | 110 | 4.7 | 19.2 | 128 | 82 | 5.6 | 1 | 0 | 0 | 1 | 1 | 1 |
| 258 | 0 |  | 0 | 43 | 16.2 | 64.0 | 91 | 196 | 65 | 123 | 67 | 5.0 | 19.5 | 108 | 72 | 5.3 | 0 | 0 | 0 | 0 | 0 | 0 |
| 259 | 0 |  | 0 | 47 | 23.9 | 73.0 | 81 | 168 | 65 | 91 | 55 | 4.7 | 27.5 | 124 | 81 | 5.0 | 0 | 0 | 0 | 0 | 0 | 0 |
| 260 | 0 |  | 0 | 61 | 29.7 | 88.0 | 95 | 168 | 57 | 98 | 50 | 5.6 | 25.5 | 143 | 74 | 5.5 | 1 | 0 | 0 | 1 | 0 | 0 |
| 261 | 1 | 0 | 1 | 61 | 25.1 | 89.0 | 103 | 137 | 35 | 83 | 68 | 7.7 | 24.5 | 133 | 80 | 5.3 | 0 | 0 | 1 | 1 | 1 | 1 |
| 262 | 0 |  | 1 | 48 | 27.4 | 88.5 | 101 | 282 | 52 | 210 | 170 | 5.8 | 56.4 | 106 | 74 | 5.8 | 0 | 1 | 0 | 0 | 1 | 0 |
| 263 | 0 |  | 0 | 38 | 21.7 | 73.0 | 77 | 162 | 41 | 113 | 144 | 4.7 | 15.5 | 101 | 61 | 5.1 | 0 | 0 | 1 | 0 | 0 | 0 |
| 264 | 0 |  | 1 | 55 | 23.9 | 72.0 | 98 | 237 | 68 | 148 | 48 | 7.5 | 24.4 | 114 | 76 | 5.7 | 0 | 0 | 0 | 0 | 0 | 0 |
| 265 | 0 |  | 0 | 42 | 22.2 | 65.0 | 89 | 192 | 74 | 102 | 38 | 5.0 | 22.6 | 108 | 65 | 4.9 | 0 | 0 | 0 | 0 | 0 | 0 |
| 266 | 0 |  | 0 | 48 | 22.4 | 70.0 | 83 | 188 | 70 | 101 | 86 | 3.3 | 39.1 | 137 | 95 | 5.0 | 0 | 0 | 0 | 1 | 0 | 0 |
| 267 | 0 |  | 1 | 45 | 27.1 | 94.0 | 98 | 216 | 41 | 135 | 223 | 7.0 | 36.5 | 152 | 96 | 5.9 | 1 | 1 | 0 | 1 | 1 | 1 |
| 268 | 0 |  | 0 | 47 | 19.7 | 69.0 | 92 | 187 | 50 | 123 | 51 | 4.3 | 20.9 | 109 | 71 | 4.9 | 0 | 0 | 0 | 0 | 0 | 0 |
| 269 | 1 | 0 | 1 | 53 | 24.9 | 81.0 | 90 | 150 | 58 | 82 | 50 | 5.5 | 19.2 | 124 | 82 | 6.2 | 0 | 0 | 0 | 1 | 1 | 0 |
| 270 | 1 | 1 | 1 | 63 | 20.0 | 84.0 | 100 | 220 | 92 | 81 | 251 | 7.1 | 36.3 | 154 | 84 | 5.6 | 0 | 1 | 0 | 1 | 1 | 1 |
| 271 | 0 |  | 1 | 45 | 23.6 | 79.5 | 82 | 223 | 39 | 148 | 175 | 8.0 | 24.2 | 120 | 81 | 3.9 | 0 | 1 | 1 | 0 | 0 | 0 |
| 272 | 1 | 0 | 1 | 58 | 25.7 | 85.0 | 90 | 275 | 46 | 193 | 133 | 6.0 | 17.6 | 132 | 84 | 3.8 | 0 | 0 | 0 | 1 | 0 | 0 |
| 273 | 1 | 0 | 0 | 53 | 20.4 | 74.0 | 111 | 292 | 44 | 212 | 181 | 6.5 | 32.8 | 137 | 90 | 5.7 | 0 | 1 | 1 | 1 | 1 | 1 |
| 274 | 1 | 1 | 1 | 51 | 30.9 | 97.0 | 101 | 213 | 50 | 143 | 90 | 9.3 | 31.8 | 130 | 83 | 5.8 | 1 | 0 | 0 | 1 | 1 | 1 |
| 275 | 0 |  | 1 | 51 | 22.5 | 81.0 | 77 | 193 | 44 | 129 | 72 | 6.8 | 34.4 | 140 | 88 | 5.0 | 0 | 0 | 0 | 1 | 0 | 0 |
| 276 | 1 | 0 | 0 | 43 | 31.6 | 84.0 | 106 | 251 | 53 | 174 | 114 | 5.5 | 16.2 | 123 | 73 | 5.3 | 1 | 0 | 0 | 1 | 1 | 1 |
| 277 | 0 |  | 0 | 60 | 26.1 | 87.0 | 97 | 257 | 60 | 167 | 109 | 6.6 | 22.9 | 121 | 64 | 7.2 | 1 | 0 | 0 | 0 | 1 | 0 |
| 278 | 0 |  | 1 | 52 | 24.3 | 77.0 | 99 | 167 | 53 | 93 | 152 | 7.1 | 25.5 | 114 | 79 | 5.3 | 0 | 1 | 0 | 0 | 0 | 0 |
| 279 | 1 | 1 | 1 | 52 | 29.6 | 95.0 | 91 | 176 | 31 | 133 | 63 | 6.4 | 51.9 | 111 | 69 | 5.6 | 1 | 0 | 1 | 0 | 0 | 0 |
| 280 | 0 |  | 0 | 61 | 24.7 | 82.0 | 108 | 145 | 42 | 88 | 76 | 5.4 | 23.6 | 176 | 83 | 5.7 | 1 | 0 | 1 | 1 | 1 | 1 |
| 281 | 1 | 1 | 1 | 58 | 24.9 | 84.0 | 96 | 204 | 57 | 125 | 130 | 6.1 | 19.5 | 114 | 71 | 5.7 | 0 | 0 | 0 | 0 | 0 | 0 |
| 282 | 0 |  | 0 | 48 | 19.7 | 69.0 | 97 | 174 | 59 | 93 | 57 | 5.3 | 25.2 | 92 | 59 | 5.4 | 0 | 0 | 0 | 1 | 0 | 0 |
| 283 | 1 | 1 | 1 | 53 | 26.2 | 88.0 | 95 | 191 | 40 | 120 | 132 | 6.1 | 69.0 | 115 | 80 | 5.6 | 0 | 0 | 0 | 0 | 0 | 0 |
| 284 | 1 | 0 | 0 | 42 | 24.3 | 78.0 | 106 | 187 | 32 | 55 | 585 | 7.0 | 17.8 | 140 | 98 | 5.9 | 0 | 1 | 1 | 1 | 1 | 1 |
| 285 | 0 |  | 0 | 50 | 17.7 | 67.0 | 83 | 155 | 52 | 68 | 192 | 4.3 | 25.8 | 110 | 66 | 5.4 | 0 | 1 | 0 | 0 | 0 | 0 |
| 286 | 0 |  | 0 | 41 | 25.0 | 77.0 | 84 | 229 | 55 | 159 | 61 | 5.2 | 23.5 | 131 | 89 | 5.1 | 0 | 0 | 0 | 1 | 0 | 0 |
| 287 | 0 |  | 1 | 37 | 25.1 | 85.0 | 90 | 262 | 43 | 184 | 176 | 7.8 | 48.7 | 134 | 83 | 5.3 | 0 | 1 | 0 | 1 | 0 | 0 |
| 288 | 1 | 0 | 1 | 54 | 27.5 | 99.0 | 92 | 273 | 44 | 208 | 124 | 5.9 | 40.4 | 124 | 80 | 5.4 | 1 | 0 | 0 | 1 | 0 | 0 |
| 289 | 0 |  | 1 | 51 | 26.7 | 90.0 | 106 | 173 | 29 | 86 | 322 | 6.7 | 30.4 | 112 | 65 | 5.5 | 1 | 1 | 1 | 0 | 1 | 1 |
| 290 | 0 |  | 0 | 49 | 19.5 | 64.0 | 86 | 227 | 72 | 136 | 68 | 4.9 | 31.7 | 93 | 61 | 5.2 | 0 | 0 | 0 | 1 | 0 | 0 |
| 291 | 0 |  | 0 | 42 | 21.2 | 67.5 | 94 | 177 | 65 | 97 | 62 | 4.5 | 29.7 | 94 | 66 | 5.4 | 0 | 0 | 0 | 1 | 0 | 0 |
| 292 | 0 |  | 0 | 57 | 27.3 | 76.0 | 111 | 272 | 55 | 191 | 122 | 6.4 | 45.0 | 145 | 79 | 5.5 | 0 | 0 | 0 | 1 | 1 | 0 |
| 293 | 0 |  | 0 | 36 | 21.7 | 70.0 | 93 | 165 | 40 | 109 | 108 | 4.3 | 15.8 | 99 | 62 | 5.3 | 0 | 0 | 1 | 1 | 0 | 0 |
| 294 | 0 |  | 0 | 33 | 21.3 | 74.0 | 99 | 170 | 56 | 104 | 81 | 4.4 | 35.3 | 113 | 69 | 4.8 | 0 | 0 | 0 | 0 | 0 | 0 |
| 295 | 0 |  | 1 | 37 | 26.8 | 83.0 | 87 | 205 | 64 | 120 | 87 | 7.2 | 17.9 | 128 | 81 | 5.0 | 0 | 0 | 0 | 0 | 0 | 0 |
| 296 | 0 |  | 0 | 36 | 21.3 | 72.0 | 82 | 180 | 76 | 88 | 61 | 3.0 | 30.6 | 98 | 62 | 5.0 | 0 | 0 | 0 | 1 | 0 | 0 |
| 297 | 1 | 1 | 1 | 55 | 23.8 | 85.0 | 91 | 209 | 39 | 144 | 168 | 7.4 | 31.9 | 129 | 85 | 5.5 | 0 | 1 | 1 | 1 | 0 | 1 |
| 298 | 1 | 1 | 1 | 54 | 23.2 | 84.0 | 96 | 208 | 41 | 107 | 204 | 6.6 | 20.6 | 125 | 84 | 5.3 | 0 | 1 | 0 | 0 | 0 | 0 |
| 299 | 0 |  | 1 | 35 | 22.5 | 80.0 | 85 | 169 | 45 | 115 | 69 | 5.6 | 19.3 | 116 | 74 | 4.9 | 0 | 0 | 0 | 0 | 0 | 0 |
| 300 | 0 |  | 0 | 62 | 27.9 | 87.0 | 91 | 225 | 59 | 129 | 97 | 5.0 | 36.7 | 142 | 79 | 5.2 | 1 | 0 | 0 | 1 | 0 | 0 |
| 301 | 0 |  | 1 | 62 | 25.3 | 93.0 | 101 | 129 | 40 | 80 | 50 | 7.2 | 41.7 | 128 | 82 | 5.7 | 1 | 0 | 0 | 1 | 1 | 1 |
| 302 | 0 |  | 0 | 44 | 21.3 | 70.0 | 73 | 211 | 65 | 113 | 78 | 4.3 | 21.9 | 104 | 66 | 5.3 | 0 | 0 | 0 | 0 | 0 | 0 |
| 303 | 1 | 1 | 1 | 56 | 21.2 | 76.0 | 102 | 222 | 50 | 159 | 180 | 5.6 | 50.7 | 130 | 84 | 5.3 | 0 | 1 | 0 | 1 | 1 | 1 |
| 304 | 1 | 0 | 0 | 46 | 19.5 | 62.0 | 96 | 181 | 40 | 106 | 146 | 3.5 | 27.5 | 114 | 58 | 5.7 | 0 | 0 | 1 | 0 | 0 | 0 |
| 305 | 1 | 0 | 1 | 40 | 28.2 | 97.0 | 103 | 285 | 45 | 212 | 163 | 5.7 | 21.3 | 126 | 80 | 5.6 | 1 | 1 | 0 | 0 | 1 | 1 |
| 306 | 0 |  | 0 | 58 | 26.9 | 85.0 | 113 | 206 | 48 | 137 | 129 | 6.6 | 33.5 | 122 | 68 | 5.7 | 1 | 0 | 1 | 1 | 1 | 1 |
| 307 | 0 |  | 0 | 51 | 22.2 | 73.0 | 92 | 183 | 67 | 104 | 44 | 5.3 | 26.0 | 113 | 75 | 5.0 | 0 | 0 | 0 | 0 | 0 | 0 |
| 308 | 0 |  | 1 | 55 | 21.2 | 74.0 | 92 | 198 | 68 | 116 | 79 | 5.3 | 28.3 | 121 | 77 | 5.1 | 0 | 0 | 0 | 0 | 0 | 0 |
| 309 | 0 |  | 1 | 61 | 23.6 | 86.0 | 89 | 267 | 63 | 166 | 117 | 7.2 | 40.5 | 124 | 72 | 5.7 | 0 | 0 | 0 | 0 | 0 | 0 |
| 310 | 0 |  | 1 | 60 | 20.9 | 76.5 | 82 | 222 | 54 | 156 | 41 | 6.4 | 38.9 | 134 | 89 | 5.6 | 0 | 0 | 0 | 1 | 0 | 0 |
| 311 | 0 |  | 0 | 54 | 21.1 | 69.0 | 97 | 184 | 67 | 95 | 74 | 4.6 | 22.3 | 112 | 65 | 5.2 | 0 | 0 | 0 | 0 | 0 | 0 |
| 312 | 1 | 1 | 1 | 47 | 30.6 | 102.0 | 113 | 218 | 44 | 95 | 513 | 6.5 | 60.1 | 122 | 73 | 5.9 | 1 | 1 | 0 | 1 | 1 | 1 |
| 313 | 0 |  | 1 | 52 | 27.1 | 85.0 | 87 | 275 | 51 | 166 | 242 | 7.1 | 20.1 | 104 | 69 | 5.5 | 0 | 1 | 0 | 0 | 0 | 0 |
| 314 | 0 |  | 0 | 31 | 18.7 | 60.5 | 68 | 174 | 71 | 79 | 39 | 5.4 | 25.2 | 111 | 76 | 5.0 | 0 | 0 | 0 | 0 | 0 | 0 |
| 315 | 0 |  | 1 | 38 | 30.8 | 98.0 | 158 | 140 | 31 | 69 | 278 | 6.7 | 23.6 | 134 | 87 | 7.1 | 1 | 1 | 1 | 1 | 1 | 1 |
| 316 | 0 |  | 1 | 39 | 25.3 | 92.5 | 102 | 193 | 43 | 129 | 123 | 6.0 | 20.7 | 131 | 77 | 5.0 | 1 | 0 | 0 | 1 | 1 | 1 |
| 317 | 0 |  | 1 | 52 | 24.3 | 81.5 | 87 | 235 | 44 | 175 | 119 | 5.8 | 29.2 | 138 | 83 | 5.1 | 0 | 0 | 0 | 1 | 0 | 0 |
| 318 | 0 |  | 1 | 62 | 27.0 | 85.0 | 98 | 198 | 58 | 130 | 62 | 6.0 | 38.9 | 123 | 79 | 5.6 | 0 | 0 | 0 | 0 | 0 | 0 |
| 319 | 1 | 0 | 1 | 52 | 27.5 | 90.0 | 104 | 286 | 44 | 199 | 325 | 6.6 | 19.4 | 146 | 94 | 5.4 | 1 | 1 | 0 | 1 | 1 | 1 |
| 320 | 1 | 0 | 1 | 45 | 22.5 | 80.0 | 97 | 147 | 39 | 91 | 76 | 6.6 | 18.8 | 114 | 76 | 5.0 | 0 | 0 | 1 | 0 | 0 | 0 |
| 321 | 0 |  | 0 | 48 | 27.1 | 80.0 | 91 | 273 | 38 | 203 | 201 | 5.2 | 45.4 | 116 | 77 | 5.2 | 1 | 1 | 1 | 0 | 0 | 1 |
| 322 | 1 | 1 | 1 | 42 | 27.4 | 92.5 | 82 | 236 | 36 | 179 | 106 | 7.7 | 27.8 | 125 | 88 | 5.6 | 1 | 0 | 1 | 1 | 0 | 1 |
| 323 | 0 |  | 0 | 37 | 22.2 | 68.0 | 87 | 162 | 53 | 101 | 65 | 4.0 | 13.3 | 96 | 59 | 5.3 | 0 | 0 | 0 | 1 | 0 | 0 |
| 324 | 0 |  | 0 | 51 | 27.0 | 87.0 | 92 | 182 | 58 | 108 | 92 | 5.1 | 21.5 | 121 | 67 | 5.6 | 1 | 0 | 0 | 0 | 0 | 0 |
| 325 | 0 |  | 0 | 38 | 24.1 | 83.0 | 87 | 93 | 56 | 33 | 32 | 5.4 | 15.3 | 102 | 54 | 5.5 | 1 | 0 | 0 | 0 | 0 | 0 |
| 326 | 0 |  | 1 | 38 | 27.0 | 93.0 | 100 | 193 | 35 | 142 | 61 | 6.5 | 24.8 | 113 | 76 | 5.1 | 1 | 0 | 1 | 0 | 1 | 1 |
| 327 | 0 |  | 1 | 62 | 26.7 | 96.0 | 90 | 182 | 32 | 132 | 103 | 7.0 | 37.6 | 115 | 76 | 6.1 | 1 | 0 | 1 | 0 | 1 | 1 |
| 328 | 1 | 0 | 1 | 60 | 23.4 | 80.0 | 83 | 126 | 38 | 72 | 38 | 6.6 | 20.6 | 142 | 87 | 4.9 | 0 | 0 | 1 | 1 | 0 | 0 |
| 329 | 0 |  | 1 | 47 | 23.8 | 82.0 | 93 | 232 | 52 | 155 | 104 | 5.2 | 21.0 | 120 | 71 | 5.1 | 0 | 0 | 0 | 0 | 0 | 0 |
| 330 | 0 |  | 1 | 48 | 29.4 | 96.0 | 102 | 223 | 35 | 176 | 101 | 5.1 | 36.2 | 127 | 94 | 5.9 | 1 | 0 | 1 | 1 | 1 | 1 |
| 331 | 1 | 1 | 0 | 50 | 21.5 | 68.5 | 96 | 170 | 79 | 76 | 38 | 3.9 | 16.6 | 104 | 65 | 5.3 | 0 | 0 | 0 | 0 | 0 | 0 |
| 332 | 1 | 0 | 1 | 37 | 26.5 | 90.5 | 91 | 178 | 43 | 120 | 76 | 7.3 | 27.1 | 128 | 81 | 4.9 | 1 | 0 | 0 | 0 | 0 | 0 |
| 333 | 0 |  | 1 | 42 | 18.5 | 60.0 | 78 | 185 | 52 | 122 | 53 | 6.0 | 16.8 | 117 | 77 | 4.8 | 0 | 0 | 0 | 0 | 0 | 0 |
| 334 | 1 | 0 | 1 | 56 | 27.2 | 92.0 | 81 | 208 | 32 | 159 | 94 | 7.1 | 35.6 | 118 | 80 | 5.2 | 1 | 0 | 1 | 0 | 0 | 0 |
| 335 | 1 | 1 | 1 | 56 | 23.6 | 86.0 | 120 | 141 | 40 | 67 | 213 | 7.1 | 47.2 | 112 | 70 | 5.9 | 0 | 1 | 0 | 1 | 1 | 1 |
| 336 | 0 |  | 1 | 59 | 19.3 | 75.0 | 106 | 174 | 47 | 101 | 114 | 5.8 | 30.3 | 110 | 55 | 5.7 | 0 | 0 | 0 | 0 | 1 | 0 |
| 337 | 0 |  | 1 | 36 | 29.3 | 88.5 | 83 | 290 | 57 | 219 | 138 | 6.4 | 24.6 | 119 | 74 | 5.1 | 0 | 0 | 0 | 0 | 0 | 0 |
| 338 | 1 | 0 | 0 | 49 | 24.6 | 74.5 | 99 | 200 | 58 | 134 | 51 | 5.3 | 37.1 | 131 | 81 | 5.5 | 0 | 0 | 0 | 1 | 0 | 0 |
| 339 | 0 |  | 1 | 61 | 21.3 | 78.0 | 97 | 200 | 61 | 116 | 48 | 5.1 | 20.4 | 151 | 93 | 5.3 | 0 | 0 | 0 | 1 | 0 | 0 |
| 340 | 0 |  | 0 | 56 | 19.4 | 56.0 | 75 | 214 | 90 | 105 | 27 | 6.1 | 34.4 | 129 | 82 | 5.4 | 0 | 0 | 0 | 1 | 0 | 0 |
| 341 | 1 | 0 | 1 | 51 | 24.4 | 84.0 | 93 | 214 | 61 | 130 | 128 | 6.6 | 44.6 | 153 | 96 | 5.2 | 0 | 0 | 0 | 1 | 0 | 0 |
| 342 | 1 | 0 | 1 | 58 | 26.1 | 83.0 | 95 | 141 | 40 | 80 | 127 | 8.2 | 30.8 | 121 | 76 | 5.4 | 0 | 0 | 0 | 1 | 0 | 0 |
| 343 | 0 |  | 0 | 40 | 20.0 | 66.0 | 89 | 182 | 49 | 126 | 82 | 4.5 | 14.1 | 100 | 64 | 5.5 | 0 | 0 | 1 | 0 | 0 | 0 |
| 344 | 0 |  | 1 | 52 | 30.1 | 99.0 | 95 | 169 | 51 | 114 | 45 | 6.9 | 33.8 | 138 | 96 | 5.3 | 1 | 0 | 0 | 1 | 0 | 0 |
| 345 | 1 | 0 | 0 | 57 | 27.2 | 80.0 | 125 | 220 | 47 | 130 | 240 | 5.4 | 17.5 | 122 | 70 | 5.9 | 1 | 1 | 1 | 0 | 1 | 1 |
| 346 | 1 | 0 | 0 | 56 | 21.4 | 75.0 | 96 | 169 | 57 | 102 | 61 | 4.3 | 20.7 | 126 | 80 | 6.1 | 0 | 0 | 0 | 1 | 1 | 0 |
| 347 | 1 | 0 | 0 | 51 | 25.5 | 76.0 | 110 | 190 | 44 | 125 | 148 | 5.0 | 22.0 | 102 | 62 | 5.5 | 0 | 0 | 1 | 0 | 1 | 0 |
| 348 | 0 |  | 0 | 44 | 25.3 | 74.0 | 89 | 172 | 57 | 98 | 70 | 4.1 | 28.6 | 101 | 46 | 5.2 | 0 | 0 | 0 | 0 | 0 | 0 |
| 349 | 0 |  | 0 | 43 | 19.0 | 68.5 | 76 | 229 | 94 | 112 | 88 | 5.4 | 18.7 | 113 | 70 | 4.7 | 0 | 0 | 0 | 0 | 0 | 0 |
| 350 | 0 |  | 0 | 60 | 22.8 | 71.0 | 94 | 217 | 41 | 141 | 112 | 5.1 | 27.6 | 103 | 58 | 5.2 | 0 | 0 | 1 | 0 | 0 | 0 |
| 351 | 0 |  | 0 | 61 | 26.9 | 78.5 | 100 | 253 | 59 | 163 | 97 | 6.0 | 26.4 | 140 | 80 | 5.7 | 0 | 0 | 0 | 1 | 1 | 0 |
| 352 | 1 | 1 | 1 | 63 | 22.9 | 85.0 | 115 | 198 | 56 | 134 | 88 | 7.0 | 41.5 | 116 | 72 | 5.6 | 0 | 0 | 0 | 0 | 1 | 0 |
| 353 | 1 | 0 | 0 | 55 | 22.2 | 75.0 | 93 | 145 | 47 | 84 | 118 | 4.3 | 14.7 | 116 | 73 | 5.9 | 0 | 0 | 1 | 1 | 1 | 1 |
| 354 | 0 |  | 1 | 49 | 26.4 | 86.0 | 85 | 181 | 28 | 112 | 172 | 7.4 | 34.0 | 128 | 77 | 5.3 | 0 | 1 | 1 | 1 | 0 | 1 |
| 355 | 0 |  | 0 | 51 | 19.5 | 65.0 | 66 | 201 | 76 | 115 | 35 | 4.3 | 17.3 | 115 | 55 | 5.0 | 0 | 0 | 0 | 0 | 0 | 0 |
| 356 | 1 | 1 | 1 | 56 | 26.5 | 88.0 | 109 | 241 | 30 | 173 | 217 | 6.1 | 16.0 | 112 | 75 | 6.2 | 0 | 1 | 1 | 0 | 1 | 1 |
| 357 | 0 |  | 0 | 49 | 20.8 | 70.0 | 96 | 271 | 85 | 166 | 49 | 3.7 | 33.9 | 128 | 79 | 5.4 | 0 | 0 | 0 | 0 | 0 | 0 |
| 358 | 0 |  | 1 | 54 | 21.5 | 76.5 | 89 | 156 | 44 | 88 | 115 | 5.2 | 32.1 | 112 | 70 | 5.5 | 0 | 0 | 0 | 0 | 0 | 0 |
| 359 | 1 | 1 | 0 | 43 | 21.2 | 74.0 | 84 | 156 | 70 | 77 | 38 | 5.6 | 18.9 | 126 | 80 | 4.8 | 0 | 0 | 0 | 0 | 0 | 0 |
| 360 | 1 | 1 | 0 | 36 | 25.4 | 86.0 | 96 | 195 | 60 | 115 | 79 | 4.8 | 23.1 | 112 | 67 | 5.7 | 1 | 0 | 0 | 0 | 0 | 0 |
| 361 | 1 | 1 | 1 | 61 | 23.0 | 87.0 | 116 | 211 | 60 | 116 | 239 | 7.1 | 27.7 | 149 | 88 | 5.3 | 0 | 1 | 0 | 1 | 1 | 1 |
| 362 | 0 |  | 1 | 45 | 25.5 | 80.0 | 92 | 192 | 55 | 110 | 217 | 5.3 | 21.9 | 117 | 75 | 5.5 | 0 | 1 | 0 | 0 | 0 | 0 |
| 363 | 0 |  | 0 | 60 | 20.2 | 65.0 | 93 | 256 | 76 | 142 | 118 | 4.5 | 14.6 | 102 | 65 | 4.8 | 0 | 0 | 0 | 0 | 0 | 0 |
| 364 | 0 |  | 1 | 42 | 26.2 | 82.5 | 82 | 197 | 43 | 145 | 149 | 7.3 | 43.0 | 127 | 84 | 5.2 | 0 | 0 | 0 | 0 | 0 | 0 |
| 365 | 0 |  | 0 | 55 | 27.8 | 90.0 | 168 | 146 | 39 | 79 | 164 | 6.1 | 30.9 | 127 | 71 | 6.9 | 1 | 1 | 1 | 1 | 1 | 1 |
| 366 | 1 | 1 | 1 | 50 | 25.2 | 80.0 | 96 | 169 | 48 | 89 | 270 | 5.7 | 30.8 | 115 | 73 | 6.4 | 0 | 1 | 0 | 0 | 1 | 0 |
| 367 | 0 |  | 0 | 39 | 16.7 | 62.0 | 83 | 144 | 51 | 90 | 57 | 4.2 | 15.6 | 114 | 65 | 5.5 | 0 | 0 | 0 | 0 | 0 | 0 |
| 368 | 0 |  | 0 | 57 | 20.0 | 69.0 | 104 | 195 | 69 | 104 | 56 | 3.5 | 31.7 | 141 | 75 | 5.7 | 0 | 0 | 0 | 1 | 1 | 0 |
| 369 | 1 | 1 | 1 | 62 | 26.5 | 94.0 | 209 | 240 | 30 | 133 | 369 | 7.5 | 25.5 | 143 | 82 | 6.9 | 1 | 1 | 1 | 1 | 1 | 1 |
| 370 | 1 | 0 | 0 | 49 | 19.4 | 64.0 | 81 | 228 | 71 | 136 | 66 | 4.2 | 31.7 | 118 | 65 | 5.0 | 0 | 0 | 0 | 0 | 0 | 0 |
| 371 | 0 |  | 0 | 53 | 20.3 | 64.5 | 85 | 222 | 51 | 139 | 153 | 5.2 | 33.5 | 113 | 77 | 5.3 | 0 | 1 | 0 | 0 | 0 | 0 |
| 372 | 1 | 0 | 0 | 48 | 23.0 | 67.5 | 84 | 198 | 71 | 110 | 47 | 5.4 | 22.7 | 162 | 90 | 5.3 | 0 | 0 | 0 | 1 | 0 | 0 |
| 373 | 0 |  | 1 | 63 | 24.4 | 84.0 | 101 | 215 | 35 | 152 | 181 | 6.8 | 26.8 | 136 | 85 | 5.7 | 0 | 1 | 1 | 1 | 1 | 1 |
| 374 | 1 | 0 | 1 | 53 | 26.6 | 90.0 | 147 | 218 | 65 | 134 | 150 | 5.7 | 25.4 | 151 | 94 | 7.5 | 1 | 1 | 0 | 1 | 1 | 1 |
| 375 | 0 |  | 1 | 54 | 24.0 | 84.0 | 102 | 149 | 48 | 77 | 98 | 5.4 | 31.2 | 120 | 79 | 6.2 | 0 | 0 | 0 | 0 | 1 | 0 |
| 376 | 1 | 1 | 1 | 60 | 35.2 | 116.0 | 91 | 158 | 62 | 91 | 59 | 7.3 | 42.4 | 137 | 76 | 5.4 | 1 | 0 | 0 | 1 | 0 | 0 |
| 377 | 1 | 1 | 0 | 47 | 22.0 | 74.0 | 72 | 186 | 77 | 101 | 33 | 4.1 | 18.7 | 115 | 81 | 4.8 | 0 | 0 | 0 | 0 | 0 | 0 |
| 378 | 1 | 1 | 0 | 50 | 22.9 | 80.0 | 104 | 236 | 66 | 146 | 64 | 4.3 | 18.3 | 137 | 79 | 5.5 | 1 | 0 | 0 | 1 | 1 | 1 |
| 379 | 0 |  | 0 | 35 | 19.5 | 67.0 | 91 | 245 | 68 | 162 | 74 | 3.6 | 30.1 | 154 | 98 | 5.3 | 0 | 0 | 0 | 1 | 0 | 0 |
| 380 | 0 |  | 1 | 50 | 23.6 | 80.0 | 84 | 116 | 38 | 57 | 112 | 6.2 | 32.5 | 127 | 78 | 5.2 | 0 | 0 | 1 | 0 | 0 | 0 |
| 381 | 0 |  | 1 | 37 | 22.7 | 74.0 | 72 | 243 | 45 | 165 | 251 | 9.1 | 25.2 | 102 | 70 | 5.5 | 0 | 1 | 0 | 0 | 0 | 0 |
| 382 | 0 |  | 0 | 56 | 19.3 | 64.0 | 66 | 270 | 74 | 171 | 75 | 6.0 | 33.1 | 107 | 75 | 5.0 | 0 | 0 | 0 | 0 | 0 | 0 |
| 383 | 0 |  | 0 | 54 | 25.6 | 82.0 | 98 | 248 | 38 | 139 | 301 | 5.0 | 29.9 | 110 | 79 | 5.5 | 1 | 1 | 1 | 1 | 0 | 1 |
| 384 | 0 |  | 1 | 40 | 25.5 | 77.5 | 97 | 171 | 57 | 102 | 85 | 6.3 | 19.9 | 160 | 104 | 5.4 | 0 | 0 | 0 | 1 | 0 | 0 |
| 385 | 0 |  | 0 | 51 | 27.4 | 93.5 | 85 | 212 | 62 | 133 | 64 | 5.8 | 20.3 | 112 | 77 | 5.3 | 1 | 0 | 0 | 0 | 0 | 0 |
| 386 | 0 |  | 0 | 51 | 18.0 | 70.0 | 82 | 162 | 43 | 108 | 62 | 4.4 | 16.1 | 118 | 67 | 5.2 | 0 | 0 | 1 | 0 | 0 | 0 |
| 387 | 0 |  | 0 | 57 | 23.4 | 83.5 | 104 | 205 | 47 | 138 | 106 | 3.8 | 36.7 | 130 | 78 | 5.4 | 1 | 0 | 1 | 1 | 1 | 1 |
| 388 | 0 |  | 0 | 63 | 29.9 | 90.0 | 130 | 191 | 43 | 128 | 87 | 5.6 | 19.9 | 126 | 63 | 5.9 | 1 | 0 | 1 | 0 | 1 | 1 |
| 389 | 0 |  | 1 | 42 | 25.3 | 88.0 | 85 | 216 | 49 | 152 | 59 | 8.1 | 42.3 | 138 | 83 | 5.2 | 0 | 0 | 0 | 1 | 0 | 0 |
| 390 | 1 | 1 | 1 | 47 | 24.7 | 87.5 | 91 | 193 | 35 | 135 | 111 | 5.3 | 22.1 | 122 | 81 | 3.9 | 0 | 0 | 1 | 0 | 0 | 0 |
| 391 | 0 |  | 0 | 60 | 23.6 | 83.0 | 103 | 189 | 60 | 100 | 112 | 6.2 | 29.0 | 132 | 83 | 5.6 | 1 | 0 | 0 | 1 | 1 | 1 |
| 392 | 0 |  | 1 | 60 | 33.5 | 102.0 | 113 | 225 | 57 | 142 | 120 | 5.9 | 45.0 | 146 | 87 | 5.6 | 1 | 0 | 0 | 1 | 1 | 1 |
| 393 | 1 | 1 | 1 | 40 | 21.6 | 84.5 | 89 | 149 | 41 | 94 | 87 | 6.5 | 22.3 | 130 | 86 | 5.4 | 0 | 0 | 0 | 1 | 0 | 0 |
| 394 | 0 |  | 1 | 48 | 25.6 | 82.0 | 91 | 229 | 33 | 157 | 163 | 7.9 | 22.6 | 109 | 77 | 5.1 | 0 | 1 | 1 | 0 | 0 | 0 |
| 395 | 0 |  | 0 | 49 | 28.2 | 86.5 | 95 | 205 | 49 | 127 | 146 | 4.2 | 19.8 | 134 | 74 | 5.4 | 1 | 0 | 1 | 1 | 0 | 1 |
| 396 | 0 |  | 0 | 41 | 20.5 | 75.0 | 101 | 216 | 58 | 148 | 85 | 4.4 | 24.8 | 110 | 70 | 5.5 | 0 | 0 | 0 | 0 | 1 | 0 |
| 397 | 0 |  | 1 | 57 | 26.4 | 89.0 | 107 | 159 | 45 | 96 | 140 | 6.5 | 30.7 | 118 | 79 | 6.2 | 0 | 0 | 0 | 1 | 1 | 0 |
| 398 | 0 |  | 1 | 55 | 25.7 | 88.0 | 131 | 165 | 47 | 91 | 203 | 6.4 | 43.8 | 126 | 79 | 6.5 | 0 | 1 | 0 | 0 | 1 | 0 |
| 399 | 0 |  | 1 | 50 | 20.3 | 79.0 | 100 | 156 | 32 | 114 | 53 | 7.5 | 33.0 | 111 | 78 | 5.8 | 0 | 0 | 1 | 0 | 1 | 0 |
| 400 | 1 | 0 | 1 | 55 | 27.2 | 90.0 | 121 | 162 | 35 | 107 | 118 | 4.4 | 26.7 | 140 | 83 | 6.5 | 1 | 0 | 1 | 1 | 1 | 1 |
| 401 | 1 | 0 | 1 | 55 | 24.7 | 86.0 | 109 | 297 | 53 | 222 | 78 | 7.5 | 37.2 | 139 | 81 | 6.1 | 0 | 0 | 0 | 1 | 1 | 0 |
| 402 | 1 | 1 | 1 | 54 | 25.6 | 87.0 | 92 | 221 | 39 | 149 | 210 | 5.9 | 55.5 | 156 | 107 | 5.6 | 0 | 1 | 1 | 1 | 0 | 1 |
| 403 | 0 |  | 0 | 48 | 22.9 | 79.0 | 95 | 235 | 43 | 181 | 82 | 4.3 | 20.7 | 105 | 66 | 5.0 | 0 | 0 | 1 | 0 | 0 | 0 |
| 404 | 0 |  | 0 | 64 | 25.9 | 78.0 | 108 | 157 | 52 | 79 | 105 | 4.1 | 19.6 | 147 | 81 | 5.9 | 0 | 0 | 0 | 1 | 1 | 0 |
| 405 | 0 |  | 1 | 44 | 26.3 | 87.0 | 92 | 216 | 38 | 128 | 231 | 6.1 | 13.3 | 114 | 75 | 5.0 | 0 | 1 | 1 | 0 | 0 | 0 |
| 406 | 0 |  | 1 | 41 | 29.4 | 94.0 | 86 | 138 | 39 | 76 | 133 | 7.5 | 26.5 | 110 | 74 | 5.2 | 1 | 0 | 1 | 1 | 0 | 1 |
| 407 | 1 | 1 | 1 | 54 | 22.0 | 77.0 | 93 | 227 | 64 | 148 | 81 | 5.4 | 33.3 | 120 | 75 | 5.4 | 0 | 0 | 0 | 0 | 0 | 0 |
| 408 | 0 |  | 1 | 64 | 26.5 | 86.0 | 107 | 237 | 44 | 181 | 74 | 6.0 | 34.3 | 153 | 92 | 5.4 | 0 | 0 | 0 | 1 | 1 | 0 |
| 409 | 1 | 0 | 1 | 51 | 23.5 | 83.0 | 95 | 180 | 26 | 128 | 165 | 5.4 | 41.1 | 122 | 83 | 5.9 | 0 | 1 | 1 | 0 | 1 | 1 |
| 410 | 0 |  | 1 | 45 | 29.2 | 92.0 | 96 | 199 | 28 | 101 | 366 | 7.4 | 19.5 | 102 | 65 | 5.1 | 1 | 1 | 1 | 0 | 0 | 1 |
| 411 | 1 | 0 | 0 | 44 | 20.0 | 68.0 | 77 | 183 | 64 | 109 | 25 | 3.9 | 24.5 | 126 | 76 | 4.8 | 0 | 0 | 0 | 0 | 0 | 0 |
| 412 | 1 | 1 | 1 | 40 | 25.0 | 82.5 | 84 | 173 | 38 | 125 | 82 | 6.5 | 43.4 | 108 | 72 | 5.2 | 0 | 0 | 1 | 1 | 0 | 0 |
| 413 | 1 | 0 | 1 | 41 | 27.7 | 83.0 | 90 | 221 | 48 | 151 | 66 | 7.1 | 30.4 | 123 | 79 | 4.9 | 0 | 0 | 0 | 0 | 0 | 0 |
| 414 | 1 | 1 | 0 | 53 | 18.7 | 63.5 | 87 | 239 | 65 | 160 | 79 | 3.7 | 18.7 | 113 | 63 | 5.5 | 0 | 0 | 0 | 0 | 0 | 0 |
| 415 | 0 |  | 0 | 49 | 27.6 | 86.0 | 113 | 236 | 47 | 171 | 140 | 5.5 | 23.4 | 152 | 95 | 5.8 | 1 | 0 | 1 | 1 | 1 | 1 |
| 416 | 1 | 1 | 1 | 63 | 25.6 | 85.0 | 149 | 179 | 38 | 105 | 187 | 4.2 | 30.5 | 133 | 75 | 6.6 | 0 | 1 | 1 | 1 | 1 | 1 |
| 417 | 0 |  | 0 | 38 | 20.9 | 65.0 | 86 | 188 | 80 | 85 | 29 | 4.4 | 23.5 | 94 | 59 | 5.1 | 0 | 0 | 0 | 1 | 0 | 0 |
| 418 | 1 | 1 | 1 | 37 | 19.3 | 67.0 | 84 | 263 | 67 | 177 | 112 | 6.5 | 28.9 | 117 | 70 | 5.4 | 0 | 0 | 0 | 0 | 0 | 0 |
| 419 | 0 |  | 0 | 60 | 22.0 | 79.0 | 100 | 178 | 58 | 106 | 59 | 5.6 | 18.3 | 121 | 66 | 5.6 | 0 | 0 | 0 | 0 | 1 | 0 |
| 420 | 1 | 1 | 1 | 59 | 27.9 | 98.0 | 88 | 216 | 45 | 142 | 163 | 6.4 | 36.7 | 141 | 72 | 5.2 | 1 | 1 | 0 | 1 | 0 | 1 |
| 421 | 1 | 1 | 1 | 62 | 23.2 | 77.0 | 131 | 128 | 23 | 66 | 252 | 7.9 | 30.6 | 114 | 76 | 6.5 | 0 | 1 | 1 | 1 | 1 | 1 |
| 422 | 0 |  | 0 | 51 | 20.7 | 69.0 | 85 | 219 | 76 | 124 | 74 | 4.0 | 11.2 | 97 | 64 | 5.0 | 0 | 0 | 0 | 1 | 0 | 0 |
| 423 | 1 | 1 | 0 | 54 | 30.6 | 82.0 | 140 | 159 | 54 | 86 | 52 | 5.8 | 15.8 | 128 | 74 | 6.5 | 1 | 0 | 0 | 0 | 1 | 0 |
| 424 | 0 |  | 1 | 39 | 24.6 | 87.5 | 95 | 183 | 79 | 100 | 109 | 5.8 | 26.9 | 120 | 77 | 4.9 | 0 | 0 | 0 | 0 | 0 | 0 |
| 425 | 1 | 1 | 1 | 50 | 24.3 | 88.0 | 85 | 134 | 46 | 76 | 46 | 8.2 | 50.6 | 116 | 75 | 5.2 | 0 | 0 | 0 | 0 | 0 | 0 |
| 426 | 1 | 1 | 0 | 49 | 25.6 | 87.0 | 118 | 207 | 33 | 128 | 319 | 6.6 | 15.8 | 102 | 65 | 5.5 | 1 | 1 | 1 | 0 | 1 | 1 |
| 427 | 0 |  | 0 | 41 | 21.1 | 69.5 | 96 | 162 | 42 | 97 | 94 | 4.6 | 24.6 | 109 | 68 | 5.3 | 0 | 0 | 1 | 0 | 0 | 0 |
| 428 | 1 | 0 | 0 | 54 | 20.0 | 67.0 | 100 | 223 | 103 | 90 | 61 | 4.0 | 19.0 | 109 | 70 | 5.5 | 0 | 0 | 0 | 0 | 1 | 0 |
| 429 | 0 |  | 0 | 34 | 23.8 | 77.0 | 91 | 195 | 45 | 116 | 107 | 5.3 | 19.2 | 113 | 78 | 5.5 | 0 | 0 | 1 | 0 | 0 | 0 |
| 430 | 0 |  | 0 | 57 | 20.6 | 66.0 | 80 | 195 | 111 | 71 | 37 | 4.4 | 21.8 | 105 | 67 | 5.2 | 0 | 0 | 0 | 0 | 0 | 0 |
| 431 | 1 | 0 | 1 | 32 | 35.3 | 104.0 | 99 | 268 | 39 | 144 | 454 | 6.8 | 26.0 | 120 | 70 | 5.5 | 1 | 1 | 1 | 0 | 0 | 1 |
| 432 | 0 |  | 0 | 37 | 22.7 | 78.0 | 99 | 143 | 50 | 78 | 43 | 5.2 | 30.7 | 95 | 69 | 5.3 | 0 | 0 | 0 | 1 | 0 | 0 |
| 433 | 1 | 1 | 0 | 47 | 22.6 | 67.5 | 89 | 177 | 57 | 102 | 50 | 4.9 | 14.4 | 123 | 63 | 5.5 | 0 | 0 | 0 | 0 | 0 | 0 |
| 434 | 0 |  | 1 | 40 | 28.2 | 91.0 | 89 | 209 | 36 | 142 | 161 | 8.2 | 24.4 | 112 | 79 | 5.4 | 1 | 1 | 1 | 0 | 0 | 1 |
| 435 | 0 |  | 0 | 55 | 26.0 | 80.5 | 81 | 265 | 71 | 160 | 83 | 4.5 | 38.1 | 115 | 64 | 5.4 | 1 | 0 | 0 | 0 | 0 | 0 |
| 436 | 1 | 0 | 0 | 53 | 21.7 | 72.0 | 87 | 191 | 60 | 117 | 59 | 5.9 | 28.1 | 125 | 79 | 5.6 | 0 | 0 | 0 | 0 | 0 | 0 |
| 437 | 1 | 1 | 1 | 47 | 30.1 | 111.5 | 95 | 177 | 29 | 98 | 320 | 8.1 | 29.0 | 117 | 76 | 5.6 | 1 | 1 | 1 | 1 | 0 | 1 |
| 438 | 0 |  | 0 | 38 | 27.6 | 87.0 | 100 | 208 | 60 | 128 | 132 | 4.0 | 29.6 | 117 | 76 | 5.3 | 1 | 0 | 0 | 0 | 1 | 0 |
| 439 | 0 |  | 1 | 55 | 18.9 | 69.5 | 94 | 173 | 54 | 99 | 96 | 5.1 | 35.3 | 104 | 66 | 4.9 | 0 | 0 | 0 | 0 | 0 | 0 |
| 440 | 0 |  | 1 | 33 | 34.0 | 113.0 | 88 | 152 | 36 | 97 | 139 | 8.3 | 19.1 | 131 | 87 | 4.9 | 1 | 0 | 1 | 1 | 0 | 1 |
| 441 | 0 |  | 0 | 40 | 19.7 | 66.0 | 80 | 206 | 55 | 142 | 62 | 5.0 | 15.6 | 109 | 76 | 5.1 | 0 | 0 | 0 | 0 | 0 | 0 |
| 442 | 0 |  | 0 | 49 | 29.7 | 85.0 | 85 | 288 | 54 | 199 | 155 | 5.9 | 20.2 | 128 | 69 | 5.4 | 1 | 1 | 0 | 0 | 0 | 0 |
| 443 | 0 |  | 1 | 54 | 21.8 | 75.0 | 88 | 289 | 72 | 188 | 127 | 4.2 | 33.4 | 138 | 82 | 5.5 | 0 | 0 | 0 | 1 | 0 | 0 |
| 444 | 0 |  | 1 | 53 | 25.4 | 88.0 | 87 | 195 | 41 | 123 | 165 | 5.2 | 37.8 | 130 | 87 | 5.2 | 0 | 1 | 0 | 1 | 0 | 0 |
| 445 | 1 | 0 | 0 | 42 | 20.4 | 67.0 | 83 | 194 | 70 | 113 | 68 | 5.5 | 30.4 | 108 | 67 | 5.4 | 0 | 0 | 0 | 0 | 0 | 0 |
| 446 | 1 | 1 | 0 | 39 | 21.5 | 79.5 | 97 | 209 | 63 | 126 | 82 | 4.8 | 27.0 | 118 | 73 | 4.8 | 0 | 0 | 0 | 0 | 0 | 0 |
| 447 | 1 | 0 | 1 | 45 | 25.8 | 89.0 | 122 | 180 | 32 | 126 | 107 | 7.8 | 34.5 | 146 | 96 | 5.7 | 0 | 0 | 1 | 1 | 1 | 1 |
| 448 | 0 |  | 0 | 55 | 19.3 | 67.0 | 84 | 191 | 63 | 105 | 55 | 4.9 | 24.7 | 104 | 68 | 5.3 | 0 | 0 | 0 | 0 | 0 | 0 |
| 449 | 0 |  | 1 | 61 | 27.4 | 92.0 | 214 | 228 | 51 | 146 | 158 | 7.8 | 40.5 | 128 | 88 | 10.2 | 1 | 1 | 0 | 1 | 1 | 1 |
| 450 | 1 | 1 | 0 | 58 | 29.1 | 92.0 | 101 | 207 | 46 | 131 | 182 | 6.6 | 29.8 | 112 | 67 | 5.5 | 1 | 1 | 1 | 0 | 1 | 1 |
| 451 | 0 |  | 1 | 63 | 22.4 | 80.5 | 90 | 242 | 34 | 168 | 176 | 6.6 | 33.0 | 135 | 77 | 5.5 | 0 | 1 | 1 | 1 | 0 | 1 |
| 452 | 1 | 1 | 1 | 48 | 30.0 | 96.0 | 90 | 210 | 59 | 134 | 68 | 8.1 | 23.0 | 135 | 85 | 5.4 | 1 | 0 | 0 | 1 | 0 | 0 |
| 453 | 0 |  | 1 | 55 | 25.1 | 84.0 | 89 | 209 | 47 | 147 | 75 | 5.3 | 34.5 | 114 | 66 | 5.7 | 0 | 0 | 0 | 0 | 0 | 0 |
| 454 | 1 | 1 | 1 | 60 | 26.7 | 94.0 | 95 | 238 | 40 | 174 | 154 | 10.4 | 30.0 | 140 | 89 | 5.6 | 1 | 1 | 0 | 1 | 0 | 1 |
| 455 | 0 |  | 0 | 48 | 19.0 | 62.0 | 76 | 144 | 67 | 59 | 66 | 4.0 | 37.2 | 100 | 63 | 5.1 | 0 | 0 | 0 | 0 | 0 | 0 |
| 456 | 0 |  | 0 | 53 | 22.0 | 71.5 | 88 | 181 | 67 | 94 | 60 | 4.6 | 24.3 | 125 | 68 | 5.1 | 0 | 0 | 0 | 1 | 0 | 0 |
| 457 | 0 |  | 1 | 60 | 25.4 | 81.0 | 87 | 163 | 62 | 97 | 31 | 5.8 | 28.3 | 124 | 81 | 5.1 | 0 | 0 | 0 | 0 | 0 | 0 |
| 458 | 0 |  | 0 | 63 | 28.4 | 85.5 | 100 | 192 | 54 | 126 | 124 | 6.3 | 27.9 | 172 | 87 | 5.8 | 1 | 0 | 0 | 1 | 1 | 1 |
| 459 | 0 |  | 1 | 48 | 24.1 | 79.0 | 89 | 206 | 44 | 145 | 68 | 7.6 | 27.6 | 141 | 95 | 5.5 | 0 | 0 | 0 | 1 | 0 | 0 |
| 460 | 1 | 1 | 1 | 35 | 28.6 | 91.0 | 93 | 201 | 34 | 131 | 113 | 6.6 | 47.7 | 114 | 64 | 5.7 | 1 | 0 | 1 | 0 | 0 | 0 |
| 461 | 0 |  | 1 | 62 | 23.8 | 79.0 | 98 | 246 | 38 | 184 | 95 | 6.2 | 35.3 | 118 | 78 | 5.2 | 0 | 0 | 1 | 0 | 0 | 0 |
| 462 | 0 |  | 1 | 37 | 31.5 | 95.0 | 95 | 167 | 30 | 112 | 152 | 8.6 | 22.5 | 144 | 77 | 5.4 | 1 | 1 | 1 | 1 | 0 | 1 |
| 463 | 1 | 0 | 1 | 58 | 25.3 | 87.5 | 83 | 242 | 43 | 168 | 88 | 6.5 | 34.5 | 139 | 81 | 5.2 | 0 | 0 | 0 | 1 | 0 | 0 |
| 464 | 0 |  | 0 | 48 | 23.7 | 80.0 | 119 | 182 | 42 | 132 | 70 | 5.4 | 12.1 | 108 | 72 | 6.2 | 1 | 0 | 1 | 0 | 1 | 1 |
| 465 | 0 |  | 0 | 47 | 34.3 | 99.0 | 86 | 262 | 58 | 180 | 102 | 5.1 | 23.8 | 143 | 93 | 5.0 | 1 | 0 | 0 | 1 | 0 | 0 |
| 466 | 0 |  | 0 | 42 | 24.1 | 77.5 | 85 | 198 | 60 | 126 | 66 | 5.0 | 22.7 | 107 | 74 | 4.9 | 0 | 0 | 0 | 0 | 0 | 0 |
| 467 | 1 | 1 | 1 | 35 | 19.8 | 72.0 | 92 | 162 | 40 | 111 | 78 | 5.4 | 35.6 | 123 | 77 | 5.0 | 0 | 0 | 0 | 0 | 0 | 0 |
| 468 | 1 | 0 | 0 | 41 | 22.2 | 74.0 | 92 | 201 | 53 | 132 | 89 | 6.7 | 35.3 | 96 | 64 | 5.2 | 0 | 0 | 0 | 1 | 0 | 0 |
| 469 | 0 |  | 1 | 41 | 22.7 | 80.5 | 92 | 245 | 49 | 133 | 241 | 7.2 | 21.3 | 128 | 70 | 5.5 | 0 | 1 | 0 | 0 | 0 | 0 |
| 470 | 0 |  | 1 | 49 | 25.2 | 83.0 | 159 | 135 | 40 | 78 | 132 | 6.0 | 37.8 | 127 | 88 | 7.1 | 0 | 0 | 0 | 1 | 1 | 0 |
| 471 | 1 | 1 | 1 | 39 | 25.5 | 81.0 | 85 | 191 | 38 | 136 | 137 | 6.1 | 31.6 | 112 | 71 | 5.0 | 0 | 0 | 1 | 0 | 0 | 0 |
| 472 | 1 | 1 | 1 | 57 | 22.5 | 84.0 | 84 | 192 | 80 | 102 | 49 | 5.1 | 35.4 | 137 | 86 | 5.3 | 0 | 0 | 0 | 1 | 0 | 0 |
| 473 | 1 | 0 | 1 | 36 | 26.6 | 89.0 | 74 | 251 | 58 | 156 | 301 | 5.4 | 30.7 | 125 | 76 | 5.4 | 0 | 1 | 0 | 0 | 0 | 0 |
| 474 | 1 | 0 | 0 | 55 | 20.0 | 63.0 | 120 | 262 | 38 | 197 | 96 | 6.3 | 31.0 | 129 | 92 | 5.6 | 0 | 0 | 1 | 1 | 1 | 1 |
| 475 | 1 | 1 | 0 | 36 | 22.0 | 70.0 | 101 | 149 | 36 | 102 | 88 | 5.6 | 29.9 | 94 | 65 | 5.3 | 0 | 0 | 1 | 1 | 1 | 1 |
| 476 | 1 | 0 | 0 | 58 | 22.4 | 81.0 | 82 | 232 | 67 | 149 | 73 | 5.8 | 26.9 | 136 | 79 | 5.5 | 1 | 0 | 0 | 1 | 0 | 0 |
| 477 | 1 | 0 | 1 | 63 | 24.4 | 87.0 | 100 | 178 | 37 | 128 | 97 | 6.5 | 28.1 | 148 | 73 | 5.3 | 0 | 0 | 1 | 1 | 1 | 1 |
| 478 | 0 |  | 1 | 34 | 26.7 | 91.0 | 81 | 166 | 46 | 118 | 52 | 6.5 | 20.5 | 109 | 73 | 5.3 | 1 | 0 | 0 | 0 | 0 | 0 |
| 479 | 1 | 0 | 0 | 30 | 19.6 | 68.0 | 89 | 150 | 73 | 59 | 45 | 3.8 | 30.7 | 105 | 72 | 4.7 | 0 | 0 | 0 | 0 | 0 | 0 |
| 480 | 0 |  | 0 | 56 | 23.1 | 70.0 | 98 | 189 | 55 | 115 | 56 | 5.0 | 26.1 | 122 | 79 | 5.4 | 0 | 0 | 0 | 0 | 0 | 0 |
| 481 | 1 | 0 | 0 | 58 | 27.4 | 88.0 | 171 | 245 | 59 | 157 | 152 | 5.8 | 37.4 | 160 | 86 | 7.1 | 1 | 1 | 0 | 1 | 1 | 1 |
| 482 | 1 | 1 | 1 | 32 | 23.4 | 83.0 | 91 | 212 | 35 | 148 | 157 | 5.5 | 15.8 | 111 | 65 | 5.6 | 0 | 1 | 1 | 0 | 0 | 0 |
| 483 | 0 |  | 0 | 34 | 21.8 | 71.0 | 91 | 214 | 44 | 151 | 101 | 5.0 | 18.2 | 110 | 66 | 5.1 | 0 | 0 | 1 | 0 | 0 | 0 |
| 484 | 0 |  | 1 | 50 | 20.6 | 87.0 | 143 | 171 | 45 | 106 | 91 | 6.2 | 19.1 | 103 | 73 | 7.1 | 0 | 0 | 0 | 0 | 1 | 0 |
| 485 | 0 |  | 0 | 50 | 21.4 | 68.0 | 90 | 221 | 61 | 142 | 29 | 4.8 | 23.1 | 116 | 65 | 5.8 | 0 | 0 | 0 | 0 | 1 | 0 |
| 486 | 0 |  | 1 | 38 | 29.5 | 97.0 | 89 | 158 | 36 | 117 | 84 | 7.8 | 16.7 | 137 | 73 | 5.0 | 1 | 0 | 1 | 1 | 0 | 1 |
| 487 | 1 | 1 | 1 | 59 | 25.8 | 92.0 | 122 | 106 | 44 | 44 | 137 | 8.8 | 36.1 | 126 | 91 | 6.1 | 1 | 0 | 0 | 1 | 1 | 1 |
| 488 | 1 | 0 | 1 | 40 | 23.8 | 92.0 | 84 | 187 | 45 | 133 | 53 | 7.2 | 24.7 | 126 | 79 | 5.6 | 1 | 0 | 0 | 0 | 0 | 0 |
| 489 | 0 |  | 0 | 33 | 20.2 | 81.0 | 91 | 165 | 71 | 80 | 55 | 4.4 | 20.6 | 104 | 68 | 4.9 | 1 | 0 | 0 | 0 | 0 | 0 |
| 490 | 1 | 1 | 1 | 47 | 26.9 | 92.0 | 107 | 176 | 44 | 85 | 446 | 6.6 | 37.1 | 119 | 76 | 5.2 | 1 | 1 | 0 | 1 | 1 | 1 |
| 491 | 0 |  | 0 | 42 | 26.9 | 87.5 | 84 | 206 | 48 | 134 | 99 | 4.8 | 25.1 | 125 | 77 | 5.3 | 1 | 0 | 1 | 0 | 0 | 0 |
| 492 | 1 | 0 | 1 | 61 | 25.2 | 82.0 | 102 | 200 | 39 | 126 | 165 | 6.3 | 24.5 | 122 | 78 | 5.6 | 0 | 1 | 1 | 0 | 1 | 1 |
| 493 | 1 | 1 | 1 | 46 | 34.6 | 110.0 | 111 | 133 | 29 | 57 | 349 | 7.9 | 19.1 | 142 | 84 | 5.7 | 1 | 1 | 1 | 1 | 1 | 1 |
| 494 | 0 |  | 0 | 57 | 22.6 | 70.0 | 81 | 247 | 63 | 176 | 55 | 5.5 | 32.5 | 139 | 73 | 5.6 | 0 | 0 | 0 | 1 | 0 | 0 |
| 495 | 0 |  | 0 | 32 | 20.7 | 69.5 | 74 | 141 | 51 | 82 | 40 | 5.5 | 16.5 | 91 | 56 | 5.5 | 0 | 0 | 0 | 1 | 0 | 0 |
| 496 | 0 |  | 0 | 47 | 21.5 | 64.0 | 88 | 201 | 72 | 119 | 46 | 4.3 | 37.5 | 103 | 60 | 5.0 | 0 | 0 | 0 | 0 | 0 | 0 |
| 497 | 0 |  | 0 | 63 | 26.4 | 82.0 | 90 | 156 | 45 | 117 | 65 | 6.7 | 31.2 | 119 | 68 | 5.3 | 1 | 0 | 1 | 0 | 0 | 0 |
| 498 | 1 | 0 | 1 | 54 | 37.3 | 118.5 | 109 | 162 | 20 | 98 | 315 | 4.7 | 22.7 | 123 | 76 | 6.5 | 1 | 1 | 1 | 1 | 1 | 1 |
| 499 | 0 |  | 1 | 48 | 29.4 | 96.5 | 85 | 172 | 42 | 115 | 89 | 5.2 | 20.3 | 142 | 91 | 5.2 | 1 | 0 | 0 | 1 | 0 | 0 |
| 500 | 0 |  | 0 | 35 | 19.8 | 65.0 | 80 | 179 | 46 | 125 | 34 | 4.1 | 15.3 | 106 | 63 | 5.3 | 0 | 0 | 1 | 0 | 0 | 0 |
| 501 | 0 |  | 0 | 34 | 17.8 | 68.5 | 85 | 126 | 66 | 38 | 74 | 4.5 | 18.4 | 90 | 61 | 4.8 | 0 | 0 | 0 | 1 | 0 | 0 |
| 502 | 0 |  | 0 | 60 | 18.6 | 70.0 | 81 | 196 | 74 | 98 | 51 | 4.9 | 27.9 | 132 | 78 | 5.1 | 0 | 0 | 0 | 1 | 0 | 0 |
| 503 | 1 | 1 | 1 | 47 | 33.9 | 89.0 | 93 | 221 | 50 | 143 | 156 | 6.8 | 17.3 | 102 | 70 | 4.9 | 0 | 1 | 0 | 0 | 0 | 0 |
| 504 | 1 | 0 | 0 | 44 | 23.3 | 74.0 | 82 | 197 | 47 | 123 | 188 | 4.2 | 38.7 | 113 | 63 | 5.3 | 0 | 1 | 1 | 0 | 0 | 0 |
| 505 | 0 |  | 1 | 36 | 24.9 | 83.0 | 87 | 191 | 41 | 137 | 88 | 7.5 | 32.4 | 134 | 79 | 5.4 | 0 | 0 | 0 | 1 | 0 | 0 |
| 506 | 0 |  | 1 | 42 | 23.4 | 87.5 | 95 | 231 | 42 | 156 | 275 | 6.7 | 20.9 | 112 | 78 | 5.7 | 0 | 1 | 0 | 0 | 0 | 0 |
| 507 | 0 |  | 0 | 52 | 29.2 | 86.0 | 94 | 157 | 46 | 104 | 71 | 5.6 | 31.7 | 130 | 79 | 5.5 | 1 | 0 | 1 | 1 | 0 | 1 |
| 508 | 1 | 1 | 1 | 38 | 19.8 | 75.5 | 99 | 197 | 41 | 136 | 155 | 6.6 | 41.9 | 97 | 71 | 4.8 | 0 | 1 | 0 | 1 | 0 | 0 |
| 509 | 0 |  | 1 | 48 | 26.3 | 90.0 | 88 | 140 | 40 | 78 | 119 | 6.8 | 32.2 | 122 | 81 | 5.2 | 1 | 0 | 0 | 1 | 0 | 0 |
| 510 | 0 |  | 1 | 35 | 22.9 | 79.0 | 97 | 211 | 38 | 147 | 155 | 5.7 | 33.4 | 122 | 83 | 5.0 | 0 | 1 | 1 | 0 | 0 | 0 |
| 511 | 1 | 0 | 0 | 40 | 24.0 | 72.0 | 88 | 143 | 49 | 76 | 93 | 4.3 | 19.0 | 110 | 71 | 5.9 | 0 | 0 | 1 | 0 | 1 | 0 |
| 512 | 0 |  | 1 | 50 | 27.2 | 88.0 | 96 | 209 | 26 | 145 | 231 | 5.8 | 34.3 | 135 | 92 | 5.4 | 0 | 1 | 1 | 1 | 0 | 1 |
| 513 | 0 |  | 1 | 41 | 24.1 | 84.0 | 93 | 165 | 29 | 93 | 177 | 9.1 | 42.2 | 120 | 84 | 5.7 | 0 | 1 | 1 | 0 | 0 | 0 |
| 514 | 0 |  | 1 | 40 | 27.3 | 89.0 | 106 | 212 | 35 | 109 | 324 | 6.8 | 32.2 | 123 | 84 | 5.4 | 0 | 1 | 1 | 0 | 1 | 1 |
| 515 | 1 | 1 | 1 | 51 | 24.9 | 79.0 | 105 | 227 | 68 | 123 | 151 | 8.3 | 36.3 | 134 | 89 | 5.7 | 0 | 1 | 0 | 1 | 1 | 1 |
| 516 | 1 | 1 | 1 | 57 | 21.3 | 79.0 | 81 | 226 | 52 | 159 | 83 | 7.1 | 27.6 | 97 | 62 | 6.0 | 0 | 0 | 0 | 1 | 1 | 0 |
| 517 | 1 | 1 | 1 | 58 | 29.3 | 105.0 | 111 | 271 | 44 | 192 | 144 | 7.2 | 23.1 | 106 | 58 | 5.4 | 1 | 0 | 0 | 0 | 1 | 0 |
| 518 | 0 |  | 0 | 49 | 28.6 | 84.5 | 97 | 245 | 47 | 165 | 142 | 5.3 | 22.6 | 131 | 68 | 5.5 | 1 | 0 | 1 | 1 | 0 | 1 |
| 519 | 0 |  | 1 | 50 | 28.6 | 86.0 | 87 | 205 | 27 | 150 | 203 | 7.7 | 28.9 | 125 | 76 | 4.1 | 0 | 1 | 1 | 0 | 0 | 0 |
| 520 | 1 | 1 | 0 | 55 | 26.5 | 78.0 | 123 | 227 | 53 | 159 | 80 | 5.0 | 19.9 | 181 | 91 | 5.6 | 0 | 0 | 0 | 1 | 1 | 0 |
| 521 | 1 | 1 | 0 | 63 | 20.4 | 68.0 | 132 | 168 | 60 | 87 | 77 | 5.4 | 44.3 | 123 | 60 | 6.4 | 0 | 0 | 0 | 0 | 1 | 0 |
| 522 | 0 |  | 1 | 38 | 27.8 | 98.0 | 82 | 157 | 33 | 108 | 103 | 6.3 | 18.8 | 128 | 85 | 5.3 | 1 | 0 | 1 | 1 | 1 | 1 |
| 523 | 1 | 0 | 0 | 51 | 29.8 | 90.0 | 105 | 225 | 50 | 133 | 167 | 6.9 | 27.0 | 139 | 91 | 5.6 | 1 | 1 | 0 | 1 | 1 | 1 |
| 524 | 0 |  | 0 | 38 | 23.2 | 75.5 | 88 | 202 | 68 | 121 | 58 | 4.3 | 20.7 | 111 | 70 | 4.6 | 0 | 0 | 0 | 0 | 0 | 0 |
| 525 | 0 |  | 1 | 60 | 26.3 | 85.0 | 102 | 185 | 51 | 121 | 84 | 5.4 | 30.5 | 121 | 77 | 5.4 | 0 | 0 | 0 | 0 | 1 | 0 |
| 526 | 0 |  | 1 | 31 | 27.8 | 85.0 | 92 | 229 | 41 | 158 | 233 | 7.5 | 32.4 | 133 | 80 | 5.2 | 0 | 1 | 0 | 1 | 0 | 0 |
| 527 | 0 |  | 1 | 48 | 26.3 | 84.0 | 107 | 178 | 32 | 123 | 127 | 7.1 | 36.1 | 125 | 77 | 5.8 | 0 | 0 | 1 | 0 | 1 | 0 |
| 528 | 0 |  | 0 | 30 | 19.9 | 72.0 | 80 | 182 | 66 | 100 | 56 | 4.4 | 15.2 | 106 | 61 | 5.3 | 0 | 0 | 0 | 0 | 0 | 0 |
| 529 | 1 | 1 | 1 | 59 | 29.5 | 94.0 | 92 | 254 | 44 | 173 | 258 | 10.1 | 24.9 | 127 | 83 | 5.2 | 1 | 1 | 0 | 0 | 0 | 0 |
| 530 | 0 |  | 0 | 37 | 21.1 | 74.0 | 64 | 178 | 50 | 108 | 67 | 7.4 | 35.2 | 128 | 82 | 5.2 | 0 | 0 | 0 | 0 | 0 | 0 |
| 531 | 0 |  | 0 | 59 | 25.1 | 82.0 | 100 | 190 | 52 | 125 | 76 | 5.7 | 45.4 | 133 | 83 | 5.7 | 1 | 0 | 0 | 1 | 1 | 1 |
| 532 | 1 | 1 | 1 | 40 | 32.2 | 101.0 | 176 | 176 | 31 | 105 | 250 | 5.9 | 25.0 | 154 | 102 | 7.8 | 1 | 1 | 1 | 1 | 1 | 1 |
| 533 | 0 |  | 1 | 36 | 33.9 | 110.0 | 90 | 206 | 36 | 150 | 190 | 8.9 | 31.4 | 133 | 94 | 5.2 | 1 | 1 | 1 | 1 | 0 | 1 |
| 534 | 0 |  | 1 | 32 | 30.4 | 96.0 | 83 | 240 | 34 | 183 | 89 | 8.1 | 30.5 | 108 | 67 | 5.6 | 1 | 0 | 1 | 0 | 0 | 0 |
| 535 | 1 | 0 | 0 | 50 | 21.7 | 72.0 | 89 | 200 | 54 | 146 | 51 | 4.5 | 9.4 | 96 | 57 | 5.6 | 0 | 0 | 0 | 1 | 0 | 0 |
| 536 | 0 |  | 1 | 51 | 23.7 | 78.0 | 88 | 244 | 69 | 161 | 50 | 5.3 | 27.5 | 117 | 66 | 6.0 | 0 | 0 | 0 | 0 | 1 | 0 |
| 537 | 1 | 1 | 1 | 50 | 25.8 | 87.5 | 100 | 225 | 47 | 116 | 237 | 7.2 | 27.6 | 130 | 90 | 5.1 | 0 | 1 | 0 | 1 | 1 | 1 |
| 538 | 0 |  | 0 | 56 | 20.1 | 69.0 | 92 | 201 | 51 | 133 | 59 | 5.1 | 47.8 | 114 | 69 | 5.2 | 0 | 0 | 0 | 0 | 0 | 0 |
| 539 | 0 |  | 1 | 49 | 23.4 | 92.0 | 95 | 208 | 48 | 149 | 82 | 7.2 | 12.1 | 113 | 66 | 5.0 | 1 | 0 | 0 | 0 | 0 | 0 |
| 540 | 0 |  | 0 | 43 | 29.7 | 91.0 | 92 | 162 | 36 | 103 | 134 | 5.2 | 20.8 | 129 | 75 | 5.2 | 1 | 0 | 1 | 0 | 0 | 0 |
| 541 | 0 |  | 1 | 32 | 23.4 | 73.0 | 102 | 159 | 46 | 103 | 75 | 7.1 | 24.9 | 125 | 64 | 5.0 | 0 | 0 | 0 | 0 | 1 | 0 |
| 542 | 0 |  | 0 | 62 | 27.5 | 80.0 | 91 | 265 | 59 | 162 | 142 | 5.5 | 38.5 | 111 | 61 | 6.0 | 1 | 0 | 0 | 1 | 1 | 1 |
| 543 | 0 |  | 0 | 39 | 27.1 | 84.0 | 84 | 214 | 58 | 144 | 70 | 5.4 | 21.9 | 111 | 68 | 5.2 | 1 | 0 | 0 | 0 | 0 | 0 |
| 544 | 0 |  | 1 | 53 | 24.6 | 81.5 | 99 | 233 | 46 | 156 | 185 | 5.9 | 18.0 | 136 | 91 | 5.5 | 0 | 1 | 0 | 1 | 0 | 0 |
| 545 | 0 |  | 0 | 33 | 18.8 | 70.0 | 89 | 173 | 42 | 113 | 72 | 4.8 | 16.9 | 113 | 72 | 5.1 | 0 | 0 | 1 | 0 | 0 | 0 |
| 546 | 1 | 0 | 1 | 62 | 28.6 | 97.0 | 92 | 200 | 32 | 152 | 112 | 6.1 | 36.4 | 100 | 71 | 5.3 | 1 | 0 | 1 | 0 | 0 | 0 |
| 547 | 1 | 1 | 1 | 39 | 36.9 | 113.0 | 124 | 240 | 28 | 163 | 276 | 8.6 | 26.4 | 129 | 92 | 6.2 | 1 | 1 | 1 | 1 | 1 | 1 |
| 548 | 1 | 1 | 1 | 47 | 27.7 | 87.0 | 105 | 233 | 45 | 151 | 141 | 8.0 | 21.2 | 128 | 83 | 5.4 | 0 | 0 | 0 | 0 | 1 | 0 |
| 549 | 1 | 0 | 1 | 50 | 22.7 | 70.0 | 90 | 198 | 73 | 107 | 36 | 4.4 | 19.4 | 119 | 70 | 5.3 | 0 | 0 | 0 | 0 | 0 | 0 |
| 550 | 1 | 1 | 0 | 35 | 21.6 | 77.0 | 86 | 195 | 57 | 121 | 62 | 4.9 | 16.0 | 109 | 67 | 4.9 | 0 | 0 | 0 | 0 | 0 | 0 |
| 551 | 1 | 1 | 0 | 60 | 23.4 | 72.0 | 109 | 246 | 50 | 164 | 204 | 5.1 | 16.6 | 139 | 90 | 6.0 | 0 | 1 | 0 | 1 | 1 | 1 |
| 552 | 1 | 1 | 1 | 41 | 24.1 | 93.0 | 101 | 171 | 35 | 99 | 207 | 5.2 | 35.3 | 122 | 81 | 5.2 | 1 | 1 | 1 | 0 | 1 | 1 |
| 553 | 0 |  | 0 | 40 | 21.5 | 68.0 | 89 | 207 | 63 | 131 | 57 | 5.2 | 27.1 | 110 | 71 | 5.4 | 0 | 0 | 0 | 0 | 0 | 0 |
| 554 | 0 |  | 1 | 40 | 22.7 | 79.0 | 97 | 243 | 47 | 179 | 126 | 7.2 | 24.6 | 113 | 77 | 5.2 | 0 | 0 | 0 | 0 | 0 | 0 |
| 555 | 0 |  | 0 | 63 | 22.4 | 76.5 | 93 | 208 | 50 | 143 | 64 | 5.8 | 28.9 | 138 | 72 | 5.2 | 0 | 0 | 0 | 1 | 0 | 0 |
| 556 | 0 |  | 1 | 36 | 21.6 | 69.0 | 86 | 122 | 50 | 62 | 38 | 5.9 | 14.7 | 119 | 77 | 5.1 | 0 | 0 | 0 | 0 | 0 | 0 |
| 557 | 1 | 0 | 1 | 46 | 33.3 | 110.0 | 89 | 168 | 42 | 107 | 81 | 4.5 | 41.0 | 124 | 71 | 5.6 | 1 | 0 | 0 | 0 | 0 | 0 |
| 558 | 0 |  | 1 | 36 | 24.5 | 77.0 | 100 | 228 | 48 | 161 | 72 | 6.5 | 29.2 | 112 | 73 | 5.3 | 0 | 0 | 0 | 0 | 1 | 0 |
| 559 | 0 |  | 1 | 33 | 29.9 | 99.0 | 96 | 175 | 33 | 138 | 62 | 7.5 | 16.9 | 119 | 73 | 5.2 | 1 | 0 | 1 | 0 | 0 | 0 |
| 560 | 0 |  | 1 | 55 | 23.5 | 87.0 | 73 | 198 | 44 | 144 | 125 | 8.0 | 29.4 | 126 | 74 | 5.4 | 0 | 0 | 0 | 0 | 0 | 0 |
| 561 | 0 |  | 1 | 42 | 24.4 | 75.0 | 90 | 242 | 54 | 176 | 112 | 5.5 | 29.3 | 108 | 70 | 5.3 | 0 | 0 | 0 | 0 | 0 | 0 |
| 562 | 1 | 1 | 1 | 48 | 23.3 | 85.0 | 122 | 187 | 41 | 118 | 178 | 11.1 | 30.1 | 156 | 99 | 5.6 | 0 | 1 | 0 | 1 | 1 | 1 |
| 563 | 1 | 1 | 1 | 53 | 25.0 | 91.0 | 101 | 149 | 35 | 95 | 176 | 8.3 | 20.7 | 111 | 75 | 6.1 | 1 | 1 | 1 | 1 | 1 | 1 |
| 564 | 1 | 0 | 1 | 56 | 29.4 | 88.0 | 91 | 177 | 67 | 97 | 56 | 6.5 | 15.9 | 127 | 81 | 5.8 | 0 | 0 | 0 | 0 | 1 | 0 |
| 565 | 0 |  | 1 | 34 | 27.6 | 93.0 | 97 | 277 | 36 | 178 | 192 | 8.3 | 24.2 | 122 | 79 | 5.0 | 1 | 1 | 1 | 0 | 0 | 1 |
| 566 | 0 |  | 0 | 37 | 20.4 | 70.0 | 87 | 170 | 67 | 88 | 36 | 4.9 | 17.4 | 115 | 73 | 5.0 | 0 | 0 | 0 | 0 | 0 | 0 |
| 567 | 1 | 0 | 1 | 41 | 35.5 | 108.0 | 102 | 246 | 27 | 162 | 304 | 8.7 | 38.3 | 126 | 63 | 5.5 | 1 | 1 | 1 | 0 | 1 | 1 |
| 568 | 0 |  | 1 | 33 | 31.8 | 97.0 | 94 | 230 | 41 | 159 | 189 | 7.5 | 20.1 | 118 | 81 | 5.4 | 1 | 1 | 0 | 0 | 0 | 0 |
| 569 | 0 |  | 1 | 46 | 29.4 | 97.0 | 92 | 197 | 43 | 125 | 157 | 7.1 | 23.1 | 118 | 77 | 4.9 | 1 | 1 | 0 | 0 | 0 | 0 |
| 570 | 1 | 0 | 0 | 58 | 20.6 | 70.0 | 83 | 228 | 70 | 133 | 90 | 5.1 | 19.8 | 129 | 89 | 5.6 | 0 | 0 | 0 | 1 | 0 | 0 |
| 571 | 0 |  | 0 | 40 | 21.2 | 62.0 | 76 | 334 | 111 | 201 | 101 | 3.8 | 21.1 | 93 | 68 | 4.8 | 0 | 0 | 0 | 1 | 0 | 0 |
| 572 | 0 |  | 0 | 54 | 26.7 | 94.0 | 94 | 196 | 47 | 135 | 118 | 4.5 | 23.9 | 111 | 74 | 5.4 | 1 | 0 | 1 | 0 | 0 | 0 |
| 573 | 1 | 1 | 1 | 59 | 24.3 | 87.0 | 89 | 136 | 46 | 58 | 182 | 3.7 | 22.9 | 116 | 67 | 5.5 | 0 | 1 | 0 | 1 | 0 | 0 |
| 574 | 1 | 0 | 0 | 51 | 24.4 | 80.0 | 89 | 219 | 52 | 150 | 98 | 3.8 | 16.1 | 110 | 72 | 5.4 | 1 | 0 | 0 | 0 | 0 | 0 |
| 575 | 0 |  | 0 | 41 | 21.9 | 70.5 | 90 | 197 | 72 | 108 | 70 | 4.2 | 10.7 | 90 | 61 | 5.7 | 0 | 0 | 0 | 1 | 0 | 0 |
| 576 | 1 | 0 | 0 | 35 | 19.0 | 64.0 | 87 | 228 | 76 | 127 | 55 | 5.7 | 15.3 | 98 | 71 | 4.9 | 0 | 0 | 0 | 1 | 0 | 0 |
| 577 | 0 |  | 0 | 37 | 22.1 | 76.0 | 86 | 206 | 66 | 129 | 58 | 4.8 | 10.0 | 93 | 65 | 5.0 | 0 | 0 | 0 | 1 | 0 | 0 |
| 578 | 1 | 0 | 0 | 33 | 22.4 | 70.0 | 88 | 217 | 78 | 127 | 62 | 4.5 | 26.6 | 97 | 62 | 5.0 | 0 | 0 | 0 | 1 | 0 | 0 |
| 579 | 0 |  | 0 | 36 | 20.9 | 64.0 | 88 | 133 | 46 | 69 | 65 | 4.0 | 17.0 | 114 | 73 | 5.1 | 0 | 0 | 1 | 0 | 0 | 0 |
| 580 | 0 |  | 1 | 56 | 24.9 | 86.5 | 101 | 295 | 46 | 158 | 473 | 6.3 | 32.3 | 175 | 93 | 5.8 | 0 | 1 | 0 | 1 | 1 | 1 |
| 581 | 1 | 0 | 1 | 51 | 25.5 | 83.0 | 90 | 201 | 54 | 130 | 98 | 6.0 | 10.4 | 133 | 90 | 5.2 | 0 | 0 | 0 | 1 | 0 | 0 |
| 582 | 0 |  | 0 | 37 | 19.3 | 64.0 | 89 | 171 | 44 | 103 | 86 | 5.6 | 34.7 | 106 | 74 | 5.3 | 0 | 0 | 1 | 1 | 0 | 0 |
| 583 | 0 |  | 1 | 47 | 20.3 | 80.0 | 95 | 197 | 61 | 113 | 110 | 5.6 | 40.7 | 119 | 78 | 5.4 | 0 | 0 | 0 | 0 | 0 | 0 |
| 584 | 0 |  | 1 | 48 | 20.4 | 72.0 | 77 | 193 | 45 | 132 | 51 | 6.4 | 34.9 | 108 | 67 | 5.2 | 0 | 0 | 0 | 0 | 0 | 0 |
| 585 | 1 | 0 | 0 | 32 | 18.6 | 69.0 | 76 | 205 | 61 | 122 | 61 | 5.1 | 24.5 | 108 | 69 | 5.1 | 0 | 0 | 0 | 0 | 0 | 0 |
| 586 | 0 |  | 1 | 37 | 26.9 | 93.0 | 92 | 234 | 48 | 174 | 79 | 8.6 | 24.9 | 129 | 89 | 5.5 | 1 | 0 | 0 | 1 | 0 | 0 |
| 587 | 0 |  | 0 | 45 | 21.0 | 69.0 | 99 | 255 | 46 | 182 | 111 | 4.2 | 22.1 | 102 | 70 | 5.6 | 0 | 0 | 1 | 0 | 0 | 0 |
| 588 | 1 | 0 | 1 | 63 | 23.9 | 85.5 | 113 | 146 | 44 | 81 | 36 | 6.5 | 29.4 | 150 | 100 | 5.8 | 0 | 0 | 0 | 1 | 1 | 0 |
| 589 | 1 | 0 | 0 | 39 | 19.1 | 62.0 | 85 | 246 | 69 | 157 | 62 | 3.7 | 24.2 | 88 | 55 | 4.8 | 0 | 0 | 0 | 1 | 0 | 0 |
| 590 | 1 | 1 | 0 | 60 | 25.9 | 90.0 | 105 | 227 | 51 | 162 | 71 | 3.8 | 30.6 | 138 | 89 | 5.8 | 1 | 0 | 0 | 1 | 1 | 1 |
| 591 | 1 | 0 | 1 | 41 | 27.0 | 93.0 | 95 | 240 | 42 | 169 | 193 | 6.8 | 15.6 | 114 | 72 | 5.1 | 1 | 1 | 0 | 0 | 0 | 0 |
| 592 | 0 |  | 1 | 60 | 23.2 | 76.0 | 98 | 212 | 63 | 138 | 73 | 5.7 | 34.8 | 123 | 77 | 5.2 | 0 | 0 | 0 | 0 | 0 | 0 |
| 593 | 0 |  | 1 | 45 | 21.4 | 75.0 | 99 | 221 | 40 | 167 | 114 | 6.0 | 27.9 | 120 | 74 | 4.0 | 0 | 0 | 0 | 0 | 0 | 0 |
| 594 | 0 |  | 0 | 39 | 21.4 | 64.0 | 87 | 217 | 41 | 171 | 62 | 5.5 | 19.7 | 135 | 80 | 4.8 | 0 | 0 | 1 | 1 | 0 | 0 |
| 595 | 1 | 0 | 1 | 53 | 21.1 | 72.5 | 83 | 197 | 47 | 149 | 60 | 5.4 | 46.3 | 118 | 72 | 5.1 | 0 | 0 | 0 | 0 | 0 | 0 |
| 596 | 0 |  | 1 | 40 | 25.2 | 80.0 | 83 | 186 | 64 | 101 | 103 | 7.2 | 24.3 | 120 | 75 | 4.7 | 0 | 0 | 0 | 0 | 0 | 0 |
| 597 | 0 |  | 0 | 40 | 22.0 | 72.5 | 93 | 159 | 50 | 96 | 61 | 3.9 | 21.3 | 101 | 56 | 5.5 | 0 | 0 | 0 | 0 | 0 | 0 |
| 598 | 1 | 1 | 1 | 55 | 22.3 | 84.0 | 97 | 202 | 48 | 143 | 66 | 5.9 | 30.3 | 116 | 72 | 6.0 | 0 | 0 | 0 | 0 | 1 | 0 |
| 599 | 1 | 0 | 1 | 45 | 24.3 | 86.0 | 269 | 236 | 42 | 159 | 170 | 4.2 | 25.0 | 105 | 64 | 11.4 | 0 | 1 | 0 | 0 | 1 | 0 |
| 600 | 1 | 0 | 0 | 40 | 20.3 | 64.0 | 83 | 223 | 51 | 136 | 85 | 5.5 | 17.5 | 103 | 63 | 5.1 | 0 | 0 | 0 | 0 | 0 | 0 |
| 601 | 1 | 1 | 1 | 46 | 23.6 | 80.0 | 99 | 260 | 53 | 161 | 227 | 7.7 | 24.0 | 146 | 86 | 5.7 | 0 | 1 | 0 | 1 | 0 | 0 |
| 602 | 1 | 0 | 1 | 62 | 21.4 | 78.0 | 149 | 162 | 58 | 89 | 44 | 3.9 | 24.0 | 142 | 85 | 6.5 | 0 | 0 | 0 | 1 | 1 | 0 |
| 603 | 0 |  | 0 | 58 | 20.0 | 67.0 | 94 | 228 | 81 | 127 | 47 | 6.1 | 35.5 | 123 | 68 | 5.7 | 0 | 0 | 0 | 0 | 0 | 0 |
| 604 | 0 |  | 0 | 47 | 21.3 | 75.5 | 89 | 267 | 71 | 173 | 73 | 4.0 | 20.6 | 119 | 73 | 4.7 | 0 | 0 | 0 | 0 | 0 | 0 |
| 605 | 0 |  | 0 | 34 | 25.8 | 82.5 | 82 | 286 | 62 | 206 | 51 | 5.3 | 15.6 | 117 | 69 | 5.4 | 1 | 0 | 0 | 0 | 0 | 0 |
| 606 | 0 |  | 0 | 40 | 23.1 | 81.0 | 90 | 171 | 62 | 89 | 104 | 6.5 | 33.5 | 115 | 62 | 4.9 | 1 | 0 | 0 | 0 | 0 | 0 |
| 607 | 1 | 1 | 1 | 39 | 27.8 | 84.0 | 88 | 177 | 38 | 108 | 191 | 7.9 | 33.7 | 115 | 74 | 5.2 | 0 | 1 | 1 | 0 | 0 | 0 |
| 608 | 1 | 1 | 1 | 48 | 30.9 | 98.0 | 107 | 151 | 34 | 90 | 156 | 6.0 | 21.9 | 116 | 82 | 6.5 | 1 | 1 | 1 | 0 | 1 | 1 |
| 609 | 0 |  | 0 | 45 | 21.5 | 69.5 | 98 | 184 | 75 | 95 | 74 | 3.3 | 20.7 | 128 | 76 | 5.2 | 0 | 0 | 0 | 0 | 0 | 0 |
| 610 | 0 |  | 0 | 40 | 18.9 | 72.5 | 92 | 229 | 84 | 127 | 41 | 3.6 | 17.2 | 93 | 67 | 4.8 | 0 | 0 | 0 | 1 | 0 | 0 |
| 611 | 0 |  | 0 | 41 | 21.6 | 80.0 | 89 | 158 | 54 | 91 | 93 | 5.1 | 25.1 | 124 | 79 | 5.7 | 1 | 0 | 0 | 1 | 0 | 0 |
| 612 | 0 |  | 0 | 30 | 18.7 | 73.5 | 66 | 198 | 50 | 150 | 43 | 5.5 | 8.9 | 103 | 67 | 4.9 | 0 | 0 | 0 | 0 | 0 | 0 |
| 613 | 0 |  | 1 | 50 | 29.9 | 93.0 | 99 | 230 | 53 | 155 | 99 | 7.1 | 39.5 | 112 | 81 | 5.2 | 1 | 0 | 0 | 0 | 0 | 0 |
| 614 | 0 |  | 1 | 62 | 23.9 | 81.0 | 119 | 189 | 42 | 118 | 165 | 5.2 | 26.1 | 114 | 74 | 6.8 | 0 | 1 | 0 | 0 | 1 | 0 |
| 615 | 0 |  | 1 | 38 | 25.3 | 82.0 | 103 | 216 | 42 | 136 | 152 | 8.4 | 27.7 | 112 | 63 | 5.2 | 0 | 1 | 0 | 0 | 1 | 0 |
| 616 | 0 |  | 0 | 48 | 18.7 | 66.5 | 92 | 166 | 76 | 75 | 50 | 4.0 | 34.7 | 110 | 66 | 5.1 | 0 | 0 | 0 | 0 | 0 | 0 |
| 617 | 1 | 0 | 1 | 38 | 25.4 | 87.5 | 92 | 171 | 38 | 101 | 176 | 6.9 | 33.4 | 140 | 86 | 5.2 | 0 | 1 | 1 | 1 | 0 | 1 |
| 618 | 0 |  | 1 | 56 | 30.4 | 96.0 | 198 | 185 | 33 | 81 | 449 | 8.7 | 17.7 | 131 | 81 | 9.3 | 1 | 1 | 1 | 1 | 1 | 1 |
| 619 | 0 |  | 0 | 51 | 27.3 | 84.0 | 128 | 195 | 36 | 134 | 149 | 5.1 | 15.2 | 138 | 86 | 6.2 | 1 | 0 | 1 | 1 | 1 | 1 |
| 620 | 1 | 0 | 0 | 45 | 19.9 | 65.0 | 96 | 177 | 55 | 101 | 126 | 4.1 | 23.8 | 114 | 81 | 4.7 | 0 | 0 | 0 | 1 | 0 | 0 |
| 621 | 1 | 0 | 1 | 40 | 28.2 | 85.0 | 133 | 196 | 30 | 149 | 105 | 6.8 | 38.1 | 107 | 76 | 6.8 | 0 | 0 | 1 | 0 | 1 | 0 |
| 622 | 0 |  | 1 | 36 | 22.9 | 85.0 | 99 | 181 | 45 | 121 | 43 | 6.0 | 29.9 | 124 | 76 | 4.2 | 0 | 0 | 0 | 0 | 0 | 0 |
| 623 | 1 | 0 | 1 | 38 | 23.6 | 73.0 | 104 | 201 | 70 | 113 | 49 | 7.3 | 49.7 | 128 | 79 | 5.6 | 0 | 0 | 0 | 0 | 1 | 0 |
| 624 | 1 | 0 | 1 | 60 | 25.9 | 89.0 | 116 | 174 | 37 | 110 | 161 | 6.2 | 31.9 | 135 | 102 | 6.3 | 0 | 1 | 1 | 1 | 1 | 1 |
| 625 | 1 | 1 | 1 | 36 | 25.7 | 80.0 | 98 | 192 | 35 | 141 | 106 | 6.0 | 16.6 | 120 | 76 | 5.3 | 0 | 0 | 1 | 0 | 0 | 0 |
| 626 | 0 |  | 1 | 59 | 26.2 | 85.0 | 95 | 185 | 46 | 116 | 144 | 4.8 | 24.8 | 121 | 86 | 5.3 | 0 | 0 | 0 | 1 | 0 | 0 |
| 627 | 0 |  | 1 | 51 | 25.0 | 81.0 | 91 | 151 | 53 | 83 | 66 | 7.7 | 33.5 | 153 | 97 | 5.4 | 0 | 0 | 0 | 1 | 0 | 0 |
| 628 | 1 | 1 | 1 | 62 | 30.2 | 96.0 | 84 | 209 | 33 | 149 | 77 | 7.7 | 29.7 | 131 | 86 | 5.5 | 1 | 0 | 1 | 1 | 0 | 1 |
| 629 | 0 |  | 0 | 58 | 21.8 | 70.0 | 86 | 236 | 82 | 135 | 42 | 5.6 | 31.0 | 130 | 83 | 5.6 | 0 | 0 | 0 | 1 | 0 | 0 |
| 630 | 0 |  | 0 | 51 | 26.5 | 89.0 | 97 | 171 | 36 | 113 | 94 | 6.2 | 28.8 | 134 | 91 | 5.5 | 1 | 0 | 1 | 1 | 0 | 1 |
| 631 | 1 | 1 | 1 | 37 | 32.5 | 102.0 | 90 | 164 | 40 | 94 | 223 | 6.6 | 20.5 | 129 | 88 | 5.3 | 1 | 1 | 0 | 1 | 0 | 1 |
| 632 | 0 |  | 0 | 32 | 18.4 | 64.0 | 98 | 209 | 73 | 105 | 57 | 4.0 | 36.1 | 105 | 73 | 4.9 | 0 | 0 | 0 | 0 | 0 | 0 |
| 633 | 1 | 1 | 1 | 53 | 25.0 | 80.0 | 93 | 206 | 50 | 135 | 103 | 5.6 | 28.9 | 120 | 91 | 5.2 | 0 | 0 | 0 | 1 | 0 | 0 |
| 634 | 0 |  | 1 | 61 | 25.3 | 88.0 | 158 | 128 | 43 | 69 | 56 | 6.2 | 35.9 | 129 | 85 | 6.2 | 0 | 0 | 0 | 1 | 1 | 0 |
| 635 | 1 | 0 | 0 | 58 | 21.6 | 73.0 | 93 | 228 | 59 | 144 | 115 | 5.2 | 29.0 | 136 | 86 | 5.4 | 0 | 0 | 0 | 1 | 0 | 0 |
| 636 | 0 |  | 1 | 58 | 25.1 | 86.0 | 104 | 144 | 55 | 58 | 150 | 7.0 | 27.8 | 133 | 78 | 5.8 | 0 | 1 | 0 | 1 | 1 | 1 |
| 637 | 0 |  | 0 | 40 | 18.5 | 70.0 | 89 | 169 | 60 | 88 | 65 | 4.2 | 20.5 | 93 | 66 | 4.9 | 0 | 0 | 0 | 1 | 0 | 0 |
| 638 | 0 |  | 0 | 30 | 21.7 | 75.0 | 91 | 153 | 52 | 82 | 118 | 4.2 | 48.3 | 138 | 90 | 4.7 | 0 | 0 | 0 | 1 | 0 | 0 |
| 639 | 1 | 1 | 1 | 45 | 25.0 | 83.0 | 78 | 246 | 38 | 181 | 120 | 9.2 | 25.3 | 128 | 80 | 5.5 | 0 | 0 | 1 | 0 | 0 | 0 |
| 640 | 1 | 1 | 1 | 48 | 24.9 | 82.0 | 98 | 218 | 33 | 136 | 216 | 4.3 | 37.2 | 150 | 99 | 5.5 | 0 | 1 | 1 | 1 | 0 | 1 |
| 641 | 0 |  | 0 | 32 | 21.9 | 73.5 | 76 | 159 | 46 | 102 | 60 | 4.9 | 25.5 | 101 | 64 | 4.8 | 0 | 0 | 1 | 0 | 0 | 0 |
| 642 | 1 | 0 | 1 | 55 | 24.0 | 76.0 | 230 | 124 | 44 | 65 | 61 | 5.7 | 19.0 | 153 | 88 | 14.8 | 0 | 0 | 0 | 1 | 1 | 0 |
| 643 | 0 |  | 0 | 55 | 24.8 | 85.0 | 95 | 165 | 63 | 90 | 48 | 4.8 | 17.7 | 128 | 60 | 5.8 | 1 | 0 | 0 | 0 | 1 | 0 |
| 644 | 0 |  | 1 | 34 | 22.4 | 74.0 | 89 | 148 | 52 | 82 | 57 | 5.9 | 17.7 | 126 | 85 | 5.0 | 0 | 0 | 0 | 1 | 0 | 0 |
| 645 | 0 |  | 1 | 54 | 28.0 | 91.0 | 113 | 228 | 41 | 171 | 136 | 7.7 | 42.5 | 127 | 78 | 6.3 | 1 | 0 | 0 | 0 | 1 | 0 |
| 646 | 0 |  | 0 | 39 | 24.3 | 70.0 | 92 | 180 | 62 | 99 | 55 | 5.1 | 29.8 | 97 | 65 | 5.2 | 0 | 0 | 0 | 1 | 0 | 0 |
| 647 | 0 |  | 1 | 42 | 28.3 | 95.5 | 93 | 264 | 46 | 197 | 170 | 10.9 | 28.5 | 108 | 72 | 5.3 | 1 | 1 | 0 | 0 | 0 | 0 |
| 648 | 1 | 1 | 1 | 61 | 23.3 | 87.0 | 102 | 176 | 59 | 93 | 98 | 7.3 | 32.4 | 109 | 69 | 5.6 | 0 | 0 | 0 | 1 | 1 | 0 |
| 649 | 0 |  | 0 | 53 | 24.2 | 76.0 | 96 | 203 | 30 | 120 | 161 | 6.0 | 33.1 | 109 | 68 | 5.5 | 0 | 1 | 1 | 0 | 0 | 0 |
| 650 | 0 |  | 0 | 49 | 26.0 | 82.0 | 97 | 157 | 53 | 97 | 48 | 4.7 | 27.1 | 131 | 72 | 5.2 | 1 | 0 | 0 | 1 | 0 | 0 |
| 651 | 0 |  | 1 | 39 | 28.0 | 78.0 | 121 | 249 | 41 | 92 | 694 | 9.9 | 32.0 | 137 | 91 | 5.6 | 0 | 1 | 0 | 1 | 1 | 1 |
| 652 | 1 | 0 | 0 | 46 | 19.3 | 69.0 | 81 | 212 | 79 | 124 | 31 | 5.9 | 34.5 | 117 | 55 | 5.0 | 0 | 0 | 0 | 0 | 0 | 0 |
| 653 | 0 |  | 1 | 34 | 25.9 | 89.0 | 102 | 276 | 49 | 203 | 169 | 6.8 | 14.6 | 127 | 78 | 5.7 | 0 | 1 | 0 | 0 | 1 | 0 |
| 654 | 0 |  | 0 | 49 | 18.0 | 64.0 | 88 | 251 | 72 | 157 | 48 | 4.3 | 27.9 | 102 | 70 | 5.3 | 0 | 0 | 0 | 0 | 0 | 0 |
| 655 | 1 | 1 | 1 | 57 | 22.0 | 80.0 | 191 | 187 | 48 | 127 | 84 | 6.8 | 53.6 | 126 | 75 | 7.7 | 0 | 0 | 0 | 0 | 1 | 0 |
| 656 | 0 |  | 1 | 33 | 35.9 | 110.0 | 94 | 174 | 28 | 136 | 84 | 8.6 | 23.9 | 125 | 87 | 5.4 | 1 | 0 | 1 | 1 | 0 | 1 |
| 657 | 1 | 0 | 1 | 40 | 24.5 | 85.0 | 159 | 232 | 38 | 176 | 158 | 4.7 | 17.8 | 124 | 72 | 8.9 | 0 | 1 | 1 | 0 | 1 | 1 |
| 658 | 0 |  | 0 | 36 | 20.8 | 68.0 | 180 | 112 | 39 | 52 | 85 | 4.7 | 18.8 | 125 | 78 | 8.1 | 0 | 0 | 1 | 0 | 1 | 0 |
| 659 | 0 |  | 1 | 36 | 38.3 | 122.0 | 93 | 219 | 31 | 79 | 590 | 9.7 | 26.9 | 123 | 73 | 5.8 | 1 | 1 | 1 | 0 | 1 | 1 |
| 660 | 1 | 0 | 0 | 47 | 19.7 | 66.0 | 82 | 168 | 42 | 113 | 39 | 4.8 | 11.6 | 116 | 67 | 5.2 | 0 | 0 | 1 | 0 | 0 | 0 |
| 661 | 0 |  | 1 | 44 | 22.9 | 79.0 | 95 | 198 | 55 | 119 | 83 | 5.4 | 31.6 | 107 | 70 | 5.3 | 0 | 0 | 0 | 0 | 0 | 0 |
| 662 | 1 | 0 | 1 | 44 | 26.8 | 94.0 | 85 | 186 | 34 | 134 | 81 | 6.4 | 33.8 | 128 | 80 | 5.4 | 1 | 0 | 1 | 0 | 0 | 0 |
| 663 | 0 |  | 0 | 44 | 20.1 | 70.0 | 96 | 132 | 55 | 60 | 85 | 3.5 | 14.3 | 105 | 68 | 5.6 | 0 | 0 | 0 | 0 | 0 | 0 |
| 664 | 0 |  | 0 | 49 | 23.9 | 80.5 | 89 | 172 | 55 | 97 | 69 | 4.3 | 16.7 | 154 | 111 | 5.3 | 1 | 0 | 0 | 1 | 0 | 0 |
| 665 | 0 |  | 1 | 49 | 28.9 | 93.5 | 137 | 141 | 33 | 71 | 250 | 6.4 | 36.7 | 130 | 79 | 6.2 | 1 | 1 | 1 | 1 | 1 | 1 |
| 666 | 1 | 0 | 0 | 57 | 19.3 | 64.5 | 93 | 292 | 114 | 144 | 47 | 4.7 | 19.8 | 126 | 64 | 6.1 | 0 | 0 | 0 | 0 | 1 | 0 |
| 667 | 0 |  | 0 | 42 | 20.7 | 68.0 | 97 | 209 | 63 | 133 | 57 | 4.8 | 22.0 | 122 | 79 | 4.9 | 0 | 0 | 0 | 0 | 0 | 0 |
| 668 | 1 | 1 | 0 | 61 | 27.1 | 84.5 | 108 | 185 | 48 | 118 | 95 | 4.0 | 24.8 | 137 | 68 | 7.0 | 1 | 0 | 1 | 1 | 1 | 1 |
| 669 | 1 | 1 | 1 | 55 | 25.1 | 91.0 | 89 | 238 | 53 | 164 | 180 | 6.0 | 35.0 | 126 | 91 | 5.3 | 1 | 1 | 0 | 1 | 0 | 1 |
| 670 | 1 | 1 | 1 | 53 | 29.4 | 100.0 | 161 | 171 | 40 | 88 | 226 | 8.2 | 23.8 | 110 | 75 | 7.1 | 1 | 1 | 0 | 0 | 1 | 1 |
| 671 | 0 |  | 1 | 49 | 25.6 | 79.0 | 100 | 271 | 51 | 210 | 99 | 9.9 | 24.5 | 116 | 74 | 5.5 | 0 | 0 | 0 | 0 | 1 | 0 |
| 672 | 1 | 0 | 1 | 39 | 29.7 | 98.0 | 100 | 277 | 42 | 207 | 155 | 7.8 | 15.8 | 131 | 87 | 6.3 | 1 | 1 | 0 | 1 | 1 | 1 |
| 673 | 0 |  | 0 | 41 | 21.1 | 76.0 | 83 | 170 | 71 | 81 | 60 | 4.8 | 21.7 | 104 | 61 | 5.5 | 0 | 0 | 0 | 0 | 0 | 0 |
| 674 | 0 |  | 0 | 54 | 20.7 | 70.0 | 88 | 242 | 68 | 163 | 66 | 4.5 | 14.5 | 137 | 81 | 5.9 | 0 | 0 | 0 | 1 | 1 | 0 |
| 675 | 0 |  | 1 | 59 | 27.0 | 95.5 | 87 | 194 | 40 | 128 | 188 | 6.2 | 38.4 | 130 | 84 | 5.2 | 1 | 1 | 0 | 1 | 0 | 1 |
| 676 | 1 | 1 | 1 | 42 | 30.1 | 94.0 | 96 | 264 | 34 | 188 | 117 | 8.7 | 30.4 | 135 | 88 | 5.2 | 1 | 0 | 1 | 1 | 0 | 1 |
| 677 | 0 |  | 1 | 55 | 28.1 | 96.5 | 93 | 225 | 35 | 148 | 271 | 6.1 | 31.0 | 111 | 73 | 5.7 | 1 | 1 | 1 | 0 | 0 | 1 |
| 678 | 0 |  | 1 | 36 | 20.5 | 78.0 | 122 | 246 | 46 | 175 | 119 | 6.8 | 36.0 | 114 | 81 | 5.2 | 0 | 0 | 0 | 0 | 1 | 0 |
| 679 | 1 | 1 | 1 | 34 | 26.2 | 85.0 | 95 | 247 | 49 | 171 | 89 | 7.2 | 37.5 | 130 | 77 | 5.1 | 0 | 0 | 0 | 1 | 0 | 0 |
| 680 | 0 |  | 0 | 39 | 20.8 | 74.0 | 87 | 202 | 56 | 131 | 62 | 4.2 | 45.5 | 94 | 67 | 5.5 | 0 | 0 | 0 | 1 | 0 | 0 |
| 681 | 1 | 0 | 1 | 51 | 22.2 | 82.0 | 80 | 217 | 44 | 146 | 149 | 7.7 | 24.5 | 112 | 75 | 4.4 | 0 | 0 | 0 | 0 | 0 | 0 |
| 682 | 1 | 0 | 0 | 37 | 22.6 | 81.0 | 91 | 158 | 41 | 98 | 92 | 3.6 | 11.6 | 108 | 60 | 5.3 | 1 | 0 | 1 | 0 | 0 | 0 |
| 683 | 1 | 1 | 1 | 33 | 23.8 | 81.0 | 104 | 199 | 40 | 125 | 228 | 4.9 | 14.2 | 129 | 77 | 4.8 | 0 | 1 | 0 | 0 | 1 | 0 |
| 684 | 0 |  | 1 | 38 | 29.1 | 99.0 | 84 | 206 | 44 | 153 | 62 | 9.6 | 21.4 | 135 | 83 | 5.1 | 1 | 0 | 0 | 1 | 0 | 0 |
| 685 | 0 |  | 1 | 35 | 26.9 | 90.5 | 88 | 176 | 41 | 111 | 106 | 7.7 | 15.7 | 118 | 70 | 5.4 | 1 | 0 | 0 | 0 | 0 | 0 |
| 686 | 1 | 1 | 1 | 61 | 25.2 | 90.0 | 90 | 228 | 45 | 148 | 198 | 7.5 | 17.6 | 136 | 84 | 5.3 | 1 | 1 | 0 | 1 | 0 | 1 |
| 687 | 0 |  | 1 | 56 | 27.0 | 86.0 | 93 | 196 | 42 | 132 | 100 | 6.0 | 38.0 | 133 | 82 | 5.5 | 0 | 0 | 0 | 1 | 0 | 0 |
| 688 | 1 | 0 | 0 | 42 | 20.9 | 75.0 | 90 | 243 | 79 | 143 | 49 | 5.0 | 10.8 | 99 | 64 | 5.0 | 0 | 0 | 0 | 1 | 0 | 0 |
| 689 | 0 |  | 0 | 31 | 20.0 | 62.0 | 82 | 198 | 59 | 120 | 86 | 4.9 | 29.0 | 103 | 69 | 4.9 | 0 | 0 | 0 | 0 | 0 | 0 |
| 690 | 0 |  | 0 | 49 | 22.3 | 67.0 | 86 | 227 | 47 | 132 | 295 | 4.2 | 10.9 | 123 | 88 | 5.1 | 0 | 1 | 1 | 1 | 0 | 1 |
| 691 | 1 | 0 | 0 | 34 | 25.2 | 74.0 | 94 | 211 | 40 | 150 | 117 | 5.8 | 14.3 | 119 | 82 | 6.0 | 0 | 0 | 1 | 0 | 1 | 0 |
| 692 | 1 | 0 | 1 | 53 | 33.4 | 102.0 | 101 | 240 | 47 | 158 | 244 | 5.8 | 35.5 | 136 | 84 | 6.1 | 1 | 1 | 0 | 1 | 1 | 1 |
| 693 | 0 |  | 0 | 54 | 21.6 | 70.0 | 93 | 130 | 50 | 70 | 46 | 4.3 | 35.6 | 124 | 73 | 6.6 | 0 | 0 | 0 | 0 | 1 | 0 |
| 694 | 1 | 1 | 1 | 40 | 25.2 | 80.0 | 106 | 167 | 29 | 110 | 112 | 3.7 | 30.5 | 128 | 77 | 5.5 | 0 | 0 | 1 | 0 | 1 | 0 |
| 695 | 0 |  | 0 | 53 | 25.1 | 80.5 | 87 | 173 | 61 | 99 | 53 | 6.2 | 19.4 | 123 | 76 | 4.6 | 1 | 0 | 0 | 0 | 0 | 0 |
| 696 | 0 |  | 0 | 32 | 19.2 | 66.0 | 68 | 219 | 75 | 130 | 94 | 3.1 | 28.9 | 97 | 52 | 5.1 | 0 | 0 | 0 | 1 | 0 | 0 |
| 697 | 1 | 1 | 1 | 43 | 29.6 | 89.0 | 97 | 174 | 33 | 117 | 143 | 8.2 | 29.5 | 133 | 71 | 5.9 | 0 | 0 | 1 | 1 | 1 | 1 |
| 698 | 0 |  | 0 | 48 | 27.5 | 82.0 | 96 | 145 | 28 | 94 | 74 | 5.6 | 33.6 | 119 | 78 | 5.5 | 1 | 0 | 1 | 0 | 0 | 0 |
| 699 | 1 | 0 | 1 | 64 | 28.3 | 91.0 | 133 | 175 | 50 | 105 | 130 | 7.9 | 48.7 | 133 | 55 | 7.1 | 1 | 0 | 0 | 1 | 1 | 1 |
| 700 | 0 |  | 0 | 61 | 28.8 | 93.5 | 94 | 199 | 48 | 95 | 154 | 8.4 | 32.3 | 129 | 76 | 5.2 | 1 | 1 | 1 | 1 | 0 | 1 |
| 701 | 0 |  | 1 | 63 | 30.2 | 100.0 | 102 | 173 | 40 | 115 | 93 | 7.9 | 58.9 | 146 | 99 | 5.5 | 1 | 0 | 0 | 1 | 1 | 1 |
| 702 | 0 |  | 0 | 50 | 22.9 | 78.0 | 87 | 162 | 35 | 68 | 252 | 5.6 | 24.9 | 124 | 85 | 5.6 | 0 | 1 | 1 | 1 | 0 | 1 |
| 703 | 0 |  | 1 | 63 | 28.9 | 97.0 | 88 | 172 | 59 | 99 | 71 | 5.9 | 20.1 | 146 | 98 | 4.9 | 1 | 0 | 0 | 1 | 0 | 0 |
| 704 | 1 | 1 | 0 | 63 | 23.0 | 80.0 | 97 | 196 | 42 | 130 | 174 | 4.0 | 22.7 | 119 | 68 | 5.9 | 1 | 1 | 1 | 0 | 1 | 1 |
| 705 | 1 | 1 | 1 | 49 | 27.6 | 93.0 | 92 | 231 | 42 | 171 | 93 | 6.4 | 29.2 | 166 | 113 | 5.5 | 1 | 0 | 0 | 1 | 0 | 0 |
| 706 | 0 |  | 1 | 35 | 19.6 | 72.0 | 95 | 233 | 59 | 155 | 72 | 5.6 | 25.8 | 112 | 70 | 4.9 | 0 | 0 | 0 | 0 | 0 | 0 |
| 707 | 0 |  | 0 | 59 | 28.0 | 96.5 | 93 | 157 | 64 | 83 | 97 | 4.9 | 21.4 | 111 | 72 | 6.1 | 1 | 0 | 0 | 0 | 1 | 0 |
| 708 | 0 |  | 1 | 53 | 21.8 | 74.5 | 96 | 192 | 46 | 130 | 46 | 6.1 | 19.7 | 107 | 71 | 5.3 | 0 | 0 | 0 | 0 | 0 | 0 |
| 709 | 0 |  | 1 | 43 | 21.7 | 78.0 | 94 | 195 | 26 | 104 | 357 | 8.5 | 24.3 | 107 | 75 | 5.6 | 0 | 1 | 1 | 0 | 0 | 0 |
| 710 | 1 | 1 | 1 | 35 | 31.6 | 104.5 | 94 | 172 | 37 | 110 | 220 | 6.6 | 18.9 | 114 | 69 | 5.2 | 1 | 1 | 1 | 0 | 0 | 1 |
| 711 | 1 | 1 | 1 | 62 | 26.0 | 90.0 | 85 | 172 | 38 | 123 | 94 | 7.6 | 36.1 | 146 | 83 | 5.3 | 1 | 0 | 1 | 1 | 0 | 1 |
| 712 | 0 |  | 0 | 46 | 21.5 | 65.0 | 82 | 193 | 65 | 114 | 50 | 5.6 | 21.0 | 110 | 71 | 4.8 | 0 | 0 | 0 | 0 | 0 | 0 |
| 713 | 0 |  | 0 | 47 | 21.2 | 59.0 | 79 | 204 | 73 | 122 | 36 | 4.3 | 31.8 | 111 | 77 | 5.2 | 0 | 0 | 0 | 0 | 0 | 0 |
| 714 | 1 | 1 | 0 | 60 | 25.5 | 82.0 | 101 | 215 | 46 | 132 | 187 | 5.3 | 25.3 | 132 | 63 | 5.7 | 1 | 1 | 1 | 1 | 1 | 1 |
| 715 | 1 | 0 | 1 | 35 | 23.2 | 75.0 | 98 | 258 | 48 | 172 | 208 | 7.2 | 24.1 | 120 | 78 | 5.6 | 0 | 1 | 0 | 0 | 0 | 0 |
| 716 | 0 |  | 1 | 47 | 28.7 | 90.0 | 116 | 197 | 50 | 134 | 90 | 6.6 | 22.2 | 140 | 91 | 5.6 | 1 | 0 | 0 | 1 | 1 | 1 |
| 717 | 1 | 0 | 0 | 50 | 23.4 | 81.0 | 87 | 169 | 60 | 81 | 89 | 3.6 | 13.8 | 127 | 87 | 5.5 | 1 | 0 | 0 | 1 | 0 | 0 |
| 718 | 0 |  | 0 | 59 | 25.9 | 83.0 | 95 | 137 | 59 | 62 | 56 | 4.2 | 41.0 | 123 | 68 | 5.1 | 1 | 0 | 0 | 1 | 0 | 0 |
| 719 | 0 |  | 1 | 51 | 27.7 | 94.0 | 102 | 179 | 33 | 120 | 109 | 9.5 | 38.9 | 148 | 100 | 5.8 | 1 | 0 | 1 | 1 | 1 | 1 |
| 720 | 1 | 1 | 1 | 45 | 30.8 | 91.0 | 90 | 222 | 33 | 139 | 244 | 8.7 | 17.9 | 121 | 73 | 5.2 | 1 | 1 | 1 | 1 | 0 | 1 |
| 721 | 0 |  | 0 | 31 | 32.9 | 89.0 | 87 | 228 | 37 | 159 | 164 | 5.3 | 21.3 | 124 | 73 | 5.1 | 1 | 1 | 1 | 0 | 0 | 1 |
| 722 | 0 |  | 1 | 43 | 24.0 | 85.0 | 90 | 210 | 45 | 152 | 68 | 9.1 | 30.2 | 127 | 77 | 5.2 | 0 | 0 | 0 | 0 | 0 | 0 |
| 723 | 0 |  | 0 | 46 | 21.4 | 75.0 | 80 | 185 | 48 | 120 | 55 | 4.9 | 64.8 | 126 | 82 | 4.6 | 0 | 0 | 1 | 0 | 0 | 0 |
| 724 | 1 | 1 | 0 | 60 | 20.7 | 69.0 | 95 | 197 | 61 | 113 | 74 | 4.8 | 26.0 | 101 | 61 | 5.3 | 0 | 0 | 0 | 0 | 0 | 0 |
| 725 | 0 |  | 1 | 45 | 29.3 | 91.5 | 85 | 246 | 39 | 188 | 169 | 7.9 | 31.4 | 115 | 78 | 5.4 | 1 | 1 | 1 | 0 | 0 | 1 |
| 726 | 0 |  | 1 | 35 | 23.8 | 75.0 | 85 | 190 | 34 | 137 | 91 | 7.9 | 23.9 | 108 | 65 | 5.1 | 0 | 0 | 1 | 0 | 0 | 0 |
| 727 | 1 | 1 | 0 | 60 | 22.2 | 76.0 | 102 | 255 | 39 | 161 | 211 | 6.1 | 29.4 | 140 | 83 | 5.6 | 0 | 1 | 1 | 1 | 1 | 1 |
| 728 | 0 |  | 0 | 38 | 17.6 | 64.0 | 81 | 114 | 49 | 46 | 75 | 5.2 | 25.6 | 120 | 78 | 5.3 | 0 | 0 | 1 | 0 | 0 | 0 |
| 729 | 0 |  | 0 | 39 | 22.8 | 70.0 | 92 | 201 | 52 | 137 | 74 | 6.6 | 19.5 | 124 | 74 | 5.1 | 0 | 0 | 0 | 0 | 0 | 0 |
| 730 | 0 |  | 0 | 53 | 20.1 | 72.0 | 104 | 224 | 103 | 119 | 39 | 5.4 | 35.6 | 154 | 96 | 5.5 | 0 | 0 | 0 | 1 | 1 | 0 |
| 731 | 0 |  | 0 | 33 | 22.8 | 76.5 | 90 | 248 | 72 | 159 | 63 | 5.0 | 32.5 | 98 | 54 | 5.3 | 0 | 0 | 0 | 1 | 0 | 0 |
| 732 | 0 |  | 0 | 46 | 21.4 | 84.0 | 94 | 239 | 59 | 161 | 89 | 4.9 | 24.5 | 146 | 78 | 5.2 | 1 | 0 | 0 | 1 | 0 | 0 |
| 733 | 0 |  | 1 | 47 | 23.4 | 76.0 | 80 | 197 | 77 | 98 | 60 | 6.9 | 19.7 | 125 | 73 | 5.3 | 0 | 0 | 0 | 0 | 0 | 0 |
| 734 | 1 | 1 | 1 | 44 | 25.3 | 88.0 | 101 | 178 | 29 | 114 | 195 | 5.6 | 27.2 | 102 | 73 | 5.4 | 0 | 1 | 1 | 0 | 1 | 1 |
| 735 | 0 |  | 0 | 63 | 28.0 | 84.0 | 132 | 205 | 41 | 143 | 149 | 4.6 | 27.5 | 174 | 91 | 6.2 | 1 | 0 | 1 | 1 | 1 | 1 |
| 736 | 0 |  | 1 | 40 | 21.8 | 75.0 | 86 | 248 | 54 | 176 | 86 | 7.5 | 25.1 | 125 | 83 | 5.3 | 0 | 0 | 0 | 0 | 0 | 0 |
| 737 | 0 |  | 0 | 39 | 28.2 | 85.0 | 94 | 153 | 44 | 100 | 77 | 4.4 | 6.5 | 118 | 77 | 5.2 | 1 | 0 | 1 | 0 | 0 | 0 |
| 738 | 0 |  | 0 | 60 | 22.5 | 74.0 | 62 | 159 | 69 | 69 | 68 | 5.4 | 15.4 | 103 | 57 | 5.5 | 0 | 0 | 0 | 0 | 0 | 0 |
| 739 | 0 |  | 1 | 46 | 20.9 | 74.0 | 77 | 177 | 47 | 106 | 58 | 8.6 | 36.3 | 103 | 70 | 5.3 | 0 | 0 | 0 | 0 | 0 | 0 |
| 740 | 0 |  | 1 | 46 | 23.1 | 83.0 | 78 | 189 | 54 | 117 | 44 | 9.3 | 24.0 | 125 | 76 | 5.4 | 0 | 0 | 0 | 0 | 0 | 0 |
| 741 | 0 |  | 1 | 46 | 21.8 | 76.0 | 86 | 165 | 43 | 94 | 120 | 5.2 | 20.1 | 118 | 76 | 5.1 | 0 | 0 | 0 | 0 | 0 | 0 |
| 742 | 1 | 1 | 1 | 62 | 24.5 | 81.0 | 125 | 200 | 46 | 116 | 221 | 5.9 | 26.9 | 139 | 95 | 5.6 | 0 | 1 | 0 | 1 | 1 | 1 |
| 743 | 0 |  | 0 | 40 | 18.1 | 64.0 | 87 | 167 | 62 | 86 | 52 | 3.5 | 23.1 | 107 | 59 | 5.1 | 0 | 0 | 0 | 0 | 0 | 0 |
| 744 | 1 | 1 | 1 | 63 | 23.2 | 81.0 | 86 | 199 | 61 | 117 | 38 | 6.3 | 38.1 | 118 | 58 | 5.5 | 0 | 0 | 0 | 0 | 0 | 0 |
| 745 | 1 | 1 | 0 | 62 | 28.0 | 89.0 | 92 | 169 | 25 | 76 | 306 | 5.4 | 27.3 | 114 | 78 | 6.3 | 1 | 1 | 1 | 1 | 1 | 1 |
| 746 | 1 | 0 | 1 | 61 | 24.9 | 82.0 | 90 | 165 | 33 | 88 | 269 | 6.0 | 33.4 | 112 | 70 | 5.6 | 0 | 1 | 1 | 1 | 0 | 1 |
| 747 | 0 |  | 0 | 36 | 23.2 | 78.0 | 101 | 167 | 53 | 91 | 104 | 3.1 | 23.1 | 113 | 71 | 5.3 | 0 | 0 | 0 | 0 | 1 | 0 |
| 748 | 0 |  | 0 | 51 | 29.8 | 90.0 | 108 | 180 | 43 | 112 | 133 | 5.4 | 17.0 | 110 | 78 | 6.8 | 1 | 0 | 1 | 0 | 1 | 1 |
| 749 | 1 | 0 | 0 | 53 | 26.3 | 75.0 | 112 | 187 | 45 | 113 | 162 | 5.5 | 19.5 | 126 | 69 | 5.8 | 0 | 1 | 1 | 0 | 1 | 1 |
| 750 | 1 | 1 | 1 | 62 | 24.5 | 81.0 | 78 | 235 | 34 | 159 | 154 | 9.2 | 27.9 | 135 | 92 | 5.2 | 0 | 1 | 1 | 1 | 0 | 1 |
| 751 | 0 |  | 0 | 45 | 22.6 | 80.0 | 74 | 188 | 80 | 84 | 40 | 7.5 | 24.6 | 118 | 65 | 5.3 | 1 | 0 | 0 | 0 | 0 | 0 |
| 752 | 0 |  | 0 | 52 | 20.2 | 67.0 | 95 | 194 | 42 | 130 | 106 | 4.3 | 21.0 | 101 | 69 | 5.6 | 0 | 0 | 1 | 0 | 0 | 0 |
| 753 | 0 |  | 0 | 54 | 22.9 | 72.0 | 92 | 235 | 79 | 134 | 64 | 4.4 | 25.8 | 108 | 70 | 5.4 | 0 | 0 | 0 | 0 | 0 | 0 |
| 754 | 1 | 0 | 1 | 45 | 25.2 | 78.0 | 88 | 214 | 45 | 168 | 67 | 6.5 | 17.7 | 123 | 88 | 5.4 | 0 | 0 | 0 | 1 | 0 | 0 |
| 755 | 1 | 0 | 1 | 45 | 23.7 | 81.0 | 94 | 246 | 43 | 189 | 118 | 6.7 | 17.4 | 124 | 69 | 5.6 | 0 | 0 | 0 | 0 | 0 | 0 |
| 756 | 1 | 1 | 0 | 42 | 26.0 | 78.0 | 87 | 178 | 42 | 118 | 86 | 5.0 | 31.6 | 127 | 79 | 4.7 | 0 | 0 | 1 | 0 | 0 | 0 |
| 757 | 0 |  | 1 | 33 | 29.2 | 89.0 | 86 | 197 | 41 | 146 | 126 | 8.3 | 27.4 | 132 | 82 | 5.2 | 0 | 0 | 0 | 1 | 0 | 0 |
| 758 | 0 |  | 0 | 50 | 26.7 | 81.0 | 78 | 219 | 60 | 149 | 106 | 4.7 | 21.3 | 174 | 86 | 5.2 | 1 | 0 | 0 | 1 | 0 | 0 |
| 759 | 0 |  | 0 | 37 | 18.9 | 65.0 | 84 | 212 | 68 | 125 | 53 | 4.4 | 27.8 | 86 | 54 | 5.4 | 0 | 0 | 0 | 1 | 0 | 0 |
| 760 | 0 |  | 0 | 37 | 22.3 | 71.0 | 67 | 240 | 59 | 152 | 116 | 5.7 | 13.9 | 103 | 57 | 5.0 | 0 | 0 | 0 | 0 | 0 | 0 |
| 761 | 0 |  | 0 | 34 | 16.9 | 60.0 | 85 | 194 | 59 | 113 | 53 | 3.6 | 23.8 | 112 | 82 | 5.4 | 0 | 0 | 0 | 0 | 0 | 0 |
| 762 | 0 |  | 0 | 51 | 22.6 | 74.0 | 96 | 186 | 51 | 119 | 92 | 5.2 | 18.1 | 121 | 74 | 5.4 | 0 | 0 | 0 | 0 | 0 | 0 |
| 763 | 0 |  | 0 | 34 | 22.7 | 74.0 | 88 | 152 | 41 | 98 | 36 | 6.3 | 20.2 | 114 | 74 | 5.4 | 0 | 0 | 1 | 0 | 0 | 0 |
| 764 | 0 |  | 1 | 47 | 31.1 | 103.0 | 102 | 242 | 33 | 161 | 290 | 7.2 | 20.4 | 145 | 105 | 5.7 | 1 | 1 | 1 | 1 | 1 | 1 |
| 765 | 0 |  | 0 | 33 | 18.8 | 64.5 | 62 | 221 | 68 | 139 | 40 | 4.8 | 33.7 | 106 | 70 | 5.2 | 0 | 0 | 0 | 0 | 0 | 0 |
| 766 | 0 |  | 0 | 39 | 20.8 | 66.0 | 93 | 203 | 48 | 122 | 111 | 4.8 | 18.0 | 120 | 67 | 4.8 | 0 | 0 | 1 | 0 | 0 | 0 |
| 767 | 0 |  | 0 | 48 | 23.0 | 76.0 | 88 | 209 | 64 | 131 | 65 | 4.5 | 19.5 | 113 | 71 | 4.9 | 0 | 0 | 0 | 0 | 0 | 0 |
| 768 | 0 |  | 0 | 32 | 31.6 | 91.0 | 80 | 169 | 50 | 102 | 132 | 5.5 | 31.6 | 110 | 66 | 5.5 | 1 | 0 | 0 | 0 | 0 | 0 |
| 769 | 0 |  | 1 | 30 | 22.4 | 69.0 | 98 | 223 | 58 | 156 | 72 | 6.1 | 44.2 | 126 | 71 | 5.2 | 0 | 0 | 0 | 0 | 0 | 0 |
| 770 | 1 | 1 | 1 | 60 | 30.7 | 107.0 | 98 | 226 | 43 | 168 | 101 | 7.9 | 31.3 | 131 | 74 | 5.9 | 1 | 0 | 0 | 1 | 1 | 1 |
| 771 | 0 |  | 0 | 55 | 27.2 | 86.0 | 100 | 185 | 41 | 134 | 74 | 6.5 | 42.0 | 159 | 82 | 5.4 | 1 | 0 | 1 | 1 | 1 | 1 |
| 772 | 0 |  | 0 | 50 | 24.3 | 77.0 | 93 | 200 | 52 | 114 | 129 | 4.8 | 22.4 | 111 | 71 | 5.8 | 0 | 0 | 0 | 0 | 1 | 0 |
| 773 | 0 |  | 1 | 34 | 30.0 | 90.0 | 85 | 157 | 53 | 97 | 35 | 8.3 | 21.8 | 113 | 71 | 5.4 | 1 | 0 | 0 | 0 | 0 | 0 |
| 774 | 1 | 0 | 1 | 45 | 28.1 | 98.0 | 92 | 190 | 33 | 117 | 204 | 9.7 | 40.2 | 100 | 68 | 3.9 | 1 | 1 | 1 | 0 | 0 | 1 |
| 775 | 0 |  | 0 | 38 | 31.9 | 87.0 | 87 | 180 | 40 | 131 | 87 | 6.3 | 18.7 | 122 | 72 | 4.7 | 1 | 0 | 1 | 0 | 0 | 0 |
| 776 | 0 |  | 1 | 43 | 23.5 | 80.0 | 111 | 248 | 57 | 172 | 169 | 7.7 | 31.7 | 127 | 86 | 5.4 | 0 | 1 | 0 | 1 | 1 | 1 |
| 777 | 0 |  | 1 | 35 | 21.6 | 70.0 | 87 | 225 | 51 | 144 | 82 | 6.0 | 23.2 | 116 | 80 | 4.9 | 0 | 0 | 0 | 0 | 0 | 0 |
| 778 | 1 | 0 | 1 | 60 | 25.4 | 91.0 | 111 | 250 | 44 | 171 | 141 | 5.4 | 32.9 | 144 | 101 | 5.6 | 1 | 0 | 0 | 1 | 1 | 1 |
| 779 | 0 |  | 1 | 54 | 21.5 | 83.0 | 80 | 204 | 44 | 145 | 61 | 7.5 | 29.1 | 142 | 83 | 5.9 | 0 | 0 | 0 | 1 | 1 | 0 |
| 780 | 1 | 0 | 1 | 58 | 30.4 | 104.0 | 116 | 171 | 33 | 115 | 172 | 6.9 | 21.1 | 127 | 94 | 6.7 | 1 | 1 | 1 | 1 | 1 | 1 |
| 781 | 0 |  | 0 | 55 | 20.5 | 68.0 | 96 | 252 | 60 | 163 | 39 | 5.5 | 15.7 | 80 | 49 | 5.8 | 0 | 0 | 0 | 1 | 1 | 0 |
| 782 | 0 |  | 0 | 34 | 24.6 | 79.0 | 95 | 178 | 37 | 127 | 110 | 5.5 | 18.3 | 108 | 57 | 4.8 | 0 | 0 | 1 | 0 | 0 | 0 |
| 783 | 0 |  | 1 | 37 | 29.9 | 96.5 | 86 | 142 | 39 | 91 | 63 | 7.3 | 16.1 | 130 | 85 | 5.5 | 1 | 0 | 1 | 1 | 0 | 1 |
| 784 | 0 |  | 1 | 55 | 23.7 | 80.0 | 92 | 150 | 61 | 72 | 66 | 5.7 | 33.7 | 116 | 82 | 5.2 | 0 | 0 | 0 | 0 | 0 | 0 |
| 785 | 0 |  | 1 | 48 | 27.8 | 87.0 | 92 | 205 | 50 | 131 | 108 | 8.5 | 25.0 | 128 | 87 | 5.1 | 0 | 0 | 0 | 1 | 0 | 0 |
| 786 | 0 |  | 0 | 62 | 21.8 | 73.5 | 96 | 188 | 52 | 116 | 80 | 5.4 | 33.9 | 197 | 84 | 5.5 | 0 | 0 | 0 | 1 | 0 | 0 |
| 787 | 1 | 1 | 0 | 58 | 27.0 | 90.0 | 102 | 186 | 44 | 122 | 80 | 6.2 | 22.2 | 128 | 71 | 5.5 | 1 | 0 | 1 | 0 | 1 | 1 |
| 788 | 0 |  | 0 | 56 | 24.8 | 80.0 | 101 | 287 | 49 | 200 | 111 | 5.0 | 38.3 | 95 | 57 | 5.2 | 1 | 0 | 1 | 1 | 1 | 1 |
| 789 | 0 |  | 0 | 58 | 25.3 | 77.0 | 86 | 224 | 44 | 158 | 97 | 5.4 | 32.7 | 116 | 63 | 5.8 | 0 | 0 | 1 | 0 | 1 | 0 |
| 790 | 0 |  | 1 | 33 | 20.5 | 77.5 | 83 | 194 | 59 | 111 | 81 | 5.9 | 13.4 | 117 | 71 | 5.2 | 0 | 0 | 0 | 0 | 0 | 0 |
| 791 | 0 |  | 0 | 37 | 24.4 | 83.0 | 86 | 184 | 82 | 84 | 45 | 4.2 | 17.5 | 105 | 70 | 5.1 | 1 | 0 | 0 | 0 | 0 | 0 |
| 792 | 0 |  | 1 | 37 | 20.2 | 72.0 | 83 | 295 | 51 | 235 | 50 | 6.6 | 33.6 | 121 | 76 | 5.4 | 0 | 0 | 0 | 0 | 0 | 0 |
| 793 | 0 |  | 0 | 50 | 25.7 | 74.0 | 89 | 182 | 54 | 113 | 77 | 4.6 | 27.1 | 133 | 94 | 5.1 | 0 | 0 | 0 | 1 | 0 | 0 |
| 794 | 0 |  | 1 | 63 | 22.5 | 72.0 | 90 | 245 | 84 | 137 | 76 | 4.9 | 59.9 | 130 | 82 | 5.6 | 0 | 0 | 0 | 1 | 0 | 0 |
| 795 | 0 |  | 0 | 56 | 21.2 | 70.0 | 102 | 349 | 45 | 263 | 112 | 6.2 | 24.6 | 122 | 79 | 5.5 | 0 | 0 | 1 | 0 | 1 | 0 |
| 796 | 0 |  | 0 | 57 | 23.3 | 74.0 | 93 | 227 | 50 | 157 | 59 | 5.1 | 24.9 | 146 | 81 | 5.6 | 0 | 0 | 0 | 1 | 0 | 0 |
| 797 | 0 |  | 0 | 41 | 21.4 | 66.0 | 96 | 205 | 89 | 106 | 25 | 4.0 | 19.3 | 106 | 63 | 5.3 | 0 | 0 | 0 | 0 | 0 | 0 |
| 798 | 1 | 0 | 1 | 43 | 26.4 | 86.0 | 107 | 247 | 45 | 195 | 146 | 5.7 | 29.6 | 109 | 73 | 6.1 | 0 | 0 | 0 | 0 | 1 | 0 |
| 799 | 0 |  | 0 | 55 | 21.6 | 61.0 | 94 | 208 | 60 | 119 | 128 | 4.3 | 23.3 | 121 | 69 | 4.9 | 0 | 0 | 0 | 0 | 0 | 0 |
| 800 | 0 |  | 0 | 32 | 18.0 | 66.0 | 82 | 229 | 77 | 139 | 97 | 4.1 | 8.5 | 106 | 71 | 5.0 | 0 | 0 | 0 | 0 | 0 | 0 |
| 801 | 1 | 1 | 0 | 43 | 20.2 | 59.0 | 107 | 213 | 65 | 127 | 74 | 4.6 | 19.0 | 109 | 67 | 5.1 | 0 | 0 | 0 | 0 | 1 | 0 |
| 802 | 1 | 0 | 0 | 53 | 30.4 | 87.0 | 105 | 121 | 32 | 71 | 127 | 5.9 | 17.1 | 113 | 69 | 5.6 | 1 | 0 | 1 | 0 | 1 | 1 |
| 803 | 1 | 0 | 1 | 39 | 29.1 | 99.5 | 97 | 293 | 72 | 194 | 96 | 9.7 | 39.3 | 123 | 71 | 5.1 | 1 | 0 | 0 | 0 | 0 | 0 |
| 804 | 0 |  | 1 | 30 | 23.8 | 75.0 | 82 | 228 | 43 | 167 | 98 | 7.6 | 16.7 | 130 | 78 | 5.5 | 0 | 0 | 0 | 1 | 0 | 0 |
| 805 | 1 | 1 | 1 | 37 | 34.8 | 104.0 | 83 | 257 | 44 | 186 | 144 | 9.9 | 26.1 | 115 | 82 | 5.4 | 1 | 0 | 0 | 1 | 0 | 0 |
| 806 | 0 |  | 0 | 40 | 21.3 | 73.5 | 91 | 216 | 78 | 111 | 78 | 4.9 | 31.0 | 123 | 79 | 4.7 | 0 | 0 | 0 | 0 | 0 | 0 |
| 807 | 0 |  | 1 | 49 | 26.3 | 84.0 | 100 | 278 | 34 | 202 | 157 | 5.8 | 31.5 | 123 | 81 | 5.5 | 0 | 1 | 1 | 0 | 1 | 1 |
| 808 | 1 | 0 | 0 | 51 | 22.5 | 74.0 | 75 | 235 | 67 | 141 | 49 | 5.4 | 20.0 | 106 | 66 | 4.4 | 0 | 0 | 0 | 0 | 0 | 0 |
| 809 | 1 | 1 | 1 | 53 | 25.8 | 82.0 | 87 | 203 | 57 | 126 | 108 | 5.7 | 25.2 | 120 | 74 | 5.3 | 0 | 0 | 0 | 0 | 0 | 0 |
| 810 | 1 | 0 | 1 | 53 | 25.4 | 82.0 | 127 | 147 | 42 | 84 | 107 | 5.8 | 30.2 | 126 | 75 | 6.2 | 0 | 0 | 0 | 0 | 1 | 0 |
| 811 | 0 |  | 0 | 41 | 22.8 | 77.0 | 86 | 208 | 37 | 163 | 81 | 4.7 | 37.9 | 106 | 56 | 5.3 | 0 | 0 | 1 | 0 | 0 | 0 |
| 812 | 0 |  | 0 | 41 | 17.3 | 61.0 | 92 | 194 | 59 | 121 | 64 | 4.0 | 24.5 | 102 | 62 | 5.0 | 0 | 0 | 0 | 0 | 0 | 0 |
| 813 | 1 | 0 | 0 | 46 | 26.0 | 80.0 | 101 | 255 | 49 | 179 | 115 | 4.7 | 14.6 | 126 | 75 | 5.5 | 1 | 0 | 1 | 0 | 1 | 1 |
| 814 | 0 |  | 1 | 36 | 26.5 | 87.0 | 93 | 209 | 43 | 122 | 233 | 6.2 | 18.3 | 128 | 76 | 5.7 | 0 | 1 | 0 | 0 | 0 | 0 |
| 815 | 1 | 0 | 0 | 44 | 18.6 | 64.0 | 81 | 147 | 54 | 79 | 38 | 4.0 | 18.9 | 118 | 84 | 4.7 | 0 | 0 | 0 | 0 | 0 | 0 |
| 816 | 1 | 1 | 0 | 44 | 28.2 | 90.0 | 99 | 185 | 40 | 138 | 84 | 5.9 | 9.9 | 127 | 73 | 6.1 | 1 | 0 | 1 | 0 | 1 | 1 |
| 817 | 0 |  | 0 | 35 | 19.2 | 64.5 | 91 | 195 | 60 | 124 | 61 | 3.8 | 25.4 | 94 | 63 | 5.1 | 0 | 0 | 0 | 1 | 0 | 0 |
| 818 | 0 |  | 0 | 55 | 19.9 | 69.0 | 99 | 264 | 54 | 181 | 84 | 4.7 | 17.7 | 100 | 50 | 5.7 | 0 | 0 | 0 | 0 | 0 | 0 |
| 819 | 1 | 0 | 0 | 36 | 20.5 | 71.0 | 92 | 205 | 52 | 124 | 156 | 4.5 | 16.2 | 99 | 71 | 5.4 | 0 | 1 | 0 | 1 | 0 | 0 |
| 820 | 0 |  | 0 | 61 | 24.8 | 80.0 | 166 | 217 | 49 | 158 | 101 | 6.3 | 18.5 | 120 | 69 | 7.0 | 1 | 0 | 1 | 0 | 1 | 1 |
| 821 | 0 |  | 0 | 45 | 18.5 | 59.0 | 98 | 252 | 69 | 177 | 49 | 5.7 | 25.7 | 113 | 72 | 5.4 | 0 | 0 | 0 | 0 | 0 | 0 |
| 822 | 0 |  | 1 | 56 | 27.1 | 94.0 | 113 | 238 | 34 | 157 | 185 | 7.7 | 40.4 | 132 | 81 | 6.4 | 1 | 1 | 1 | 1 | 1 | 1 |
| 823 | 0 |  | 1 | 61 | 23.8 | 80.0 | 121 | 210 | 43 | 146 | 88 | 9.9 | 16.4 | 133 | 78 | 5.9 | 0 | 0 | 0 | 1 | 1 | 0 |
| 824 | 0 |  | 1 | 48 | 26.0 | 91.0 | 95 | 179 | 26 | 102 | 299 | 9.7 | 21.0 | 135 | 80 | 5.6 | 1 | 1 | 1 | 1 | 0 | 1 |
| 825 | 1 | 0 | 1 | 55 | 26.0 | 86.0 | 82 | 153 | 42 | 100 | 61 | 7.3 | 29.2 | 135 | 87 | 5.1 | 0 | 0 | 0 | 1 | 0 | 0 |
| 826 | 1 | 1 | 0 | 58 | 22.7 | 85.5 | 93 | 165 | 53 | 104 | 52 | 6.7 | 31.1 | 107 | 64 | 5.7 | 1 | 0 | 0 | 0 | 1 | 0 |
| 827 | 1 | 1 | 1 | 46 | 28.8 | 93.5 | 90 | 169 | 52 | 103 | 54 | 5.1 | 48.5 | 139 | 89 | 5.1 | 1 | 0 | 0 | 1 | 0 | 0 |
| 828 | 1 | 1 | 0 | 53 | 20.0 | 78.0 | 81 | 225 | 73 | 129 | 51 | 4.9 | 25.3 | 115 | 66 | 4.8 | 0 | 0 | 0 | 0 | 0 | 0 |
| 829 | 1 | 1 | 0 | 42 | 20.1 | 70.0 | 88 | 183 | 69 | 99 | 64 | 4.6 | 36.8 | 112 | 79 | 5.2 | 0 | 0 | 0 | 0 | 0 | 0 |
| 830 | 0 |  | 0 | 44 | 21.5 | 70.5 | 79 | 134 | 58 | 69 | 55 | 3.7 | 29.1 | 112 | 72 | 5.0 | 0 | 0 | 0 | 0 | 0 | 0 |
| 831 | 0 |  | 0 | 56 | 20.5 | 68.0 | 96 | 222 | 66 | 138 | 66 | 4.3 | 29.3 | 123 | 75 | 5.3 | 0 | 0 | 0 | 0 | 0 | 0 |
| 832 | 0 |  | 0 | 51 | 22.8 | 68.0 | 80 | 230 | 50 | 166 | 85 | 4.5 | 30.7 | 132 | 74 | 5.5 | 0 | 0 | 0 | 1 | 0 | 0 |
| 833 | 0 |  | 0 | 36 | 22.8 | 64.0 | 79 | 186 | 81 | 85 | 60 | 5.5 | 18.7 | 109 | 70 | 5.2 | 0 | 0 | 0 | 0 | 0 | 0 |
| 834 | 0 |  | 0 | 41 | 20.2 | 63.0 | 80 | 126 | 50 | 64 | 47 | 4.2 | 14.8 | 94 | 71 | 5.3 | 0 | 0 | 0 | 1 | 0 | 0 |
| 835 | 0 |  | 1 | 53 | 21.7 | 78.0 | 84 | 244 | 49 | 177 | 57 | 4.8 | 22.4 | 104 | 63 | 5.4 | 0 | 0 | 0 | 0 | 0 | 0 |
| 836 | 1 | 1 | 0 | 40 | 21.4 | 61.0 | 82 | 230 | 70 | 131 | 57 | 5.0 | 22.0 | 116 | 70 | 4.8 | 0 | 0 | 0 | 0 | 0 | 0 |
| 837 | 0 |  | 0 | 40 | 20.3 | 63.0 | 79 | 205 | 77 | 112 | 62 | 4.5 | 14.6 | 101 | 59 | 5.0 | 0 | 0 | 0 | 0 | 0 | 0 |
| 838 | 1 | 1 | 0 | 50 | 23.1 | 71.0 | 90 | 265 | 54 | 186 | 87 | 5.2 | 28.5 | 113 | 85 | 5.4 | 0 | 0 | 0 | 1 | 0 | 0 |
| 839 | 1 | 1 | 1 | 44 | 22.6 | 80.0 | 96 | 206 | 52 | 124 | 187 | 8.8 | 24.5 | 131 | 63 | 5.4 | 0 | 1 | 0 | 1 | 0 | 0 |
| 840 | 1 | 1 | 0 | 31 | 17.6 | 64.0 | 78 | 214 | 68 | 134 | 38 | 4.9 | 22.4 | 95 | 64 | 4.9 | 0 | 0 | 0 | 1 | 0 | 0 |
| 841 | 1 | 1 | 1 | 44 | 25.1 | 87.0 | 84 | 180 | 41 | 132 | 49 | 5.0 | 32.2 | 113 | 64 | 5.4 | 0 | 0 | 0 | 0 | 0 | 0 |
| 842 | 0 |  | 0 | 41 | 20.9 | 64.0 | 85 | 202 | 78 | 99 | 81 | 3.7 | 25.0 | 100 | 52 | 5.1 | 0 | 0 | 0 | 0 | 0 | 0 |
| 843 | 0 |  | 1 | 33 | 25.4 | 78.0 | 99 | 143 | 50 | 67 | 125 | 8.0 | 16.3 | 122 | 86 | 5.3 | 0 | 0 | 0 | 1 | 0 | 0 |
| 844 | 0 |  | 1 | 40 | 24.9 | 79.0 | 83 | 169 | 40 | 117 | 74 | 7.4 | 25.8 | 143 | 74 | 4.9 | 0 | 0 | 0 | 1 | 0 | 0 |
| 845 | 1 | 1 | 1 | 60 | 33.5 | 102.0 | 119 | 168 | 32 | 99 | 191 | 7.7 | 31.7 | 153 | 104 | 5.6 | 1 | 1 | 1 | 1 | 1 | 1 |
| 846 | 1 | 1 | 0 | 31 | 21.7 | 68.0 | 91 | 206 | 63 | 119 | 73 | 3.6 | 14.6 | 109 | 61 | 4.3 | 0 | 0 | 0 | 0 | 0 | 0 |
| 847 | 1 | 0 | 1 | 33 | 23.0 | 76.5 | 85 | 208 | 63 | 118 | 68 | 4.5 | 26.1 | 139 | 74 | 5.1 | 0 | 0 | 0 | 1 | 0 | 0 |
| 848 | 1 | 1 | 0 | 49 | 21.7 | 70.0 | 90 | 230 | 63 | 144 | 53 | 5.2 | 21.6 | 116 | 68 | 5.5 | 0 | 0 | 0 | 0 | 0 | 0 |
| 849 | 0 |  | 0 | 56 | 22.5 | 72.0 | 84 | 231 | 65 | 156 | 46 | 5.1 | 32.0 | 125 | 80 | 5.5 | 0 | 0 | 0 | 0 | 0 | 0 |
| 850 | 0 |  | 1 | 50 | 26.7 | 93.0 | 83 | 203 | 42 | 137 | 152 | 4.4 | 24.1 | 122 | 83 | 5.4 | 1 | 1 | 0 | 1 | 0 | 1 |
| 851 | 0 |  | 0 | 38 | 20.9 | 68.0 | 79 | 187 | 65 | 106 | 72 | 4.1 | 30.4 | 124 | 73 | 5.2 | 0 | 0 | 0 | 0 | 0 | 0 |
| 852 | 0 |  | 0 | 36 | 21.4 | 69.0 | 85 | 207 | 75 | 112 | 60 | 3.8 | 22.4 | 108 | 59 | 5.3 | 0 | 0 | 0 | 0 | 0 | 0 |
| 853 | 0 |  | 0 | 41 | 22.2 | 78.0 | 91 | 154 | 50 | 91 | 45 | 4.7 | 20.2 | 127 | 70 | 5.6 | 0 | 0 | 0 | 0 | 0 | 0 |
| 854 | 1 | 0 | 0 | 58 | 34.5 | 93.0 | 115 | 263 | 66 | 154 | 162 | 8.3 | 19.3 | 133 | 82 | 5.9 | 1 | 1 | 0 | 1 | 1 | 1 |
| 855 | 0 |  | 0 | 41 | 25.8 | 86.0 | 94 | 195 | 41 | 129 | 148 | 4.4 | 16.6 | 139 | 80 | 5.8 | 1 | 0 | 1 | 1 | 1 | 1 |
| 856 | 0 |  | 1 | 36 | 27.3 | 79.0 | 78 | 205 | 64 | 127 | 32 | 7.7 | 29.3 | 115 | 69 | 5.7 | 0 | 0 | 0 | 0 | 0 | 0 |
| 857 | 0 |  | 0 | 52 | 27.8 | 95.0 | 88 | 215 | 63 | 142 | 57 | 5.3 | 37.8 | 106 | 73 | 4.8 | 1 | 0 | 0 | 0 | 0 | 0 |
| 858 | 0 |  | 0 | 48 | 33.3 | 97.0 | 104 | 239 | 37 | 166 | 228 | 5.9 | 19.7 | 131 | 77 | 5.8 | 1 | 1 | 1 | 1 | 1 | 1 |
| 859 | 0 |  | 1 | 59 | 25.9 | 86.0 | 105 | 203 | 39 | 139 | 100 | 6.8 | 39.7 | 115 | 70 | 5.6 | 0 | 0 | 1 | 0 | 1 | 0 |
| 860 | 1 | 1 | 1 | 64 | 24.9 | 86.0 | 106 | 161 | 36 | 93 | 182 | 6.5 | 34.1 | 123 | 80 | 5.7 | 0 | 1 | 1 | 0 | 1 | 1 |
| 861 | 0 |  | 1 | 54 | 27.6 | 88.5 | 108 | 193 | 52 | 117 | 94 | 4.5 | 42.5 | 121 | 84 | 5.5 | 0 | 0 | 0 | 1 | 1 | 0 |
| 862 | 1 | 1 | 1 | 59 | 29.7 | 92.5 | 100 | 193 | 39 | 106 | 160 | 7.7 | 34.0 | 148 | 87 | 5.5 | 1 | 1 | 1 | 1 | 1 | 1 |
| 863 | 0 |  | 0 | 42 | 20.7 | 72.0 | 84 | 148 | 50 | 91 | 40 | 3.4 | 7.7 | 93 | 55 | 5.4 | 0 | 0 | 0 | 1 | 0 | 0 |
| 864 | 0 |  | 0 | 41 | 25.8 | 83.5 | 86 | 288 | 73 | 191 | 75 | 4.5 | 16.8 | 131 | 87 | 5.5 | 1 | 0 | 0 | 1 | 0 | 0 |
| 865 | 0 |  | 1 | 46 | 30.3 | 90.0 | 96 | 284 | 31 | 129 | 582 | 8.0 | 24.6 | 121 | 77 | 6.1 | 1 | 1 | 1 | 0 | 1 | 1 |
| 866 | 1 | 0 | 0 | 60 | 23.7 | 74.0 | 92 | 209 | 47 | 151 | 62 | 7.0 | 29.7 | 137 | 81 | 5.4 | 0 | 0 | 1 | 1 | 0 | 0 |
| 867 | 0 |  | 0 | 36 | 25.4 | 84.0 | 99 | 234 | 42 | 180 | 87 | 4.6 | 26.4 | 99 | 70 | 5.3 | 1 | 0 | 1 | 1 | 0 | 1 |
| 868 | 1 | 1 | 1 | 34 | 25.2 | 87.0 | 86 | 174 | 70 | 93 | 32 | 5.0 | 17.1 | 122 | 81 | 5.2 | 0 | 0 | 0 | 0 | 0 | 0 |
| 869 | 0 |  | 1 | 58 | 27.0 | 84.0 | 108 | 199 | 52 | 131 | 54 | 6.0 | 29.1 | 121 | 75 | 5.2 | 0 | 0 | 0 | 0 | 1 | 0 |
| 870 | 1 | 1 | 1 | 56 | 25.3 | 84.0 | 82 | 241 | 32 | 183 | 167 | 6.0 | 38.5 | 129 | 79 | 5.2 | 0 | 1 | 1 | 0 | 0 | 0 |
| 871 | 0 |  | 0 | 42 | 19.2 | 65.0 | 92 | 154 | 59 | 82 | 49 | 5.0 | 19.9 | 108 | 51 | 5.3 | 0 | 0 | 0 | 0 | 0 | 0 |
| 872 | 1 | 0 | 1 | 43 | 30.0 | 93.0 | 90 | 283 | 42 | 219 | 96 | 6.1 | 30.5 | 140 | 80 | 3.8 | 1 | 0 | 0 | 1 | 0 | 0 |
| 873 | 0 |  | 1 | 45 | 26.4 | 94.0 | 92 | 234 | 46 | 148 | 177 | 7.5 | 28.7 | 119 | 92 | 5.1 | 1 | 1 | 0 | 1 | 0 | 1 |
| 874 | 0 |  | 1 | 62 | 23.7 | 83.0 | 115 | 286 | 52 | 212 | 132 | 8.3 | 56.9 | 158 | 95 | 6.2 | 0 | 0 | 0 | 1 | 1 | 0 |
| 875 | 1 | 1 | 1 | 61 | 28.9 | 100.0 | 133 | 286 | 45 | 169 | 478 | 9.6 | 29.2 | 169 | 104 | 5.3 | 1 | 1 | 0 | 1 | 1 | 1 |
| 876 | 1 | 1 | 0 | 49 | 23.7 | 70.0 | 96 | 201 | 38 | 119 | 148 | 3.7 | 22.7 | 119 | 78 | 5.0 | 0 | 0 | 1 | 0 | 0 | 0 |
| 877 | 0 |  | 0 | 34 | 19.2 | 64.0 | 72 | 151 | 52 | 86 | 35 | 6.7 | 22.5 | 100 | 62 | 5.2 | 0 | 0 | 0 | 0 | 0 | 0 |
| 878 | 0 |  | 1 | 44 | 20.5 | 72.0 | 82 | 216 | 40 | 146 | 98 | 6.5 | 28.9 | 116 | 88 | 4.8 | 0 | 0 | 0 | 1 | 0 | 0 |
| 879 | 1 | 1 | 1 | 44 | 24.5 | 81.0 | 92 | 157 | 35 | 102 | 103 | 6.3 | 42.5 | 128 | 74 | 5.1 | 0 | 0 | 1 | 0 | 0 | 0 |
| 880 | 1 | 1 | 0 | 45 | 21.7 | 68.0 | 100 | 201 | 57 | 120 | 92 | 7.2 | 17.8 | 94 | 63 | 4.0 | 0 | 0 | 0 | 1 | 1 | 0 |
| 881 | 0 |  | 1 | 43 | 25.0 | 89.5 | 88 | 175 | 36 | 127 | 42 | 5.7 | 28.4 | 121 | 81 | 5.5 | 0 | 0 | 1 | 0 | 0 | 0 |
| 882 | 1 | 1 | 1 | 47 | 27.3 | 90.0 | 93 | 204 | 67 | 125 | 59 | 6.1 | 20.1 | 111 | 62 | 5.1 | 1 | 0 | 0 | 0 | 0 | 0 |
| 883 | 0 |  | 1 | 58 | 24.4 | 74.0 | 85 | 195 | 47 | 131 | 65 | 8.7 | 29.5 | 133 | 77 | 5.2 | 0 | 0 | 0 | 1 | 0 | 0 |
| 884 | 1 | 1 | 1 | 62 | 24.0 | 86.0 | 98 | 184 | 36 | 106 | 167 | 7.5 | 43.4 | 139 | 84 | 5.2 | 0 | 1 | 1 | 1 | 0 | 1 |
| 885 | 1 | 0 | 0 | 45 | 21.5 | 72.0 | 100 | 165 | 65 | 80 | 26 | 4.5 | 11.9 | 103 | 71 | 5.3 | 0 | 0 | 0 | 0 | 1 | 0 |
| 886 | 0 |  | 0 | 52 | 20.3 | 65.0 | 85 | 223 | 88 | 118 | 32 | 4.6 | 32.1 | 156 | 93 | 5.3 | 0 | 0 | 0 | 1 | 0 | 0 |
| 887 | 0 |  | 0 | 64 | 22.8 | 79.5 | 93 | 287 | 75 | 188 | 93 | 4.9 | 27.5 | 148 | 71 | 5.2 | 0 | 0 | 0 | 1 | 0 | 0 |
| 888 | 1 | 0 | 1 | 62 | 27.8 | 89.0 | 174 | 174 | 43 | 131 | 58 | 4.5 | 31.9 | 103 | 75 | 7.5 | 0 | 0 | 0 | 0 | 1 | 0 |
| 889 | 0 |  | 0 | 31 | 22.6 | 74.0 | 86 | 210 | 59 | 143 | 46 | 5.1 | 31.3 | 96 | 68 | 5.5 | 0 | 0 | 0 | 1 | 0 | 0 |
| 890 | 1 | 1 | 1 | 36 | 22.1 | 81.0 | 72 | 227 | 53 | 165 | 44 | 6.2 | 25.1 | 116 | 66 | 5.0 | 0 | 0 | 0 | 0 | 0 | 0 |
| 891 | 0 |  | 1 | 33 | 21.3 | 71.0 | 88 | 216 | 39 | 167 | 107 | 5.3 | 25.2 | 122 | 74 | 5.3 | 0 | 0 | 1 | 0 | 0 | 0 |
| 892 | 0 |  | 1 | 36 | 25.9 | 89.5 | 115 | 244 | 31 | 144 | 315 | 6.2 | 38.8 | 141 | 73 | 5.4 | 0 | 1 | 1 | 1 | 1 | 1 |
| 893 | 1 | 0 | 0 | 61 | 23.4 | 72.0 | 96 | 253 | 77 | 156 | 39 | 4.4 | 43.8 | 109 | 64 | 5.1 | 0 | 0 | 0 | 0 | 0 | 0 |
| 894 | 0 |  | 0 | 55 | 20.8 | 70.0 | 105 | 222 | 48 | 162 | 42 | 6.6 | 20.8 | 109 | 66 | 6.1 | 0 | 0 | 1 | 0 | 1 | 0 |
| 895 | 0 |  | 1 | 32 | 20.9 | 71.0 | 91 | 188 | 43 | 126 | 117 | 6.4 | 23.3 | 142 | 90 | 5.1 | 0 | 0 | 0 | 1 | 0 | 0 |
| 896 | 0 |  | 0 | 59 | 20.8 | 86.0 | 77 | 246 | 72 | 159 | 79 | 4.1 | 36.4 | 110 | 70 | 5.0 | 1 | 0 | 0 | 0 | 0 | 0 |
| 897 | 0 |  | 1 | 48 | 22.9 | 75.0 | 85 | 211 | 43 | 156 | 61 | 5.8 | 16.9 | 113 | 76 | 5.2 | 0 | 0 | 0 | 0 | 0 | 0 |
| 898 | 1 | 0 | 1 | 60 | 23.6 | 81.0 | 94 | 216 | 48 | 128 | 126 | 7.5 | 38.0 | 115 | 74 | 5.3 | 0 | 0 | 0 | 0 | 0 | 0 |
| 899 | 0 |  | 0 | 55 | 26.6 | 87.0 | 103 | 242 | 50 | 171 | 74 | 6.0 | 22.0 | 140 | 64 | 4.3 | 1 | 0 | 0 | 1 | 1 | 1 |
| 900 | 0 |  | 1 | 35 | 27.0 | 85.0 | 92 | 209 | 36 | 167 | 73 | 8.8 | 24.0 | 129 | 82 | 5.7 | 0 | 0 | 1 | 0 | 0 | 0 |
| 901 | 1 | 1 | 1 | 62 | 23.1 | 82.5 | 94 | 180 | 45 | 119 | 86 | 7.3 | 37.0 | 139 | 80 | 5.4 | 0 | 0 | 0 | 1 | 0 | 0 |
| 902 | 0 |  | 0 | 60 | 19.8 | 66.0 | 87 | 208 | 64 | 118 | 109 | 3.1 | 38.8 | 115 | 72 | 5.3 | 0 | 0 | 0 | 0 | 0 | 0 |
| 903 | 0 |  | 0 | 35 | 20.8 | 68.5 | 79 | 194 | 52 | 128 | 48 | 3.9 | 26.8 | 109 | 53 | 5.0 | 0 | 0 | 0 | 0 | 0 | 0 |
| 904 | 0 |  | 0 | 33 | 18.9 | 72.0 | 92 | 178 | 57 | 102 | 77 | 4.9 | 16.8 | 102 | 72 | 5.0 | 0 | 0 | 0 | 0 | 0 | 0 |
| 905 | 0 |  | 1 | 36 | 21.9 | 78.0 | 99 | 192 | 53 | 116 | 98 | 5.8 | 25.6 | 124 | 75 | 5.3 | 0 | 0 | 0 | 0 | 0 | 0 |
| 906 | 0 |  | 0 | 32 | 19.9 | 64.0 | 71 | 185 | 61 | 107 | 35 | 4.8 | 24.3 | 104 | 59 | 5.1 | 0 | 0 | 0 | 0 | 0 | 0 |
| 907 | 0 |  | 0 | 50 | 23.0 | 71.5 | 82 | 164 | 28 | 117 | 77 | 5.6 | 19.4 | 126 | 63 | 5.4 | 0 | 0 | 1 | 0 | 0 | 0 |
| 908 | 1 | 1 | 1 | 60 | 29.5 | 99.0 | 89 | 172 | 30 | 100 | 257 | 9.1 | 14.3 | 132 | 71 | 5.1 | 1 | 1 | 1 | 1 | 0 | 1 |
| 909 | 1 | 1 | 1 | 54 | 29.9 | 102.0 | 105 | 169 | 42 | 95 | 216 | 6.3 | 23.1 | 159 | 98 | 5.4 | 1 | 1 | 0 | 1 | 1 | 1 |
| 910 | 1 | 0 | 1 | 56 | 24.8 | 78.5 | 89 | 185 | 36 | 129 | 80 | 7.4 | 38.6 | 112 | 80 | 5.7 | 0 | 0 | 1 | 0 | 0 | 0 |
| 911 | 0 |  | 1 | 36 | 25.3 | 82.5 | 83 | 233 | 56 | 151 | 179 | 8.4 | 26.2 | 116 | 78 | 4.9 | 0 | 1 | 0 | 1 | 0 | 0 |
| 912 | 1 | 0 | 1 | 56 | 24.6 | 84.0 | 82 | 221 | 42 | 152 | 129 | 8.9 | 25.4 | 134 | 88 | 5.3 | 0 | 0 | 0 | 1 | 0 | 0 |
| 913 | 0 |  | 1 | 64 | 25.5 | 87.0 | 80 | 131 | 39 | 73 | 49 | 8.2 | 35.0 | 164 | 94 | 5.5 | 0 | 0 | 1 | 1 | 0 | 0 |
| 914 | 0 |  | 1 | 54 | 25.3 | 92.5 | 93 | 163 | 42 | 97 | 163 | 6.4 | 26.7 | 115 | 71 | 5.4 | 1 | 1 | 0 | 0 | 0 | 0 |
| 915 | 1 | 0 | 1 | 37 | 19.6 | 68.5 | 84 | 185 | 76 | 94 | 52 | 6.8 | 30.2 | 114 | 77 | 5.4 | 0 | 0 | 0 | 0 | 0 | 0 |
| 916 | 0 |  | 1 | 52 | 20.0 | 70.0 | 88 | 157 | 57 | 85 | 36 | 5.7 | 18.9 | 143 | 79 | 5.1 | 0 | 0 | 0 | 1 | 0 | 0 |
| 917 | 0 |  | 1 | 34 | 28.3 | 87.5 | 96 | 229 | 39 | 176 | 90 | 7.5 | 31.5 | 121 | 69 | 5.5 | 0 | 0 | 1 | 0 | 0 | 0 |
| 918 | 0 |  | 0 | 52 | 24.3 | 81.0 | 99 | 271 | 56 | 187 | 113 | 5.9 | 22.5 | 118 | 73 | 5.5 | 1 | 0 | 0 | 0 | 0 | 0 |
| 919 | 0 |  | 1 | 37 | 23.0 | 81.0 | 76 | 164 | 59 | 63 | 110 | 6.0 | 22.6 | 122 | 73 | 5.1 | 0 | 0 | 0 | 0 | 0 | 0 |
| 920 | 1 | 0 | 1 | 63 | 27.8 | 84.0 | 104 | 275 | 45 | 198 | 100 | 6.3 | 35.3 | 110 | 67 | 5.8 | 0 | 0 | 0 | 1 | 1 | 0 |
| 921 | 1 | 1 | 1 | 43 | 26.2 | 88.5 | 86 | 199 | 40 | 137 | 104 | 7.0 | 25.7 | 134 | 95 | 4.6 | 0 | 0 | 0 | 1 | 0 | 0 |
| 922 | 0 |  | 0 | 58 | 22.7 | 74.5 | 107 | 213 | 43 | 123 | 181 | 5.5 | 29.7 | 115 | 56 | 6.0 | 0 | 1 | 1 | 1 | 1 | 1 |
| 923 | 0 |  | 1 | 31 | 31.1 | 96.0 | 80 | 207 | 30 | 132 | 319 | 7.9 | 19.5 | 111 | 65 | 5.6 | 1 | 1 | 1 | 0 | 0 | 1 |
| 924 | 1 | 0 | 1 | 30 | 27.6 | 89.0 | 87 | 165 | 45 | 100 | 123 | 7.1 | 25.3 | 120 | 80 | 5.5 | 0 | 0 | 0 | 0 | 0 | 0 |
| 925 | 1 | 1 | 0 | 36 | 25.3 | 91.5 | 79 | 217 | 73 | 167 | 80 | 4.0 | 18.5 | 105 | 68 | 5.3 | 1 | 0 | 0 | 0 | 0 | 0 |
| 926 | 0 |  | 0 | 35 | 23.2 | 74.0 | 69 | 218 | 58 | 143 | 37 | 4.6 | 43.8 | 106 | 62 | 4.6 | 0 | 0 | 0 | 0 | 0 | 0 |
| 927 | 0 |  | 0 | 30 | 19.8 | 68.0 | 93 | 202 | 69 | 122 | 100 | 4.0 | 19.4 | 108 | 71 | 5.2 | 0 | 0 | 0 | 0 | 0 | 0 |
| 928 | 0 |  | 0 | 40 | 30.3 | 86.0 | 87 | 247 | 68 | 161 | 71 | 4.4 | 29.4 | 143 | 87 | 4.9 | 1 | 0 | 0 | 1 | 0 | 0 |
| 929 | 0 |  | 1 | 58 | 23.3 | 80.0 | 95 | 152 | 41 | 98 | 92 | 6.7 | 51.9 | 114 | 64 | 4.9 | 0 | 0 | 0 | 0 | 0 | 0 |
| 930 | 0 |  | 1 | 52 | 27.7 | 92.0 | 86 | 201 | 39 | 159 | 66 | 7.2 | 24.3 | 109 | 75 | 5.3 | 1 | 0 | 1 | 0 | 0 | 0 |
| 931 | 1 | 0 | 1 | 43 | 26.2 | 78.0 | 80 | 205 | 30 | 150 | 139 | 5.2 | 25.2 | 127 | 79 | 4.1 | 0 | 0 | 1 | 0 | 0 | 0 |
| 932 | 0 |  | 1 | 36 | 21.3 | 74.0 | 77 | 128 | 45 | 76 | 43 | 8.5 | 20.5 | 114 | 64 | 5.9 | 0 | 0 | 0 | 0 | 1 | 0 |
| 933 | 1 | 0 | 1 | 53 | 26.4 | 89.5 | 92 | 201 | 36 | 133 | 86 | 1.3 | 18.8 | 119 | 76 | 4.8 | 0 | 0 | 1 | 0 | 0 | 0 |
| 934 | 1 | 1 | 1 | 60 | 31.0 | 98.5 | 94 | 136 | 38 | 76 | 215 | 6.2 | 31.7 | 149 | 89 | 5.8 | 1 | 1 | 1 | 1 | 1 | 1 |
| 935 | 0 |  | 1 | 36 | 28.2 | 99.0 | 104 | 239 | 42 | 172 | 166 | 7.2 | 31.3 | 119 | 68 | 5.4 | 1 | 1 | 0 | 0 | 1 | 1 |
| 936 | 0 |  | 1 | 38 | 22.6 | 77.0 | 97 | 198 | 43 | 139 | 86 | 6.5 | 16.2 | 128 | 89 | 5.3 | 0 | 0 | 0 | 1 | 0 | 0 |
| 937 | 1 | 0 | 1 | 53 | 23.8 | 82.0 | 105 | 257 | 38 | 167 | 142 | 7.6 | 26.7 | 128 | 83 | 5.3 | 0 | 0 | 1 | 0 | 1 | 0 |
| 938 | 1 | 0 | 1 | 64 | 25.2 | 87.0 | 140 | 120 | 43 | 68 | 51 | 6.1 | 26.8 | 132 | 80 | 6.0 | 0 | 0 | 0 | 1 | 1 | 0 |
| 939 | 1 | 1 | 1 | 63 | 23.1 | 80.0 | 95 | 284 | 36 | 144 | 275 | 5.0 | 24.7 | 122 | 73 | 5.7 | 0 | 1 | 1 | 0 | 0 | 0 |
| 940 | 0 |  | 0 | 59 | 21.7 | 76.0 | 92 | 235 | 58 | 166 | 58 | 5.8 | 34.7 | 133 | 70 | 5.5 | 0 | 0 | 0 | 1 | 0 | 0 |
| 941 | 0 |  | 1 | 59 | 24.0 | 83.0 | 73 | 186 | 63 | 103 | 47 | 5.9 | 28.1 | 144 | 84 | 5.4 | 0 | 0 | 0 | 1 | 0 | 0 |
| 942 | 1 | 1 | 1 | 45 | 28.6 | 96.0 | 143 | 158 | 49 | 71 | 253 | 7.0 | 23.3 | 115 | 80 | 6.2 | 1 | 1 | 0 | 0 | 1 | 1 |
| 943 | 0 |  | 1 | 31 | 23.9 | 81.0 | 91 | 189 | 51 | 125 | 78 | 5.8 | 26.0 | 102 | 52 | 5.4 | 0 | 0 | 0 | 0 | 0 | 0 |
| 944 | 0 |  | 1 | 47 | 21.0 | 79.0 | 93 | 205 | 36 | 150 | 108 | 6.0 | 35.8 | 127 | 98 | 5.0 | 0 | 0 | 1 | 1 | 0 | 0 |
| 945 | 1 | 1 | 1 | 54 | 26.0 | 90.0 | 101 | 233 | 51 | 159 | 99 | 6.6 | 34.4 | 129 | 83 | 5.5 | 1 | 0 | 0 | 0 | 1 | 0 |
| 946 | 0 |  | 0 | 40 | 21.5 | 80.0 | 90 | 165 | 46 | 111 | 49 | 4.2 | 23.6 | 94 | 60 | 5.3 | 1 | 0 | 1 | 1 | 0 | 1 |
| 947 | 0 |  | 0 | 44 | 18.9 | 68.0 | 82 | 130 | 46 | 72 | 45 | 4.2 | 29.7 | 105 | 57 | 5.0 | 0 | 0 | 1 | 0 | 0 | 0 |
| 948 | 1 | 1 | 1 | 55 | 25.9 | 82.5 | 81 | 177 | 77 | 87 | 60 | 4.9 | 22.4 | 127 | 78 | 5.6 | 0 | 0 | 0 | 0 | 0 | 0 |
| 949 | 1 | 1 | 1 | 61 | 27.4 | 92.0 | 102 | 185 | 42 | 103 | 213 | 7.9 | 52.1 | 135 | 86 | 6.3 | 1 | 1 | 0 | 1 | 1 | 1 |
| 950 | 0 |  | 1 | 36 | 23.4 | 82.0 | 93 | 156 | 29 | 102 | 118 | 6.5 | 32.1 | 134 | 81 | 5.0 | 0 | 0 | 1 | 1 | 0 | 0 |
| 951 | 1 | 1 | 1 | 42 | 21.6 | 79.5 | 89 | 235 | 35 | 164 | 145 | 6.3 | 28.5 | 103 | 71 | 5.3 | 0 | 0 | 1 | 0 | 0 | 0 |
| 952 | 0 |  | 0 | 39 | 18.6 | 63.5 | 83 | 191 | 75 | 101 | 58 | 5.0 | 18.0 | 103 | 72 | 4.7 | 0 | 0 | 0 | 0 | 0 | 0 |
| 953 | 0 |  | 0 | 45 | 24.1 | 74.0 | 78 | 205 | 37 | 157 | 40 | 4.7 | 20.0 | 119 | 92 | 5.3 | 0 | 0 | 1 | 1 | 0 | 0 |
| 954 | 0 |  | 0 | 39 | 20.1 | 69.0 | 91 | 251 | 99 | 140 | 34 | 4.6 | 29.3 | 100 | 57 | 5.1 | 0 | 0 | 0 | 0 | 0 | 0 |
| 955 | 0 |  | 0 | 35 | 20.1 | 67.0 | 82 | 193 | 78 | 108 | 42 | 5.5 | 29.3 | 128 | 78 | 5.4 | 0 | 0 | 0 | 0 | 0 | 0 |
| 956 | 0 |  | 1 | 60 | 23.1 | 87.5 | 101 | 191 | 36 | 116 | 195 | 8.4 | 41.8 | 122 | 81 | 5.4 | 0 | 1 | 1 | 0 | 1 | 1 |
| 957 | 0 |  | 0 | 37 | 20.4 | 65.0 | 81 | 241 | 78 | 144 | 65 | 3.7 | 32.6 | 96 | 64 | 5.0 | 0 | 0 | 0 | 1 | 0 | 0 |
| 958 | 0 |  | 1 | 49 | 27.2 | 91.0 | 98 | 208 | 55 | 135 | 86 | 5.9 | 20.0 | 117 | 76 | 5.7 | 1 | 0 | 0 | 0 | 0 | 0 |
| 959 | 0 |  | 0 | 34 | 15.9 | 60.0 | 88 | 177 | 81 | 78 | 87 | 3.2 | 21.6 | 112 | 77 | 5.1 | 0 | 0 | 0 | 0 | 0 | 0 |
| 960 | 1 | 0 | 1 | 40 | 23.4 | 78.0 | 93 | 210 | 53 | 146 | 106 | 5.6 | 38.0 | 124 | 72 | 5.0 | 0 | 0 | 0 | 0 | 0 | 0 |
| 961 | 0 |  | 0 | 38 | 19.5 | 67.0 | 76 | 229 | 59 | 165 | 63 | 5.4 | 25.4 | 99 | 60 | 5.3 | 0 | 0 | 0 | 1 | 0 | 0 |
| 962 | 1 | 1 | 0 | 39 | 18.4 | 64.0 | 78 | 210 | 64 | 137 | 51 | 4.4 | 24.6 | 107 | 67 | 5.6 | 0 | 0 | 0 | 0 | 0 | 0 |
| 963 | 0 |  | 1 | 31 | 37.0 | 118.0 | 99 | 211 | 36 | 157 | 195 | 8.9 | 19.7 | 139 | 84 | 5.3 | 1 | 1 | 1 | 1 | 0 | 1 |
| 964 | 1 | 1 | 1 | 54 | 26.9 | 85.5 | 99 | 332 | 56 | 250 | 108 | 8.7 | 43.4 | 142 | 102 | 5.5 | 0 | 0 | 0 | 1 | 0 | 0 |
| 965 | 0 |  | 0 | 62 | 21.4 | 73.0 | 98 | 131 | 53 | 66 | 38 | 3.1 | 29.1 | 133 | 60 | 5.1 | 0 | 0 | 0 | 1 | 0 | 0 |
| 966 | 0 |  | 0 | 51 | 38.2 | 100.0 | 91 | 226 | 53 | 152 | 111 | 6.6 | 13.6 | 156 | 100 | 5.8 | 1 | 0 | 0 | 1 | 1 | 1 |
| 967 | 1 | 1 | 1 | 49 | 25.9 | 89.0 | 101 | 206 | 46 | 134 | 167 | 6.9 | 24.8 | 143 | 94 | 5.7 | 0 | 1 | 0 | 1 | 1 | 1 |
| 968 | 1 | 1 | 1 | 62 | 25.7 | 92.5 | 115 | 229 | 32 | 156 | 284 | 6.3 | 31.0 | 143 | 88 | 6.3 | 1 | 1 | 1 | 1 | 1 | 1 |
| 969 | 1 | 0 | 0 | 62 | 24.3 | 82.0 | 90 | 239 | 68 | 144 | 151 | 4.6 | 10.7 | 112 | 65 | 5.3 | 1 | 1 | 0 | 0 | 0 | 0 |
| 970 | 1 | 0 | 1 | 55 | 27.1 | 85.0 | 268 | 176 | 39 | 117 | 154 | 5.1 | 16.5 | 117 | 70 | 10.7 | 0 | 1 | 1 | 1 | 1 | 1 |
| 971 | 0 |  | 1 | 37 | 26.1 | 92.0 | 71 | 201 | 43 | 157 | 63 | 7.9 | 29.4 | 142 | 94 | 5.3 | 1 | 0 | 0 | 1 | 0 | 0 |
| 972 | 1 | 1 | 0 | 64 | 21.6 | 85.0 | 88 | 235 | 50 | 166 | 90 | 4.1 | 37.0 | 106 | 68 | 5.4 | 1 | 0 | 0 | 0 | 0 | 0 |
| 973 | 0 |  | 1 | 46 | 24.3 | 79.0 | 99 | 188 | 47 | 119 | 132 | 5.4 | 32.0 | 113 | 75 | 5.8 | 0 | 0 | 0 | 0 | 1 | 0 |
| 974 | 0 |  | 0 | 48 | 20.3 | 66.0 | 92 | 167 | 53 | 103 | 49 | 4.6 | 32.5 | 113 | 73 | 5.1 | 0 | 0 | 0 | 0 | 0 | 0 |
| 975 | 1 | 1 | 1 | 50 | 35.9 | 111.0 | 109 | 192 | 56 | 105 | 172 | 7.1 | 27.2 | 133 | 90 | 5.4 | 1 | 1 | 0 | 1 | 1 | 1 |
| 976 | 0 |  | 0 | 37 | 21.7 | 67.0 | 70 | 159 | 64 | 84 | 56 | 6.2 | 25.4 | 106 | 64 | 4.9 | 0 | 0 | 0 | 0 | 0 | 0 |
| 977 | 1 | 0 | 0 | 30 | 23.4 | 76.0 | 88 | 150 | 31 | 106 | 68 | 3.2 | 23.3 | 115 | 74 | 5.1 | 0 | 0 | 1 | 0 | 0 | 0 |
| 978 | 0 |  | 0 | 53 | 25.3 | 78.0 | 83 | 178 | 57 | 110 | 44 | 4.4 | 13.7 | 137 | 71 | 4.9 | 0 | 0 | 0 | 1 | 0 | 0 |
| 979 | 0 |  | 0 | 56 | 24.7 | 79.0 | 87 | 213 | 69 | 135 | 92 | 3.3 | 17.0 | 117 | 61 | 5.4 | 0 | 0 | 0 | 0 | 0 | 0 |
| 980 | 1 | 0 | 1 | 59 | 25.3 | 82.0 | 105 | 177 | 48 | 118 | 61 | 5.9 | 28.4 | 128 | 75 | 6.2 | 0 | 0 | 0 | 0 | 1 | 0 |
| 981 | 0 |  | 1 | 32 | 23.9 | 79.0 | 90 | 147 | 32 | 84 | 229 | 6.1 | 23.7 | 124 | 74 | 5.1 | 0 | 1 | 1 | 0 | 0 | 0 |
| 982 | 1 | 1 | 0 | 60 | 19.1 | 65.0 | 96 | 191 | 64 | 107 | 42 | 5.1 | 17.8 | 117 | 70 | 5.4 | 0 | 0 | 0 | 0 | 0 | 0 |
| 983 | 0 |  | 1 | 32 | 28.1 | 90.0 | 89 | 190 | 33 | 78 | 576 | 7.0 | 23.9 | 126 | 73 | 5.3 | 1 | 1 | 1 | 0 | 0 | 1 |
| 984 | 0 |  | 1 | 37 | 28.8 | 86.0 | 82 | 147 | 42 | 53 | 245 | 6.4 | 27.4 | 124 | 89 | 5.0 | 0 | 1 | 0 | 1 | 0 | 0 |
| 985 | 1 | 1 | 1 | 58 | 29.9 | 102.0 | 111 | 225 | 45 | 146 | 221 | 7.5 | 37.4 | 127 | 80 | 5.7 | 1 | 1 | 0 | 0 | 1 | 1 |
| 986 | 0 |  | 1 | 39 | 22.5 | 77.5 | 78 | 201 | 60 | 130 | 51 | 6.3 | 29.0 | 117 | 76 | 4.8 | 0 | 0 | 0 | 0 | 0 | 0 |
| 987 | 1 | 0 | 1 | 38 | 25.0 | 82.0 | 87 | 202 | 79 | 103 | 97 | 7.4 | 34.4 | 138 | 83 | 5.2 | 0 | 0 | 0 | 1 | 0 | 0 |
| 988 | 1 | 0 | 1 | 64 | 24.3 | 80.0 | 107 | 195 | 48 | 119 | 168 | 6.8 | 12.8 | 117 | 88 | 5.7 | 0 | 1 | 0 | 1 | 1 | 1 |
| 989 | 0 |  | 1 | 63 | 22.9 | 72.0 | 155 | 220 | 53 | 144 | 62 | 4.2 | 41.1 | 141 | 73 | 6.9 | 0 | 0 | 0 | 1 | 1 | 0 |
| 990 | 0 |  | 1 | 38 | 26.4 | 91.0 | 93 | 228 | 54 | 153 | 125 | 5.9 | 38.3 | 114 | 76 | 5.5 | 1 | 0 | 0 | 0 | 0 | 0 |
| 991 | 0 |  | 1 | 42 | 24.3 | 77.5 | 94 | 190 | 40 | 123 | 139 | 6.9 | 30.1 | 148 | 89 | 5.4 | 0 | 0 | 0 | 1 | 0 | 0 |
| 992 | 1 | 0 | 1 | 41 | 27.5 | 87.5 | 84 | 164 | 22 | 78 | 357 | 7.0 | 34.4 | 121 | 83 | 5.2 | 0 | 1 | 1 | 0 | 0 | 0 |
| 993 | 0 |  | 1 | 47 | 26.6 | 89.0 | 101 | 179 | 36 | 101 | 138 | 5.6 | 28.3 | 116 | 69 | 5.5 | 0 | 0 | 1 | 1 | 1 | 1 |
| 994 | 1 | 0 | 1 | 37 | 29.3 | 94.0 | 96 | 295 | 36 | 236 | 197 | 7.0 | 21.9 | 134 | 83 | 5.5 | 1 | 1 | 1 | 1 | 0 | 1 |
| 995 | 1 | 1 | 1 | 55 | 25.8 | 87.0 | 97 | 193 | 33 | 138 | 95 | 7.5 | 24.8 | 120 | 72 | 5.3 | 0 | 0 | 1 | 0 | 0 | 0 |
| 996 | 0 |  | 0 | 60 | 24.1 | 79.0 | 85 | 203 | 54 | 125 | 71 | 4.7 | 31.9 | 123 | 71 | 5.6 | 0 | 0 | 0 | 0 | 0 | 0 |
| 997 | 0 |  | 1 | 47 | 21.5 | 77.0 | 93 | 248 | 67 | 160 | 222 | 3.9 | 32.9 | 126 | 87 | 5.4 | 0 | 1 | 0 | 1 | 0 | 0 |
| 998 | 0 |  | 1 | 48 | 20.0 | 66.0 | 78 | 227 | 78 | 132 | 35 | 5.7 | 44.9 | 117 | 63 | 4.9 | 0 | 0 | 0 | 0 | 0 | 0 |
| 999 | 1 | 1 | 0 | 54 | 21.5 | 70.0 | 78 | 182 | 80 | 86 | 37 | 4.3 | 17.4 | 121 | 68 | 5.3 | 0 | 0 | 0 | 0 | 0 | 0 |
| 1000 | 1 | 0 | 1 | 57 | 23.2 | 88.0 | 96 | 236 | 56 | 156 | 64 | 5.7 | 62.0 | 133 | 94 | 5.3 | 0 | 0 | 0 | 1 | 0 | 0 |
| 1001 | 1 | 1 | 1 | 43 | 30.5 | 90.0 | 87 | 228 | 24 | 153 | 273 | 5.8 | 45.8 | 132 | 88 | 5.4 | 1 | 1 | 1 | 1 | 0 | 1 |
| 1002 | 1 | 1 | 1 | 59 | 31.8 | 103.0 | 103 | 197 | 32 | 135 | 218 | 9.4 | 13.2 | 116 | 75 | 5.9 | 1 | 1 | 1 | 0 | 1 | 1 |
| 1003 | 0 |  | 1 | 47 | 22.9 | 82.0 | 88 | 190 | 44 | 130 | 95 | 6.8 | 26.1 | 105 | 72 | 5.1 | 0 | 0 | 0 | 0 | 0 | 0 |
| 1004 | 1 | 0 | 0 | 60 | 34.1 | 104.0 | 177 | 302 | 74 | 219 | 101 | 5.7 | 30.5 | 155 | 83 | 6.8 | 1 | 0 | 0 | 1 | 1 | 1 |
| 1005 | 0 |  | 0 | 43 | 22.0 | 70.0 | 85 | 215 | 61 | 144 | 62 | 4.3 | 28.4 | 118 | 65 | 5.4 | 0 | 0 | 0 | 0 | 0 | 0 |
| 1006 | 0 |  | 0 | 46 | 17.8 | 64.0 | 92 | 195 | 51 | 125 | 88 | 3.7 | 14.8 | 119 | 73 | 4.9 | 0 | 0 | 0 | 0 | 0 | 0 |
| 1007 | 1 | 1 | 1 | 46 | 26.2 | 91.0 | 89 | 207 | 60 | 113 | 179 | 5.9 | 22.1 | 144 | 93 | 5.3 | 1 | 1 | 0 | 1 | 0 | 1 |
| 1008 | 0 |  | 1 | 33 | 26.2 | 85.0 | 81 | 180 | 39 | 123 | 94 | 8.0 | 19.0 | 130 | 89 | 3.9 | 0 | 0 | 1 | 1 | 0 | 0 |
| 1009 | 1 | 0 | 1 | 44 | 30.2 | 104.0 | 97 | 228 | 44 | 155 | 103 | 5.8 | 34.9 | 112 | 75 | 5.5 | 1 | 0 | 0 | 0 | 0 | 0 |
| 1010 | 0 |  | 1 | 39 | 21.6 | 77.0 | 98 | 170 | 44 | 112 | 69 | 4.1 | 27.9 | 119 | 82 | 5.0 | 0 | 0 | 0 | 0 | 0 | 0 |
| 1011 | 1 | 1 | 0 | 46 | 23.3 | 67.0 | 94 | 231 | 61 | 145 | 113 | 4.4 | 25.7 | 109 | 70 | 4.2 | 0 | 0 | 0 | 0 | 0 | 0 |
| 1012 | 0 |  | 0 | 47 | 23.8 | 73.5 | 83 | 144 | 58 | 87 | 58 | 2.7 | 17.0 | 124 | 72 | 4.5 | 0 | 0 | 0 | 0 | 0 | 0 |
| 1013 | 0 |  | 1 | 39 | 27.2 | 90.0 | 85 | 197 | 38 | 107 | 171 | 6.1 | 24.2 | 122 | 73 | 5.6 | 1 | 1 | 1 | 0 | 0 | 1 |
| 1014 | 1 | 1 | 1 | 55 | 21.7 | 80.0 | 78 | 196 | 58 | 106 | 136 | 7.1 | 40.7 | 123 | 90 | 4.7 | 0 | 0 | 0 | 1 | 0 | 0 |
| 1015 | 1 | 0 | 0 | 55 | 22.8 | 70.0 | 93 | 207 | 52 | 132 | 95 | 2.9 | 28.3 | 136 | 75 | 5.0 | 0 | 0 | 0 | 1 | 0 | 0 |
| 1016 | 0 |  | 0 | 32 | 19.8 | 65.0 | 90 | 283 | 71 | 192 | 63 | 3.7 | 25.8 | 94 | 67 | 5.3 | 0 | 0 | 0 | 1 | 0 | 0 |
| 1017 | 0 |  | 0 | 58 | 30.6 | 91.5 | 119 | 133 | 38 | 79 | 123 | 4.3 | 19.1 | 120 | 70 | 6.9 | 1 | 0 | 1 | 1 | 1 | 1 |
| 1018 | 0 |  | 1 | 42 | 22.0 | 70.5 | 89 | 169 | 76 | 72 | 64 | 5.5 | 35.6 | 111 | 79 | 5.5 | 0 | 0 | 0 | 0 | 0 | 0 |
| 1019 | 0 |  | 1 | 34 | 27.3 | 86.0 | 92 | 203 | 38 | 149 | 65 | 7.1 | 17.9 | 135 | 86 | 5.1 | 0 | 0 | 1 | 1 | 0 | 0 |
| 1020 | 0 |  | 1 | 30 | 31.4 | 100.0 | 102 | 182 | 36 | 128 | 127 | 5.7 | 61.7 | 126 | 69 | 5.6 | 1 | 0 | 1 | 0 | 1 | 1 |
| 1021 | 0 |  | 0 | 30 | 18.9 | 64.0 | 85 | 184 | 62 | 109 | 70 | 4.4 | 29.3 | 98 | 53 | 5.0 | 0 | 0 | 0 | 1 | 0 | 0 |
| 1022 | 0 |  | 0 | 64 | 20.5 | 76.5 | 221 | 242 | 76 | 149 | 107 | 4.9 | 47.0 | 113 | 60 | 7.3 | 0 | 0 | 0 | 0 | 1 | 0 |
| 1023 | 0 |  | 1 | 42 | 23.0 | 88.0 | 101 | 202 | 64 | 115 | 50 | 5.2 | 33.7 | 118 | 82 | 5.4 | 0 | 0 | 0 | 0 | 1 | 0 |
| 1024 | 0 |  | 1 | 37 | 20.7 | 66.0 | 90 | 200 | 67 | 132 | 69 | 7.2 | 39.8 | 127 | 72 | 5.1 | 0 | 0 | 0 | 0 | 0 | 0 |
| 1025 | 1 | 1 | 0 | 33 | 20.7 | 85.5 | 96 | 152 | 57 | 77 | 61 | 4.4 | 18.9 | 103 | 62 | 5.1 | 1 | 0 | 0 | 0 | 0 | 0 |
| 1026 | 0 |  | 1 | 33 | 23.5 | 80.0 | 89 | 213 | 53 | 145 | 112 | 6.1 | 28.0 | 138 | 79 | 5.1 | 0 | 0 | 0 | 1 | 0 | 0 |
| 1027 | 1 | 0 | 1 | 50 | 30.2 | 91.0 | 104 | 172 | 32 | 101 | 107 | 6.1 | 28.9 | 145 | 103 | 5.6 | 1 | 0 | 1 | 1 | 1 | 1 |
| 1028 | 1 | 1 | 0 | 49 | 23.8 | 75.0 | 93 | 208 | 46 | 138 | 100 | 5.0 | 36.7 | 114 | 75 | 5.7 | 0 | 0 | 1 | 0 | 0 | 0 |
| 1029 | 1 | 1 | 1 | 62 | 24.6 | 77.0 | 93 | 167 | 27 | 107 | 143 | 6.5 | 23.1 | 118 | 80 | 5.7 | 0 | 0 | 1 | 1 | 0 | 0 |
| 1030 | 0 |  | 1 | 31 | 20.4 | 67.0 | 86 | 164 | 34 | 101 | 131 | 6.0 | 32.9 | 117 | 78 | 5.2 | 0 | 0 | 1 | 0 | 0 | 0 |
| 1031 | 0 |  | 1 | 58 | 29.4 | 106.5 | 93 | 204 | 42 | 139 | 108 | 6.4 | 36.2 | 157 | 101 | 5.4 | 1 | 0 | 0 | 1 | 0 | 0 |
| 1032 | 1 | 1 | 1 | 53 | 22.6 | 82.0 | 94 | 199 | 55 | 105 | 143 | 7.0 | 33.8 | 124 | 86 | 4.9 | 0 | 0 | 0 | 1 | 0 | 0 |
| 1033 | 0 |  | 1 | 62 | 22.7 | 77.0 | 238 | 263 | 57 | 189 | 85 | 4.3 | 43.7 | 151 | 83 | 11.5 | 0 | 0 | 0 | 1 | 1 | 0 |
| 1034 | 1 | 0 | 1 | 35 | 33.4 | 109.0 | 89 | 238 | 31 | 146 | 304 | 7.6 | 24.1 | 123 | 87 | 5.4 | 1 | 1 | 1 | 1 | 0 | 1 |
| 1035 | 1 | 1 | 1 | 57 | 23.3 | 80.0 | 86 | 235 | 54 | 166 | 83 | 8.2 | 27.6 | 111 | 72 | 5.6 | 0 | 0 | 0 | 0 | 0 | 0 |
| 1036 | 0 |  | 1 | 42 | 22.5 | 83.0 | 90 | 267 | 58 | 181 | 322 | 7.1 | 40.7 | 112 | 69 | 5.3 | 0 | 1 | 0 | 0 | 0 | 0 |
| 1037 | 0 |  | 1 | 43 | 27.7 | 93.0 | 96 | 190 | 39 | 130 | 185 | 9.4 | 22.4 | 123 | 88 | 5.4 | 1 | 1 | 1 | 1 | 0 | 1 |
| 1038 | 0 |  | 1 | 48 | 25.1 | 84.0 | 98 | 198 | 35 | 141 | 95 | 6.5 | 28.4 | 116 | 73 | 5.3 | 0 | 0 | 1 | 0 | 0 | 0 |
| 1039 | 0 |  | 1 | 54 | 26.9 | 88.0 | 117 | 160 | 78 | 61 | 87 | 6.0 | 36.9 | 150 | 95 | 8.8 | 0 | 0 | 0 | 1 | 1 | 0 |
| 1040 | 1 | 1 | 1 | 37 | 31.8 | 95.0 | 94 | 174 | 49 | 107 | 76 | 7.6 | 19.7 | 132 | 86 | 4.6 | 1 | 0 | 0 | 1 | 0 | 0 |
| 1041 | 0 |  | 0 | 54 | 24.3 | 84.0 | 90 | 235 | 51 | 154 | 94 | 6.8 | 28.1 | 109 | 64 | 5.1 | 1 | 0 | 0 | 0 | 0 | 0 |
| 1042 | 1 | 0 | 0 | 40 | 25.4 | 79.0 | 101 | 229 | 60 | 145 | 144 | 4.6 | 26.4 | 106 | 77 | 5.1 | 0 | 0 | 0 | 0 | 1 | 0 |
| 1043 | 0 |  | 1 | 52 | 21.5 | 68.0 | 102 | 214 | 58 | 150 | 62 | 5.4 | 47.9 | 127 | 74 | 5.6 | 0 | 0 | 0 | 0 | 1 | 0 |
| 1044 | 1 | 1 | 1 | 53 | 28.4 | 95.0 | 120 | 204 | 41 | 116 | 302 | 7.0 | 13.7 | 105 | 77 | 5.8 | 1 | 1 | 0 | 1 | 1 | 1 |
| 1045 | 0 |  | 0 | 37 | 20.7 | 63.5 | 78 | 179 | 62 | 101 | 41 | 4.5 | 14.4 | 96 | 70 | 4.7 | 0 | 0 | 0 | 1 | 0 | 0 |
| 1046 | 0 |  | 1 | 39 | 25.7 | 78.0 | 90 | 185 | 45 | 131 | 50 | 8.3 | 37.4 | 130 | 74 | 5.4 | 0 | 0 | 0 | 1 | 0 | 0 |
| 1047 | 0 |  | 0 | 62 | 26.5 | 93.0 | 95 | 308 | 48 | 220 | 136 | 8.1 | 24.2 | 122 | 74 | 5.5 | 1 | 0 | 1 | 1 | 0 | 1 |
| 1048 | 0 |  | 1 | 52 | 26.5 | 95.0 | 93 | 203 | 83 | 96 | 104 | 7.3 | 30.1 | 116 | 84 | 5.5 | 1 | 0 | 0 | 1 | 0 | 0 |
| 1049 | 1 | 1 | 1 | 48 | 25.4 | 77.0 | 91 | 228 | 50 | 169 | 70 | 10.5 | 37.4 | 127 | 89 | 5.8 | 0 | 0 | 0 | 1 | 1 | 0 |
| 1050 | 0 |  | 1 | 46 | 29.2 | 95.0 | 90 | 157 | 48 | 76 | 199 | 6.1 | 40.6 | 108 | 71 | 5.3 | 1 | 1 | 0 | 1 | 0 | 1 |
| 1051 | 1 | 0 | 0 | 61 | 24.8 | 76.0 | 94 | 254 | 48 | 176 | 68 | 5.8 | 32.2 | 121 | 67 | 5.3 | 0 | 0 | 1 | 0 | 0 | 0 |
| 1052 | 0 |  | 1 | 33 | 27.6 | 93.5 | 88 | 213 | 33 | 144 | 197 | 7.4 | 48.7 | 122 | 76 | 5.5 | 1 | 1 | 1 | 0 | 0 | 1 |
| 1053 | 0 |  | 0 | 43 | 25.2 | 79.5 | 93 | 212 | 42 | 145 | 67 | 5.4 | 30.5 | 125 | 77 | 4.9 | 0 | 0 | 1 | 0 | 0 | 0 |
| 1054 | 1 | 0 | 1 | 30 | 24.9 | 86.0 | 93 | 237 | 32 | 169 | 158 | 7.0 | 16.4 | 117 | 78 | 5.4 | 0 | 1 | 1 | 0 | 0 | 0 |
| 1055 | 0 |  | 0 | 37 | 22.1 | 72.0 | 80 | 197 | 77 | 108 | 63 | 5.8 | 17.1 | 116 | 77 | 5.2 | 0 | 0 | 0 | 0 | 0 | 0 |
| 1056 | 0 |  | 0 | 50 | 22.7 | 80.0 | 94 | 199 | 43 | 125 | 124 | 4.1 | 13.2 | 101 | 65 | 5.3 | 1 | 0 | 1 | 0 | 0 | 0 |
| 1057 | 0 |  | 0 | 53 | 23.5 | 66.0 | 92 | 227 | 59 | 139 | 115 | 4.6 | 32.6 | 99 | 67 | 5.5 | 0 | 0 | 0 | 1 | 0 | 0 |
| 1058 | 0 |  | 0 | 43 | 20.8 | 71.0 | 84 | 159 | 53 | 89 | 99 | 3.9 | 30.7 | 111 | 70 | 4.8 | 0 | 0 | 0 | 0 | 0 | 0 |
| 1059 | 1 | 1 | 1 | 43 | 23.4 | 80.0 | 99 | 182 | 44 | 117 | 95 | 6.4 | 33.3 | 112 | 75 | 5.4 | 0 | 0 | 0 | 0 | 0 | 0 |
| 1060 | 0 |  | 0 | 51 | 22.9 | 76.0 | 95 | 326 | 52 | 250 | 119 | 5.3 | 11.8 | 107 | 67 | 5.4 | 0 | 0 | 0 | 0 | 0 | 0 |
| 1061 | 1 | 0 | 1 | 58 | 24.0 | 85.0 | 92 | 184 | 54 | 105 | 128 | 6.7 | 28.2 | 141 | 86 | 5.3 | 0 | 0 | 0 | 1 | 0 | 0 |
| 1062 | 0 |  | 0 | 36 | 21.6 | 78.0 | 82 | 209 | 51 | 139 | 51 | 4.8 | 28.3 | 100 | 67 | 5.3 | 0 | 0 | 0 | 0 | 0 | 0 |
| 1063 | 1 | 1 | 1 | 37 | 24.3 | 80.0 | 83 | 193 | 34 | 146 | 80 | 6.2 | 23.7 | 115 | 76 | 5.4 | 0 | 0 | 1 | 0 | 0 | 0 |
| 1064 | 1 | 1 | 0 | 35 | 21.7 | 72.0 | 75 | 187 | 68 | 114 | 80 | 3.3 | 8.6 | 109 | 76 | 5.0 | 0 | 0 | 0 | 0 | 0 | 0 |
| 1065 | 1 | 0 | 1 | 46 | 24.8 | 86.0 | 101 | 177 | 33 | 119 | 166 | 7.0 | 26.5 | 114 | 81 | 5.5 | 0 | 1 | 1 | 0 | 1 | 1 |
| 1066 | 1 | 1 | 1 | 60 | 26.0 | 87.0 | 106 | 177 | 36 | 114 | 132 | 8.8 | 36.2 | 148 | 93 | 5.5 | 0 | 0 | 1 | 1 | 1 | 1 |
| 1067 | 0 |  | 1 | 59 | 26.8 | 87.0 | 99 | 178 | 32 | 98 | 245 | 6.4 | 35.0 | 139 | 93 | 4.7 | 0 | 1 | 1 | 1 | 0 | 1 |
| 1068 | 0 |  | 0 | 33 | 17.8 | 60.0 | 85 | 224 | 94 | 110 | 58 | 5.3 | 17.1 | 95 | 60 | 4.6 | 0 | 0 | 0 | 1 | 0 | 0 |
| 1069 | 0 |  | 1 | 36 | 28.8 | 92.0 | 98 | 216 | 44 | 142 | 286 | 7.8 | 34.1 | 111 | 70 | 5.3 | 1 | 1 | 0 | 0 | 0 | 0 |
| 1070 | 0 |  | 1 | 51 | 24.4 | 85.0 | 88 | 232 | 42 | 161 | 168 | 6.1 | 21.4 | 105 | 71 | 5.1 | 0 | 1 | 0 | 0 | 0 | 0 |
| 1071 | 1 | 0 | 0 | 39 | 22.0 | 76.5 | 77 | 180 | 45 | 124 | 58 | 3.8 | 22.5 | 103 | 64 | 5.2 | 0 | 0 | 1 | 0 | 0 | 0 |
| 1072 | 1 | 1 | 1 | 58 | 19.1 | 72.0 | 101 | 204 | 26 | 153 | 119 | 7.1 | 51.2 | 136 | 68 | 5.8 | 0 | 0 | 1 | 1 | 1 | 1 |
| 1073 | 0 |  | 0 | 64 | 25.5 | 80.0 | 101 | 280 | 68 | 206 | 67 | 5.8 | 34.3 | 110 | 69 | 5.5 | 1 | 0 | 0 | 0 | 1 | 0 |
| 1074 | 1 | 1 | 1 | 40 | 19.3 | 67.0 | 89 | 167 | 71 | 82 | 74 | 5.7 | 26.6 | 109 | 65 | 5.0 | 0 | 0 | 0 | 0 | 0 | 0 |
| 1075 | 1 | 1 | 1 | 61 | 23.0 | 78.0 | 99 | 186 | 36 | 130 | 121 | 4.3 | 16.2 | 124 | 78 | 5.3 | 0 | 0 | 1 | 0 | 0 | 0 |
| 1076 | 0 |  | 0 | 50 | 26.4 | 81.0 | 105 | 233 | 56 | 146 | 127 | 5.7 | 31.1 | 122 | 81 | 6.0 | 1 | 0 | 0 | 0 | 1 | 0 |
| 1077 | 0 |  | 1 | 56 | 24.8 | 90.5 | 104 | 273 | 42 | 189 | 181 | 6.1 | 53.0 | 115 | 76 | 5.9 | 1 | 1 | 0 | 0 | 1 | 1 |
| 1078 | 1 | 1 | 0 | 64 | 24.0 | 74.0 | 73 | 198 | 68 | 127 | 45 | 5.7 | 48.0 | 111 | 62 | 3.9 | 0 | 0 | 0 | 0 | 0 | 0 |
| 1079 | 0 |  | 0 | 62 | 24.4 | 74.0 | 97 | 307 | 48 | 251 | 94 | 5.5 | 26.6 | 127 | 77 | 5.4 | 0 | 0 | 1 | 0 | 0 | 0 |
| 1080 | 0 |  | 1 | 44 | 18.7 | 63.0 | 69 | 224 | 59 | 155 | 40 | 5.9 | 34.6 | 106 | 69 | 4.9 | 0 | 0 | 0 | 0 | 0 | 0 |
| 1081 | 0 |  | 1 | 41 | 22.4 | 81.0 | 91 | 199 | 45 | 143 | 74 | 6.0 | 23.3 | 113 | 77 | 5.3 | 0 | 0 | 0 | 0 | 0 | 0 |
| 1082 | 0 |  | 0 | 39 | 19.8 | 65.5 | 80 | 263 | 97 | 148 | 60 | 4.7 | 18.2 | 107 | 70 | 4.7 | 0 | 0 | 0 | 0 | 0 | 0 |
| 1083 | 0 |  | 0 | 37 | 21.8 | 70.0 | 92 | 233 | 79 | 142 | 102 | 5.6 | 36.0 | 100 | 72 | 5.0 | 0 | 0 | 0 | 0 | 0 | 0 |
| 1084 | 0 |  | 0 | 54 | 24.1 | 71.0 | 86 | 246 | 75 | 159 | 47 | 5.7 | 21.0 | 107 | 70 | 5.7 | 0 | 0 | 0 | 0 | 0 | 0 |
| 1085 | 0 |  | 1 | 31 | 26.0 | 77.0 | 83 | 233 | 53 | 159 | 73 | 5.9 | 22.7 | 140 | 80 | 4.8 | 0 | 0 | 0 | 1 | 0 | 0 |
| 1086 | 1 | 1 | 1 | 62 | 27.0 | 99.0 | 103 | 140 | 32 | 102 | 47 | 6.1 | 19.5 | 111 | 67 | 5.9 | 1 | 0 | 1 | 0 | 1 | 1 |
| 1087 | 1 | 1 | 0 | 45 | 24.0 | 78.0 | 89 | 191 | 52 | 119 | 55 | 4.4 | 16.2 | 101 | 66 | 5.4 | 0 | 0 | 0 | 0 | 0 | 0 |
| 1088 | 0 |  | 0 | 60 | 25.7 | 76.0 | 79 | 251 | 50 | 183 | 89 | 7.5 | 19.5 | 116 | 75 | 5.4 | 0 | 0 | 0 | 0 | 0 | 0 |
| 1089 | 0 |  | 1 | 36 | 24.5 | 78.5 | 88 | 163 | 47 | 109 | 27 | 5.9 | 30.5 | 125 | 75 | 5.1 | 0 | 0 | 0 | 0 | 0 | 0 |
| 1090 | 0 |  | 0 | 43 | 23.8 | 83.0 | 60 | 178 | 63 | 104 | 103 | 6.7 | 20.1 | 111 | 59 | 4.6 | 1 | 0 | 0 | 0 | 0 | 0 |
| 1091 | 0 |  | 0 | 59 | 20.7 | 84.0 | 106 | 255 | 67 | 165 | 125 | 4.8 | 35.2 | 113 | 75 | 5.3 | 1 | 0 | 0 | 1 | 1 | 1 |
| 1092 | 1 | 1 | 1 | 60 | 32.5 | 102.0 | 91 | 189 | 29 | 124 | 262 | 6.2 | 13.8 | 145 | 97 | 5.5 | 1 | 1 | 1 | 1 | 0 | 1 |
| 1093 | 0 |  | 0 | 60 | 22.0 | 74.5 | 94 | 210 | 67 | 122 | 81 | 3.9 | 13.1 | 95 | 55 | 4.7 | 0 | 0 | 0 | 1 | 0 | 0 |
| 1094 | 0 |  | 0 | 52 | 20.6 | 68.0 | 82 | 231 | 61 | 140 | 97 | 4.8 | 12.6 | 116 | 86 | 5.5 | 0 | 0 | 0 | 1 | 0 | 0 |
| 1095 | 0 |  | 0 | 36 | 24.6 | 73.0 | 75 | 164 | 55 | 95 | 47 | 5.4 | 20.1 | 111 | 73 | 4.7 | 0 | 0 | 0 | 0 | 0 | 0 |
| 1096 | 0 |  | 1 | 46 | 24.7 | 78.0 | 97 | 205 | 58 | 135 | 42 | 4.3 | 41.6 | 126 | 68 | 5.7 | 0 | 0 | 0 | 0 | 0 | 0 |
| 1097 | 0 |  | 1 | 53 | 22.3 | 83.0 | 89 | 205 | 41 | 144 | 95 | 5.7 | 13.8 | 128 | 77 | 5.3 | 0 | 0 | 0 | 0 | 0 | 0 |
| 1098 | 0 |  | 0 | 42 | 26.2 | 80.0 | 87 | 160 | 49 | 105 | 92 | 5.0 | 17.1 | 119 | 85 | 5.5 | 1 | 0 | 1 | 1 | 0 | 1 |
| 1099 | 0 |  | 1 | 61 | 23.8 | 83.0 | 88 | 194 | 51 | 129 | 57 | 7.4 | 30.2 | 125 | 79 | 5.2 | 0 | 0 | 0 | 0 | 0 | 0 |
| 1100 | 1 | 0 | 1 | 61 | 28.2 | 94.0 | 95 | 192 | 46 | 124 | 108 | 6.8 | 30.7 | 116 | 74 | 5.9 | 1 | 0 | 0 | 0 | 1 | 0 |
| 1101 | 1 | 1 | 1 | 40 | 23.8 | 84.0 | 87 | 276 | 59 | 170 | 269 | 7.0 | 29.0 | 128 | 85 | 5.6 | 0 | 1 | 0 | 1 | 0 | 0 |
| 1102 | 1 | 0 | 0 | 32 | 23.5 | 73.0 | 91 | 167 | 47 | 109 | 61 | 4.3 | 24.6 | 102 | 67 | 5.3 | 0 | 0 | 1 | 0 | 0 | 0 |
| 1103 | 0 |  | 0 | 43 | 22.2 | 79.0 | 99 | 187 | 47 | 118 | 96 | 4.8 | 13.6 | 95 | 59 | 5.3 | 0 | 0 | 1 | 1 | 0 | 0 |
| 1104 | 0 |  | 0 | 57 | 20.0 | 70.0 | 79 | 228 | 53 | 158 | 95 | 5.8 | 22.9 | 106 | 73 | 5.7 | 0 | 0 | 0 | 0 | 0 | 0 |
| 1105 | 1 | 0 | 1 | 58 | 32.2 | 98.0 | 85 | 158 | 26 | 86 | 243 | 7.3 | 32.3 | 119 | 89 | 5.8 | 1 | 1 | 1 | 1 | 1 | 1 |
| 1106 | 0 |  | 0 | 43 | 21.7 | 67.0 | 94 | 237 | 44 | 133 | 318 | 4.4 | 59.8 | 120 | 62 | 5.8 | 0 | 1 | 1 | 0 | 1 | 1 |
| 1107 | 1 | 1 | 1 | 51 | 29.9 | 89.0 | 158 | 214 | 44 | 160 | 145 | 4.4 | 20.8 | 127 | 80 | 7.4 | 0 | 0 | 0 | 0 | 1 | 0 |
| 1108 | 0 |  | 1 | 31 | 41.6 | 126.0 | 100 | 164 | 43 | 117 | 60 | 6.5 | 25.5 | 153 | 92 | 4.9 | 1 | 0 | 0 | 1 | 1 | 1 |
| 1109 | 0 |  | 1 | 41 | 25.8 | 89.0 | 103 | 198 | 59 | 123 | 95 | 3.8 | 23.3 | 116 | 75 | 5.4 | 0 | 0 | 0 | 0 | 1 | 0 |
| 1110 | 0 |  | 0 | 56 | 20.7 | 70.0 | 83 | 233 | 70 | 152 | 43 | 3.9 | 27.2 | 107 | 68 | 5.7 | 0 | 0 | 0 | 0 | 0 | 0 |
| 1111 | 0 |  | 0 | 60 | 21.7 | 76.5 | 99 | 272 | 62 | 185 | 87 | 4.6 | 76.8 | 102 | 68 | 5.7 | 0 | 0 | 0 | 0 | 0 | 0 |
| 1112 | 0 |  | 0 | 48 | 24.3 | 79.0 | 96 | 158 | 29 | 103 | 126 | 4.3 | 19.5 | 111 | 68 | 5.5 | 0 | 0 | 1 | 0 | 0 | 0 |
| 1113 | 0 |  | 0 | 53 | 24.0 | 73.0 | 108 | 288 | 70 | 179 | 176 | 4.5 | 32.5 | 112 | 74 | 5.9 | 0 | 1 | 0 | 0 | 1 | 0 |
| 1114 | 0 |  | 1 | 47 | 29.9 | 95.0 | 95 | 194 | 42 | 139 | 103 | 7.2 | 37.9 | 140 | 98 | 5.2 | 1 | 0 | 0 | 1 | 0 | 0 |
| 1115 | 1 | 1 | 0 | 62 | 21.8 | 69.0 | 94 | 236 | 63 | 159 | 80 | 3.9 | 25.8 | 128 | 76 | 5.5 | 0 | 0 | 0 | 0 | 0 | 0 |
| 1116 | 0 |  | 1 | 56 | 28.1 | 84.0 | 89 | 251 | 48 | 150 | 181 | 8.5 | 25.9 | 119 | 77 | 5.7 | 0 | 1 | 0 | 1 | 0 | 0 |
| 1117 | 0 |  | 0 | 51 | 29.1 | 77.5 | 101 | 186 | 59 | 127 | 70 | 5.5 | 35.5 | 124 | 79 | 5.6 | 0 | 0 | 0 | 1 | 1 | 0 |
| 1118 | 0 |  | 0 | 39 | 24.2 | 63.0 | 95 | 172 | 51 | 111 | 40 | 3.8 | 16.4 | 109 | 71 | 5.1 | 0 | 0 | 0 | 0 | 0 | 0 |
| 1119 | 0 |  | 1 | 35 | 24.5 | 89.0 | 82 | 210 | 35 | 156 | 132 | 6.9 | 16.1 | 108 | 68 | 5.2 | 0 | 0 | 1 | 0 | 0 | 0 |
| 1120 | 0 |  | 1 | 33 | 23.6 | 77.0 | 94 | 169 | 33 | 73 | 328 | 6.4 | 13.9 | 128 | 74 | 5.1 | 0 | 1 | 1 | 0 | 0 | 0 |
| 1121 | 1 | 1 | 1 | 57 | 18.3 | 78.0 | 93 | 195 | 45 | 139 | 100 | 6.8 | 20.6 | 153 | 93 | 5.2 | 0 | 0 | 0 | 1 | 0 | 0 |
| 1122 | 0 |  | 0 | 41 | 24.0 | 76.0 | 88 | 178 | 54 | 119 | 33 | 4.3 | 19.1 | 108 | 70 | 5.2 | 0 | 0 | 0 | 0 | 0 | 0 |
| 1123 | 0 |  | 1 | 64 | 23.3 | 78.0 | 99 | 180 | 40 | 134 | 102 | 5.4 | 60.7 | 140 | 92 | 5.5 | 0 | 0 | 0 | 1 | 0 | 0 |
| 1124 | 0 |  | 1 | 40 | 19.9 | 72.5 | 92 | 181 | 59 | 113 | 73 | 4.7 | 22.6 | 126 | 75 | 5.2 | 0 | 0 | 0 | 0 | 0 | 0 |
| 1125 | 0 |  | 0 | 56 | 22.6 | 80.0 | 95 | 228 | 51 | 132 | 262 | 5.0 | 26.4 | 148 | 83 | 5.2 | 1 | 1 | 0 | 1 | 0 | 1 |
| 1126 | 1 | 1 | 1 | 59 | 26.1 | 89.0 | 104 | 174 | 33 | 130 | 120 | 7.3 | 25.0 | 119 | 73 | 5.8 | 0 | 0 | 1 | 0 | 1 | 0 |
| 1127 | 0 |  | 1 | 56 | 24.0 | 83.0 | 91 | 157 | 31 | 92 | 222 | 4.6 | 20.8 | 121 | 73 | 5.3 | 0 | 1 | 1 | 0 | 0 | 0 |
| 1128 | 1 | 1 | 0 | 53 | 20.6 | 68.0 | 82 | 203 | 90 | 101 | 32 | 5.3 | 19.1 | 118 | 82 | 5.2 | 0 | 0 | 0 | 0 | 0 | 0 |
| 1129 | 1 | 1 | 1 | 51 | 30.4 | 95.0 | 117 | 246 | 37 | 156 | 260 | 6.2 | 43.3 | 132 | 79 | 6.3 | 1 | 1 | 1 | 1 | 1 | 1 |
| 1130 | 1 | 1 | 0 | 47 | 24.2 | 85.0 | 85 | 178 | 38 | 102 | 270 | 6.9 | 20.6 | 141 | 88 | 5.2 | 1 | 1 | 1 | 1 | 0 | 1 |
| 1131 | 0 |  | 0 | 63 | 20.8 | 70.0 | 96 | 215 | 57 | 147 | 30 | 5.2 | 23.4 | 128 | 74 | 5.9 | 0 | 0 | 0 | 1 | 1 | 0 |
| 1132 | 0 |  | 1 | 53 | 26.9 | 90.0 | 142 | 185 | 33 | 93 | 312 | 8.8 | 55.1 | 133 | 88 | 7.3 | 1 | 1 | 1 | 1 | 1 | 1 |
| 1133 | 0 |  | 0 | 37 | 21.3 | 64.0 | 93 | 162 | 75 | 68 | 76 | 4.2 | 32.9 | 109 | 71 | 5.1 | 0 | 0 | 0 | 0 | 0 | 0 |
| 1134 | 0 |  | 0 | 37 | 21.7 | 66.0 | 83 | 180 | 66 | 105 | 38 | 2.4 | 30.3 | 104 | 68 | 5.6 | 0 | 0 | 0 | 0 | 0 | 0 |
| 1135 | 0 |  | 0 | 44 | 22.5 | 79.0 | 104 | 181 | 40 | 126 | 115 | 5.0 | 14.0 | 120 | 74 | 5.3 | 0 | 0 | 1 | 0 | 1 | 0 |
| 1136 | 1 | 0 | 1 | 45 | 32.3 | 112.0 | 155 | 210 | 47 | 145 | 157 | 4.8 | 29.5 | 166 | 111 | 7.2 | 1 | 1 | 0 | 1 | 1 | 1 |
| 1137 | 0 |  | 0 | 53 | 22.2 | 82.5 | 95 | 164 | 34 | 107 | 123 | 4.3 | 34.9 | 100 | 61 | 5.2 | 1 | 0 | 1 | 0 | 0 | 0 |
| 1138 | 0 |  | 1 | 35 | 28.3 | 85.0 | 93 | 193 | 38 | 134 | 192 | 6.9 | 28.3 | 118 | 77 | 5.2 | 0 | 1 | 1 | 0 | 0 | 0 |
| 1139 | 1 | 1 | 1 | 61 | 26.3 | 86.0 | 100 | 214 | 49 | 158 | 51 | 5.4 | 31.0 | 124 | 83 | 5.7 | 0 | 0 | 0 | 0 | 1 | 0 |
| 1140 | 0 |  | 1 | 61 | 27.0 | 92.0 | 123 | 192 | 56 | 104 | 174 | 8.5 | 38.6 | 191 | 96 | 6.0 | 1 | 1 | 0 | 1 | 1 | 1 |
| 1141 | 0 |  | 0 | 48 | 22.9 | 77.0 | 88 | 204 | 78 | 110 | 32 | 5.3 | 17.8 | 116 | 68 | 5.3 | 0 | 0 | 0 | 0 | 0 | 0 |
| 1142 | 0 |  | 0 | 53 | 31.4 | 94.5 | 95 | 208 | 49 | 140 | 104 | 6.0 | 11.4 | 103 | 73 | 5.4 | 1 | 0 | 1 | 1 | 0 | 1 |
| 1143 | 0 |  | 0 | 50 | 21.8 | 68.5 | 87 | 216 | 69 | 131 | 80 | 4.9 | 47.2 | 108 | 65 | 5.4 | 0 | 0 | 0 | 0 | 0 | 0 |
| 1144 | 0 |  | 1 | 58 | 27.1 | 92.5 | 100 | 120 | 46 | 68 | 98 | 7.7 | 36.2 | 111 | 60 | 5.9 | 1 | 0 | 0 | 0 | 1 | 0 |
| 1145 | 1 | 0 | 0 | 31 | 18.6 | 60.0 | 71 | 141 | 52 | 86 | 56 | 3.5 | 20.1 | 105 | 67 | 4.3 | 0 | 0 | 0 | 0 | 0 | 0 |
| 1146 | 0 |  | 1 | 49 | 23.9 | 85.5 | 94 | 205 | 47 | 142 | 55 | 6.5 | 27.1 | 124 | 79 | 5.8 | 0 | 0 | 0 | 1 | 1 | 0 |
| 1147 | 0 |  | 0 | 53 | 22.2 | 76.0 | 105 | 244 | 74 | 147 | 115 | 3.8 | 12.8 | 129 | 75 | 5.7 | 0 | 0 | 0 | 0 | 1 | 0 |
| 1148 | 1 | 1 | 1 | 43 | 30.5 | 101.0 | 236 | 259 | 44 | 175 | 314 | 6.1 | 20.6 | 126 | 89 | 9.3 | 1 | 1 | 0 | 1 | 1 | 1 |
| 1149 | 0 |  | 0 | 58 | 21.6 | 78.0 | 111 | 149 | 29 | 95 | 141 | 6.6 | 26.6 | 122 | 68 | 4.9 | 0 | 0 | 1 | 0 | 1 | 0 |
| 1150 | 0 |  | 1 | 55 | 24.0 | 80.0 | 104 | 214 | 37 | 153 | 191 | 7.0 | 32.9 | 109 | 65 | 5.8 | 0 | 1 | 1 | 0 | 1 | 1 |
| 1151 | 0 |  | 0 | 31 | 18.9 | 63.0 | 73 | 126 | 61 | 44 | 45 | 4.5 | 13.0 | 104 | 73 | 5.1 | 0 | 0 | 0 | 0 | 0 | 0 |
| 1152 | 0 |  | 1 | 53 | 23.0 | 81.0 | 96 | 280 | 40 | 190 | 291 | 7.5 | 28.2 | 129 | 87 | 4.7 | 0 | 1 | 0 | 1 | 0 | 0 |
| 1153 | 0 |  | 1 | 48 | 22.2 | 78.0 | 155 | 237 | 61 | 167 | 48 | 4.4 | 17.0 | 117 | 79 | 9.7 | 0 | 0 | 0 | 0 | 1 | 0 |
| 1154 | 0 |  | 0 | 40 | 25.3 | 75.0 | 94 | 179 | 63 | 108 | 69 | 3.7 | 23.5 | 119 | 83 | 5.4 | 0 | 0 | 0 | 0 | 0 | 0 |
| 1155 | 0 |  | 1 | 61 | 21.7 | 76.0 | 95 | 153 | 39 | 98 | 97 | 7.3 | 38.8 | 112 | 73 | 5.5 | 0 | 0 | 1 | 0 | 0 | 0 |
| 1156 | 1 | 1 | 1 | 56 | 29.5 | 97.0 | 102 | 131 | 62 | 55 | 75 | 7.4 | 25.3 | 128 | 81 | 6.0 | 1 | 0 | 0 | 1 | 1 | 1 |
| 1157 | 0 |  | 1 | 47 | 28.3 | 83.0 | 87 | 181 | 55 | 120 | 40 | 7.2 | 45.6 | 137 | 82 | 5.0 | 0 | 0 | 0 | 1 | 0 | 0 |
| 1158 | 0 |  | 0 | 49 | 22.0 | 71.0 | 97 | 188 | 50 | 132 | 69 | 4.7 | 21.1 | 115 | 65 | 5.4 | 0 | 0 | 0 | 0 | 0 | 0 |
| 1159 | 0 |  | 1 | 57 | 27.6 | 93.0 | 99 | 205 | 42 | 127 | 148 | 7.8 | 46.8 | 119 | 80 | 5.3 | 1 | 0 | 0 | 0 | 0 | 0 |
| 1160 | 1 | 1 | 0 | 65 | 24.6 | 81.0 | 107 | 204 | 81 | 111 | 61 | 6.3 | 19.5 | 123 | 70 | 6.2 | 1 | 0 | 0 | 0 | 1 | 0 |
| 1161 | 0 |  | 1 | 66 | 24.7 | 86.0 | 90 | 174 | 36 | 121 | 86 | 5.2 | 28.7 | 115 | 71 | 5.5 | 0 | 0 | 1 | 0 | 0 | 0 |
| 1162 | 1 | 0 | 0 | 74 | 28.2 | 93.0 | 92 | 215 | 56 | 120 | 86 | 5.3 | 8.8 | 153 | 82 | 5.5 | 1 | 0 | 0 | 1 | 0 | 0 |
| 1163 | 0 |  | 1 | 66 | 28.8 | 94.0 | 105 | 196 | 70 | 103 | 116 | 6.3 | 25.1 | 159 | 105 | 5.7 | 1 | 0 | 0 | 1 | 1 | 1 |
| 1164 | 1 | 1 | 0 | 70 | 25.3 | 84.0 | 70 | 201 | 73 | 110 | 50 | 6.9 | 27.9 | 120 | 68 | 5.2 | 1 | 0 | 0 | 0 | 0 | 0 |
| 1165 | 1 | 1 | 1 | 68 | 25.0 | 82.0 | 92 | 206 | 47 | 137 | 89 | 6.0 | 15.8 | 136 | 79 | 5.7 | 0 | 0 | 0 | 1 | 0 | 0 |
| 1166 | 1 | 1 | 0 | 66 | 23.4 | 78.0 | 103 | 149 | 54 | 80 | 84 | 6.1 | 31.2 | 151 | 75 | 6.3 | 0 | 0 | 0 | 1 | 1 | 0 |
| 1167 | 1 | 1 | 1 | 69 | 24.6 | 86.5 | 90 | 118 | 40 | 64 | 76 | 5.4 | 37.9 | 129 | 75 | 6.0 | 0 | 0 | 0 | 1 | 1 | 0 |
| 1168 | 1 | 1 | 1 | 67 | 26.3 | 84.5 | 97 | 202 | 43 | 140 | 105 | 4.8 | 22.6 | 119 | 77 | 5.4 | 0 | 0 | 0 | 1 | 0 | 0 |
| 1169 | 0 |  | 1 | 72 | 24.8 | 97.0 | 113 | 138 | 41 | 71 | 130 | 6.4 | 32.1 | 129 | 77 | 6.0 | 1 | 0 | 0 | 1 | 1 | 1 |
| 1170 | 1 | 1 | 0 | 65 | 22.8 | 79.5 | 86 | 213 | 61 | 132 | 81 | 4.5 | 31.7 | 135 | 80 | 5.1 | 0 | 0 | 0 | 1 | 0 | 0 |
| 1171 | 1 | 0 | 1 | 66 | 24.5 | 90.0 | 102 | 154 | 38 | 92 | 94 | 6.9 | 16.9 | 115 | 73 | 5.5 | 1 | 0 | 1 | 0 | 1 | 1 |
| 1172 | 0 |  | 0 | 66 | 25.1 | 80.0 | 90 | 219 | 52 | 145 | 91 | 4.5 | 25.3 | 115 | 70 | 5.3 | 1 | 0 | 0 | 0 | 0 | 0 |
| 1173 | 0 |  | 0 | 69 | 25.8 | 81.0 | 122 | 177 | 71 | 89 | 102 | 6.3 | 32.8 | 166 | 92 | 5.8 | 1 | 0 | 0 | 1 | 1 | 1 |
| 1174 | 0 |  | 0 | 67 | 26.5 | 88.0 | 82 | 149 | 42 | 98 | 75 | 5.2 | 31.9 | 163 | 88 | 5.1 | 1 | 0 | 1 | 1 | 0 | 1 |
| 1175 | 1 | 1 | 0 | 67 | 24.3 | 79.0 | 107 | 176 | 63 | 87 | 102 | 6.0 | 20.8 | 135 | 80 | 6.4 | 0 | 0 | 0 | 1 | 1 | 0 |
| 1176 | 0 |  | 0 | 66 | 22.6 | 80.0 | 89 | 163 | 67 | 85 | 61 | 5.3 | 38.9 | 123 | 74 | 5.6 | 1 | 0 | 0 | 0 | 0 | 0 |
| 1177 | 1 | 1 | 1 | 66 | 29.7 | 96.0 | 130 | 166 | 50 | 108 | 126 | 7.2 | 30.7 | 126 | 79 | 6.4 | 1 | 0 | 0 | 1 | 1 | 1 |
| 1178 | 0 |  | 0 | 66 | 22.2 | 75.5 | 99 | 183 | 78 | 77 | 86 | 5.2 | 25.8 | 145 | 77 | 5.2 | 0 | 0 | 0 | 1 | 0 | 0 |
| 1179 | 1 | 1 | 1 | 66 | 25.5 | 81.0 | 91 | 187 | 71 | 106 | 34 | 6.5 | 46.9 | 128 | 78 | 5.4 | 0 | 0 | 0 | 0 | 0 | 0 |
| 1180 | 1 | 1 | 1 | 68 | 25.1 | 78.0 | 93 | 155 | 49 | 88 | 43 | 7.4 | 25.6 | 126 | 74 | 5.6 | 0 | 0 | 0 | 1 | 0 | 0 |
| 1181 | 0 |  | 0 | 66 | 23.5 | 86.0 | 108 | 186 | 43 | 116 | 104 | 4.6 | 35.1 | 127 | 76 | 6.2 | 1 | 0 | 1 | 0 | 1 | 1 |
| 1182 | 0 |  | 0 | 66 | 21.4 | 73.0 | 95 | 225 | 67 | 141 | 56 | 5.6 | 51.4 | 113 | 59 | 5.3 | 0 | 0 | 0 | 0 | 0 | 0 |
| 1183 | 1 | 1 | 0 | 68 | 25.7 | 89.0 | 155 | 133 | 64 | 52 | 74 | 5.4 | 33.2 | 114 | 67 | 6.4 | 1 | 0 | 0 | 0 | 1 | 0 |
| 1184 | 0 |  | 0 | 66 | 25.3 | 82.0 | 89 | 192 | 65 | 117 | 38 | 6.4 | 43.3 | 134 | 60 | 5.5 | 1 | 0 | 0 | 1 | 0 | 0 |
| 1185 | 0 |  | 0 | 68 | 24.3 | 75.0 | 90 | 148 | 50 | 81 | 66 | 8.6 | 34.7 | 130 | 78 | 5.6 | 0 | 0 | 0 | 1 | 0 | 0 |
| 1186 | 1 | 1 | 1 | 66 | 22.1 | 76.5 | 102 | 202 | 28 | 149 | 154 | 6.5 | 28.3 | 112 | 70 | 5.6 | 0 | 1 | 1 | 0 | 1 | 1 |
| 1187 | 1 | 1 | 1 | 65 | 27.4 | 85.5 | 73 | 224 | 44 | 162 | 37 | 8.1 | 45.8 | 115 | 73 | 4.9 | 0 | 0 | 0 | 0 | 0 | 0 |
| 1188 | 1 | 0 | 1 | 70 | 27.5 | 94.0 | 133 | 138 | 33 | 84 | 173 | 7.5 | 33.8 | 129 | 76 | 8.1 | 1 | 1 | 1 | 0 | 1 | 1 |
| 1189 | 0 |  | 1 | 66 | 21.7 | 82.0 | 100 | 214 | 36 | 162 | 79 | 5.8 | 53.3 | 133 | 80 | 5.2 | 0 | 0 | 1 | 1 | 1 | 1 |
| 1190 | 0 |  | 0 | 68 | 28.5 | 95.0 | 98 | 225 | 59 | 147 | 77 | 4.9 | 21.1 | 139 | 80 | 5.8 | 1 | 0 | 0 | 1 | 1 | 1 |
| 1191 | 0 |  | 0 | 66 | 28.5 | 85.0 | 108 | 208 | 49 | 134 | 116 | 4.5 | 21.8 | 161 | 83 | 5.5 | 1 | 0 | 1 | 1 | 1 | 1 |
| 1192 | 1 | 0 | 0 | 67 | 22.5 | 72.0 | 132 | 191 | 47 | 138 | 116 | 5.5 | 13.5 | 167 | 86 | 6.7 | 0 | 0 | 1 | 1 | 1 | 1 |
| 1193 | 1 | 1 | 1 | 66 | 31.8 | 108.5 | 116 | 225 | 28 | 133 | 228 | 7.6 | 19.8 | 178 | 81 | 5.0 | 1 | 1 | 1 | 1 | 1 | 1 |
| 1194 | 0 |  | 0 | 66 | 24.4 | 78.0 | 108 | 175 | 57 | 94 | 77 | 3.3 | 36.2 | 124 | 74 | 5.6 | 0 | 0 | 0 | 1 | 1 | 0 |
| 1195 | 0 |  | 0 | 70 | 27.2 | 83.5 | 85 | 163 | 56 | 91 | 102 | 4.5 | 30.7 | 129 | 68 | 5.3 | 1 | 0 | 0 | 0 | 0 | 0 |
| 1196 | 1 | 0 | 1 | 67 | 26.6 | 86.0 | 113 | 252 | 35 | 126 | 352 | 7.0 | 46.6 | 143 | 94 | 5.7 | 0 | 1 | 1 | 1 | 1 | 1 |
| 1197 | 0 |  | 0 | 72 | 23.1 | 77.0 | 82 | 207 | 59 | 132 | 45 | 4.8 | 27.2 | 116 | 64 | 4.9 | 0 | 0 | 0 | 0 | 0 | 0 |
| 1198 | 0 |  | 0 | 67 | 24.0 | 78.0 | 84 | 281 | 51 | 217 | 50 | 5.8 | 41.6 | 119 | 67 | 5.5 | 0 | 0 | 0 | 0 | 0 | 0 |
| 1199 | 0 |  | 0 | 68 | 25.9 | 74.0 | 94 | 222 | 37 | 164 | 131 | 5.5 | 33.3 | 130 | 77 | 5.7 | 0 | 0 | 1 | 1 | 0 | 0 |
| 1200 | 1 | 1 | 0 | 72 | 22.4 | 84.0 | 95 | 154 | 26 | 78 | 251 | 5.7 | 25.8 | 142 | 70 | 4.6 | 1 | 1 | 1 | 1 | 0 | 1 |
| 1201 | 1 | 1 | 0 | 65 | 23.8 | 96.0 | 96 | 192 | 66 | 117 | 77 | 4.1 | 19.5 | 138 | 81 | 5.1 | 1 | 0 | 0 | 1 | 0 | 0 |
| 1202 | 0 |  | 0 | 68 | 29.4 | 93.0 | 97 | 154 | 39 | 85 | 122 | 6.2 | 32.7 | 148 | 93 | 6.3 | 1 | 0 | 1 | 1 | 1 | 1 |
| 1203 | 1 | 0 | 0 | 66 | 26.4 | 85.0 | 95 | 209 | 46 | 89 | 292 | 5.5 | 18.9 | 137 | 77 | 5.4 | 1 | 1 | 1 | 1 | 0 | 1 |
| 1204 | 1 | 1 | 1 | 67 | 27.9 | 95.0 | 151 | 235 | 36 | 162 | 199 | 5.9 | 42.5 | 109 | 68 | 9.7 | 1 | 1 | 1 | 0 | 1 | 1 |
| 1205 | 0 |  | 0 | 67 | 27.3 | 98.0 | 92 | 143 | 53 | 82 | 56 | 4.5 | 26.3 | 152 | 91 | 5.6 | 1 | 0 | 0 | 1 | 0 | 0 |
| 1206 | 1 | 1 | 0 | 66 | 32.6 | 93.0 | 143 | 204 | 46 | 123 | 106 | 5.8 | 24.0 | 148 | 76 | 6.2 | 1 | 0 | 1 | 1 | 1 | 1 |
| 1207 | 1 | 1 | 1 | 71 | 24.4 | 83.0 | 99 | 126 | 52 | 61 | 54 | 7.8 | 29.2 | 134 | 86 | 5.9 | 0 | 0 | 0 | 1 | 1 | 0 |
| 1208 | 1 | 1 | 1 | 66 | 33.0 | 104.0 | 101 | 183 | 43 | 121 | 93 | 6.9 | 39.9 | 121 | 73 | 5.5 | 1 | 0 | 0 | 1 | 1 | 1 |
| 1209 | 1 | 1 | 1 | 66 | 18.5 | 77.0 | 100 | 197 | 65 | 123 | 40 | 5.6 | 37.2 | 125 | 73 | 6.5 | 0 | 0 | 0 | 0 | 1 | 0 |
| 1210 | 1 | 1 | 0 | 66 | 22.2 | 75.0 | 95 | 238 | 63 | 169 | 41 | 5.3 | 33.6 | 155 | 79 | 5.7 | 0 | 0 | 0 | 1 | 0 | 0 |
| 1211 | 1 | 1 | 1 | 65 | 32.2 | 107.0 | 99 | 189 | 58 | 104 | 160 | 9.1 | 11.9 | 144 | 88 | 6.1 | 1 | 1 | 0 | 1 | 1 | 1 |
| 1212 | 0 |  | 0 | 71 | 14.7 | 60.0 | 75 | 160 | 56 | 87 | 52 | 4.4 | 32.1 | 117 | 53 | 5.3 | 0 | 0 | 0 | 0 | 0 | 0 |
| 1213 | 0 |  | 0 | 67 | 20.6 | 67.5 | 94 | 184 | 62 | 99 | 101 | 4.4 | 30.6 | 145 | 76 | 5.4 | 0 | 0 | 0 | 1 | 0 | 0 |
| 1214 | 1 | 1 | 1 | 67 | 27.4 | 95.0 | 100 | 205 | 43 | 147 | 59 | 7.2 | 17.7 | 122 | 87 | 5.3 | 1 | 0 | 0 | 1 | 1 | 1 |
| 1215 | 1 | 1 | 0 | 74 | 23.7 | 77.5 | 94 | 177 | 71 | 98 | 54 | 5.1 | 31.1 | 124 | 83 | 5.1 | 0 | 0 | 0 | 0 | 0 | 0 |
| 1216 | 1 | 1 | 0 | 67 | 22.4 | 71.0 | 91 | 204 | 74 | 111 | 67 | 5.2 | 25.6 | 158 | 96 | 5.1 | 0 | 0 | 0 | 1 | 0 | 0 |
| 1217 | 1 | 0 | 1 | 70 | 23.8 | 85.0 | 98 | 139 | 33 | 96 | 102 | 10.2 | 52.0 | 124 | 73 | 5.8 | 0 | 0 | 1 | 0 | 1 | 0 |
| 1218 | 1 | 0 | 0 | 70 | 24.2 | 68.0 | 146 | 194 | 110 | 69 | 64 | 3.5 | 28.2 | 108 | 64 | 6.9 | 0 | 0 | 0 | 0 | 1 | 0 |
| 1219 | 1 | 1 | 1 | 65 | 21.5 | 81.0 | 99 | 145 | 54 | 80 | 67 | 5.5 | 31.2 | 126 | 89 | 6.2 | 0 | 0 | 0 | 1 | 1 | 0 |
| 1220 | 1 | 1 | 0 | 67 | 15.1 | 62.5 | 71 | 248 | 74 | 161 | 44 | 5.2 | 41.0 | 107 | 71 | 5.7 | 0 | 0 | 0 | 0 | 0 | 0 |
| 1221 | 1 | 0 | 1 | 70 | 25.7 | 93.0 | 104 | 255 | 36 | 178 | 190 | 8.4 | 42.5 | 140 | 92 | 6.0 | 1 | 1 | 1 | 1 | 1 | 1 |
| 1222 | 1 | 1 | 1 | 69 | 25.2 | 87.0 | 97 | 202 | 44 | 144 | 54 | 5.7 | 42.9 | 124 | 66 | 5.1 | 0 | 0 | 0 | 0 | 0 | 0 |
| 1223 | 1 | 1 | 1 | 67 | 22.1 | 76.5 | 77 | 275 | 34 | 199 | 140 | 7.3 | 37.3 | 127 | 72 | 5.2 | 0 | 0 | 1 | 0 | 0 | 0 |
| 1224 | 1 | 1 | 1 | 66 | 24.2 | 85.0 | 77 | 216 | 58 | 138 | 169 | 5.8 | 22.4 | 152 | 84 | 5.5 | 0 | 1 | 0 | 1 | 0 | 0 |
| 1225 | 1 | 1 | 1 | 68 | 24.5 | 83.0 | 93 | 124 | 41 | 74 | 57 | 6.6 | 47.6 | 147 | 89 | 5.8 | 0 | 0 | 0 | 1 | 1 | 0 |
| 1226 | 1 | 0 | 1 | 65 | 21.7 | 78.0 | 152 | 135 | 48 | 72 | 62 | 4.9 | 54.8 | 113 | 68 | 6.5 | 0 | 0 | 0 | 0 | 1 | 0 |
| 1227 | 0 |  | 1 | 66 | 28.6 | 100.0 | 99 | 171 | 51 | 81 | 121 | 6.3 | 44.1 | 168 | 79 | 5.8 | 1 | 0 | 0 | 1 | 1 | 1 |
| 1228 | 0 |  | 0 | 71 | 31.1 | 90.0 | 82 | 213 | 58 | 128 | 70 | 6.4 | 22.6 | 133 | 74 | 5.6 | 1 | 0 | 0 | 1 | 0 | 0 |
| 1229 | 1 | 0 | 1 | 66 | 23.5 | 77.5 | 95 | 229 | 56 | 151 | 74 | 8.4 | 32.8 | 100 | 71 | 5.8 | 0 | 0 | 0 | 0 | 1 | 0 |
| 1230 | 0 |  | 1 | 65 | 26.3 | 84.0 | 91 | 185 | 31 | 107 | 187 | 4.9 | 25.8 | 136 | 87 | 5.5 | 0 | 1 | 1 | 1 | 0 | 1 |
| 1231 | 1 | 1 | 1 | 74 | 27.1 | 88.0 | 95 | 190 | 36 | 127 | 171 | 6.3 | 44.6 | 127 | 69 | 5.8 | 0 | 1 | 1 | 0 | 1 | 1 |
| 1232 | 1 | 1 | 0 | 66 | 24.2 | 83.5 | 106 | 173 | 48 | 108 | 105 | 6.4 | 32.5 | 125 | 70 | 5.3 | 1 | 0 | 1 | 1 | 1 | 1 |
| 1233 | 1 | 1 | 0 | 69 | 26.5 | 82.0 | 101 | 229 | 51 | 180 | 60 | 6.1 | 26.7 | 135 | 65 | 5.5 | 1 | 0 | 0 | 1 | 1 | 1 |
| 1234 | 0 |  | 1 | 73 | 23.0 | 82.0 | 105 | 189 | 60 | 115 | 57 | 4.3 | 31.1 | 129 | 70 | 4.3 | 0 | 0 | 0 | 0 | 1 | 0 |
| 1235 | 1 | 1 | 1 | 68 | 25.4 | 87.0 | 89 | 230 | 46 | 163 | 91 | 8.8 | 41.7 | 124 | 73 | 5.8 | 0 | 0 | 0 | 1 | 1 | 0 |
| 1236 | 1 | 1 | 0 | 71 | 22.0 | 76.5 | 142 | 219 | 49 | 146 | 89 | 4.4 | 24.2 | 144 | 78 | 6.1 | 0 | 0 | 1 | 1 | 1 | 1 |
| 1237 | 1 | 0 | 1 | 65 | 23.4 | 86.0 | 143 | 163 | 37 | 98 | 172 | 7.5 | 34.4 | 135 | 93 | 8.3 | 0 | 1 | 1 | 1 | 1 | 1 |
| 1238 | 1 | 1 | 0 | 70 | 32.2 | 98.0 | 99 | 134 | 41 | 78 | 124 | 6.3 | 22.2 | 153 | 84 | 5.8 | 1 | 0 | 1 | 1 | 1 | 1 |
| 1239 | 0 |  | 1 | 66 | 25.5 | 96.0 | 179 | 233 | 33 | 127 | 372 | 5.6 | 36.7 | 136 | 76 | 7.6 | 1 | 1 | 1 | 1 | 1 | 1 |
| 1240 | 0 |  | 0 | 66 | 22.0 | 78.0 | 99 | 176 | 42 | 107 | 131 | 6.5 | 52.8 | 113 | 70 | 5.5 | 0 | 0 | 1 | 0 | 0 | 0 |
| 1241 | 1 | 1 | 1 | 68 | 22.0 | 83.0 | 102 | 121 | 30 | 77 | 110 | 6.3 | 13.5 | 110 | 74 | 6.5 | 0 | 0 | 1 | 1 | 1 | 1 |
| 1242 | 1 | 1 | 1 | 69 | 24.6 | 93.5 | 110 | 155 | 38 | 93 | 163 | 5.1 | 37.6 | 119 | 74 | 4.3 | 1 | 1 | 1 | 0 | 1 | 1 |
| 1243 | 0 |  | 1 | 69 | 24.2 | 80.5 | 87 | 184 | 43 | 118 | 85 | 4.9 | 24.1 | 119 | 73 | 5.7 | 0 | 0 | 0 | 0 | 0 | 0 |
| 1244 | 1 | 1 | 0 | 70 | 20.9 | 69.0 | 78 | 201 | 93 | 86 | 64 | 5.3 | 61.1 | 178 | 82 | 5.7 | 0 | 0 | 0 | 1 | 0 | 0 |
| 1245 | 1 | 1 | 1 | 68 | 23.0 | 85.0 | 154 | 206 | 29 | 124 | 218 | 6.2 | 31.2 | 122 | 72 | 7.7 | 0 | 1 | 1 | 1 | 1 | 1 |
| 1246 | 0 |  | 1 | 65 | 27.2 | 88.0 | 107 | 139 | 36 | 81 | 103 | 5.9 | 50.9 | 131 | 78 | 5.6 | 0 | 0 | 1 | 1 | 1 | 1 |
| 1247 | 1 | 0 | 1 | 65 | 25.8 | 83.0 | 91 | 196 | 44 | 130 | 52 | 3.9 | 37.7 | 134 | 88 | 5.3 | 0 | 0 | 0 | 1 | 0 | 0 |
| 1248 | 0 |  | 0 | 68 | 22.9 | 84.0 | 91 | 189 | 57 | 116 | 67 | 4.5 | 27.3 | 135 | 89 | 5.6 | 1 | 0 | 0 | 1 | 0 | 0 |
| 1249 | 0 |  | 1 | 67 | 29.4 | 92.0 | 113 | 163 | 46 | 116 | 146 | 4.5 | 27.0 | 119 | 76 | 6.8 | 1 | 0 | 0 | 0 | 1 | 0 |
| 1250 | 0 |  | 1 | 65 | 30.3 | 100.0 | 101 | 226 | 54 | 152 | 112 | 3.8 | 39.1 | 137 | 81 | 6.6 | 1 | 0 | 0 | 1 | 1 | 1 |
| 1251 | 1 | 0 | 0 | 66 | 25.1 | 85.0 | 103 | 257 | 43 | 164 | 145 | 5.9 | 35.0 | 111 | 63 | 6.0 | 1 | 0 | 1 | 0 | 1 | 1 |
| 1252 | 0 |  | 1 | 69 | 21.5 | 80.0 | 100 | 206 | 43 | 98 | 220 | 7.7 | 45.2 | 107 | 78 | 5.6 | 0 | 1 | 0 | 0 | 1 | 0 |
| 1253 | 0 |  | 1 | 67 | 28.7 | 99.0 | 100 | 206 | 35 | 150 | 103 | 6.5 | 43.9 | 139 | 85 | 5.4 | 1 | 0 | 1 | 1 | 1 | 1 |
| 1254 | 0 |  | 1 | 70 | 23.1 | 77.0 | 96 | 144 | 46 | 76 | 55 | 4.1 | 49.8 | 149 | 73 | 5.4 | 0 | 0 | 0 | 1 | 0 | 0 |
| 1255 | 1 | 0 | 1 | 72 | 26.3 | 87.0 | 107 | 206 | 41 | 123 | 155 | 6.1 | 34.3 | 139 | 89 | 5.3 | 0 | 1 | 0 | 1 | 1 | 1 |
| 1256 | 1 | 1 | 1 | 68 | 28.2 | 98.0 | 135 | 137 | 42 | 84 | 48 | 5.7 | 19.9 | 129 | 87 | 7.2 | 1 | 0 | 0 | 1 | 1 | 1 |
| 1257 | 1 | 0 | 0 | 71 | 31.2 | 98.0 | 117 | 112 | 41 | 45 | 141 | 5.2 | 25.2 | 130 | 68 | 6.2 | 1 | 0 | 1 | 1 | 1 | 1 |
| 1258 | 1 | 0 | 1 | 69 | 24.6 | 93.5 | 104 | 225 | 36 | 152 | 209 | 4.6 | 40.3 | 133 | 71 | 5.7 | 1 | 1 | 1 | 1 | 1 | 1 |
| 1259 | 0 |  | 0 | 74 | 23.3 | 73.0 | 133 | 227 | 56 | 146 | 115 | 3.4 | 40.4 | 163 | 63 | 6.7 | 0 | 0 | 0 | 1 | 1 | 0 |
| 1260 | 0 |  | 0 | 69 | 24.9 | 90.0 | 85 | 112 | 32 | 56 | 108 | 4.8 | 17.5 | 123 | 77 | 5.3 | 1 | 0 | 1 | 1 | 0 | 1 |
| 1261 | 1 | 1 | 1 | 67 | 28.1 | 97.0 | 94 | 169 | 32 | 113 | 85 | 6.3 | 30.0 | 105 | 66 | 6.0 | 1 | 0 | 1 | 0 | 1 | 1 |
| 1262 | 1 | 1 | 1 | 68 | 23.1 | 85.5 | 129 | 213 | 34 | 87 | 455 | 8.3 | 41.9 | 137 | 79 | 7.7 | 0 | 1 | 1 | 1 | 1 | 1 |
| 1263 | 1 | 1 | 1 | 69 | 21.6 | 80.0 | 106 | 227 | 64 | 132 | 104 | 6.2 | 23.3 | 119 | 83 | 5.5 | 0 | 0 | 0 | 0 | 1 | 0 |
| 1264 | 1 | 1 | 1 | 70 | 28.1 | 91.0 | 91 | 134 | 53 | 56 | 142 | 5.7 | 27.5 | 153 | 74 | 6.7 | 1 | 0 | 0 | 1 | 1 | 1 |
| 1265 | 1 | 1 | 1 | 67 | 30.3 | 108.0 | 95 | 149 | 38 | 93 | 112 | 6.2 | 31.2 | 138 | 83 | 5.7 | 1 | 0 | 1 | 1 | 0 | 1 |
| 1266 | 1 | 0 | 1 | 69 | 28.1 | 90.0 | 83 | 221 | 28 | 174 | 95 | 7.3 | 50.3 | 124 | 81 | 5.4 | 1 | 0 | 1 | 0 | 0 | 0 |
| 1267 | 1 | 0 | 1 | 71 | 23.3 | 92.0 | 101 | 178 | 41 | 92 | 272 | 7.8 | 24.0 | 129 | 73 | 6.2 | 1 | 1 | 0 | 0 | 1 | 1 |
| 1268 | 1 | 1 | 1 | 70 | 29.8 | 92.0 | 105 | 186 | 36 | 127 | 112 | 5.9 | 35.1 | 125 | 70 | 5.8 | 1 | 0 | 1 | 1 | 1 | 1 |
| 1269 | 0 |  | 1 | 67 | 26.3 | 83.0 | 95 | 145 | 31 | 92 | 96 | 7.2 | 32.3 | 133 | 77 | 5.6 | 0 | 0 | 1 | 1 | 0 | 0 |
| 1270 | 1 | 1 | 1 | 69 | 25.0 | 90.0 | 101 | 269 | 58 | 197 | 47 | 6.3 | 28.6 | 163 | 92 | 5.6 | 1 | 0 | 0 | 1 | 1 | 1 |
| 1271 | 1 | 1 | 0 | 65 | 20.7 | 65.0 | 136 | 260 | 76 | 162 | 59 | 5.4 | 35.0 | 123 | 80 | 6.5 | 0 | 0 | 0 | 0 | 1 | 0 |
| 1272 | 0 |  | 0 | 66 | 23.6 | 82.5 | 102 | 243 | 62 | 159 | 80 | 5.3 | 22.6 | 136 | 82 | 6.0 | 1 | 0 | 0 | 1 | 1 | 1 |
| 1273 | 1 | 1 | 1 | 65 | 23.1 | 81.0 | 113 | 150 | 52 | 81 | 128 | 5.7 | 31.5 | 156 | 88 | 5.8 | 0 | 0 | 0 | 1 | 1 | 0 |
| 1274 | 1 | 0 | 0 | 74 | 28.8 | 87.0 | 106 | 264 | 31 | 158 | 346 | 6.6 | 27.9 | 165 | 66 | 6.0 | 1 | 1 | 1 | 1 | 1 | 1 |
| 1275 | 1 | 0 | 1 | 73 | 21.7 | 85.0 | 84 | 155 | 37 | 109 | 109 | 4.0 | 46.6 | 180 | 98 | 5.4 | 0 | 0 | 1 | 1 | 0 | 0 |
| 1276 | 0 |  | 1 | 67 | 23.2 | 81.0 | 101 | 226 | 48 | 149 | 112 | 7.2 | 50.4 | 152 | 89 | 5.4 | 0 | 0 | 0 | 1 | 1 | 0 |
| 1277 | 0 |  | 1 | 66 | 22.8 | 85.0 | 102 | 223 | 59 | 152 | 89 | 6.4 | 36.8 | 143 | 80 | 6.4 | 0 | 0 | 0 | 1 | 1 | 0 |
| 1278 | 0 |  | 1 | 68 | 22.3 | 84.5 | 96 | 200 | 49 | 131 | 99 | 5.4 | 27.1 | 157 | 94 | 5.4 | 0 | 0 | 0 | 1 | 0 | 0 |
| 1279 | 1 | 0 | 1 | 67 | 27.3 | 91.5 | 194 | 216 | 28 | 157 | 218 | 6.3 | 12.7 | 134 | 67 | 11.0 | 1 | 1 | 1 | 1 | 1 | 1 |
| 1280 | 0 |  | 0 | 73 | 19.8 | 75.0 | 85 | 225 | 81 | 110 | 57 | 6.0 | 29.6 | 124 | 65 | 5.5 | 0 | 0 | 0 | 0 | 0 | 0 |
| 1281 | 1 | 1 | 1 | 69 | 25.5 | 81.0 | 126 | 176 | 42 | 110 | 139 | 6.8 | 31.1 | 126 | 72 | 6.8 | 0 | 0 | 0 | 1 | 1 | 0 |
| 1282 | 0 |  | 1 | 71 | 19.0 | 70.0 | 92 | 122 | 38 | 66 | 67 | 3.6 | 51.1 | 146 | 87 | 5.3 | 0 | 0 | 1 | 1 | 0 | 0 |
| 1283 | 0 |  | 0 | 68 | 24.3 | 76.0 | 87 | 209 | 44 | 156 | 51 | 5.7 | 36.7 | 130 | 69 | 5.1 | 0 | 0 | 1 | 1 | 0 | 0 |
| 1284 | 1 | 0 | 0 | 69 | 23.1 | 82.0 | 102 | 208 | 48 | 118 | 147 | 6.8 | 25.5 | 126 | 69 | 5.5 | 1 | 0 | 1 | 0 | 1 | 1 |
| 1285 | 1 | 0 | 1 | 70 | 27.1 | 96.0 | 86 | 182 | 45 | 122 | 69 | 8.0 | 24.7 | 120 | 72 | 5.5 | 1 | 0 | 0 | 1 | 0 | 0 |
| 1286 | 0 |  | 1 | 70 | 23.1 | 85.0 | 137 | 198 | 71 | 111 | 59 | 7.9 | 35.2 | 163 | 75 | 6.5 | 0 | 0 | 0 | 1 | 1 | 0 |
| 1287 | 1 | 0 | 0 | 65 | 34.7 | 102.0 | 140 | 115 | 40 | 58 | 99 | 6.4 | 19.5 | 141 | 79 | 6.6 | 1 | 0 | 1 | 1 | 1 | 1 |
| 1288 | 1 | 0 | 0 | 67 | 22.6 | 82.0 | 97 | 215 | 70 | 136 | 39 | 5.1 | 35.4 | 151 | 54 | 6.3 | 1 | 0 | 0 | 1 | 1 | 1 |
| 1289 | 0 |  | 0 | 71 | 21.4 | 80.0 | 90 | 165 | 56 | 97 | 56 | 4.1 | 22.3 | 124 | 81 | 5.3 | 1 | 0 | 0 | 1 | 0 | 0 |
| 1290 | 1 | 0 | 1 | 69 | 23.2 | 81.0 | 83 | 149 | 60 | 72 | 60 | 7.1 | 65.5 | 117 | 62 | 5.2 | 0 | 0 | 0 | 0 | 0 | 0 |
| 1291 | 1 | 1 | 1 | 66 | 26.8 | 83.0 | 104 | 179 | 37 | 115 | 94 | 5.4 | 29.3 | 141 | 75 | 5.6 | 0 | 0 | 1 | 1 | 1 | 1 |
| 1292 | 0 |  | 0 | 65 | 23.5 | 70.0 | 78 | 191 | 60 | 118 | 36 | 4.0 | 36.8 | 137 | 73 | 5.5 | 0 | 0 | 0 | 1 | 0 | 0 |
| 1293 | 0 |  | 0 | 69 | 34.0 | 104.5 | 106 | 196 | 53 | 122 | 95 | 6.9 | 26.1 | 117 | 78 | 6.2 | 1 | 0 | 0 | 1 | 1 | 1 |
| 1294 | 0 |  | 0 | 65 | 24.2 | 81.0 | 94 | 310 | 59 | 221 | 110 | 5.8 | 35.4 | 140 | 82 | 6.0 | 1 | 0 | 0 | 1 | 1 | 1 |
| 1295 | 1 | 1 | 1 | 65 | 24.2 | 86.0 | 101 | 176 | 29 | 113 | 202 | 6.3 | 25.2 | 120 | 78 | 7.6 | 0 | 1 | 1 | 0 | 1 | 1 |
| 1296 | 1 | 1 | 1 | 68 | 25.1 | 83.0 | 90 | 146 | 44 | 80 | 66 | 6.3 | 42.2 | 138 | 80 | 5.8 | 0 | 0 | 0 | 1 | 1 | 0 |
| 1297 | 1 | 1 | 1 | 65 | 22.9 | 72.0 | 98 | 253 | 34 | 172 | 117 | 6.5 | 29.9 | 144 | 90 | 5.4 | 0 | 0 | 1 | 1 | 0 | 0 |
| 1298 | 0 |  | 0 | 66 | 35.6 | 96.0 | 108 | 329 | 40 | 236 | 304 | 6.8 | 38.8 | 176 | 102 | 6.8 | 1 | 1 | 1 | 1 | 1 | 1 |
| 1299 | 1 | 0 | 0 | 66 | 21.4 | 73.0 | 91 | 174 | 73 | 84 | 54 | 4.7 | 26.9 | 119 | 77 | 5.6 | 0 | 0 | 0 | 0 | 0 | 0 |
| 1300 | 0 |  | 0 | 66 | 20.8 | 79.0 | 90 | 155 | 58 | 80 | 57 | 4.3 | 21.1 | 117 | 70 | 5.3 | 0 | 0 | 0 | 1 | 0 | 0 |
| 1301 | 0 |  | 0 | 65 | 21.5 | 77.0 | 90 | 240 | 55 | 178 | 73 | 6.7 | 32.5 | 151 | 79 | 5.6 | 0 | 0 | 0 | 1 | 0 | 0 |
| 1302 | 0 |  | 1 | 65 | 20.5 | 75.0 | 90 | 193 | 57 | 116 | 102 | 6.8 | 19.4 | 100 | 62 | 5.5 | 0 | 0 | 0 | 0 | 0 | 0 |
| 1303 | 0 |  | 1 | 70 | 21.9 | 83.0 | 100 | 151 | 47 | 86 | 57 | 6.6 | 26.0 | 151 | 76 | 4.4 | 0 | 0 | 0 | 1 | 1 | 0 |
| 1304 | 0 |  | 1 | 67 | 27.4 | 93.0 | 95 | 232 | 67 | 129 | 133 | 6.2 | 29.3 | 124 | 84 | 5.7 | 1 | 0 | 0 | 0 | 0 | 0 |
| 1305 | 1 | 1 | 1 | 68 | 26.3 | 84.0 | 107 | 207 | 39 | 144 | 40 | 6.2 | 42.6 | 153 | 91 | 5.5 | 0 | 0 | 1 | 1 | 1 | 1 |
| 1306 | 1 | 1 | 1 | 71 | 20.4 | 74.0 | 101 | 180 | 68 | 102 | 66 | 5.8 | 58.2 | 144 | 75 | 5.9 | 0 | 0 | 0 | 1 | 1 | 0 |
